# Revisiting an old relationship: the causal associations of the ApoB/ApoA1 ratio with cardiometabolic diseases and relative risk factors—a mendelian randomization analysis

**DOI:** 10.1186/s12933-024-02140-2

**Published:** 2024-02-03

**Authors:** Chao Fu, Dongbo Liu, Qi Liu, Xuedong Wang, Xiaoxue Ma, Hong Pan, Shi Feng, Zhao Sun, Weishen Qiao, Mengyue Yang, Shuang Gao, Hongyu Ding, Xingtao Huang, Jingbo Hou

**Affiliations:** Department of Cardiology, The Key Laboratory of Myocardial Ischemia, The Second Affiliated Hospital of Harbin Medical University, Chinese Ministry of Education, Harbin, Heilongjiang Province China

**Keywords:** Mendelian randomization, The ApoB/ApoA1 ratio, Cardiometabolic diseases and risk factors

## Abstract

**Background:**

It has been confirmed that the ApoB/ApoA1 ratio is closely associated with the incidence of cardiometabolic diseases (CMD). However, due to uncontrolled confounding factors in observational studies, the causal relationship of this association remains unclear.

**Methods:**

In this study, we extracted the ApoB/ApoA1 ratio and data on CMD and its associated risk factors from the largest European Genome-Wide Association Study. The purpose was to conduct Mendelian Randomization (MR) analysis. The causal relationship between the ApoB/ApoA1 ratio and CMD was evaluated using both univariable and multivariable MR analyses. Furthermore, bidirectional MR analysis was performed to estimate the causal relationship between the ApoB/ApoA1 ratio and risk factors for CMD. The final verification confirmed whether the ApoB/ApoA1 ratio exhibits a mediating effect in CMD and related risk factors.

**Results:**

In terms of CMD, a noteworthy correlation was observed between the increase in the ApoB/ApoA1 ratio and various CMD, including ischemic heart disease, major adverse cardiovascular events, aortic aneurysm, cerebral ischemic disease and so on (all *P*_FDR_<0.05). Meanwhile, the ApoB/ApoA1 ratio was significantly associated with CMD risk factors, such as hemoglobin A1c, fasting insulin levels, waist-to-hip ratio, sedentary behavior, and various others, demonstrating a notable causal relationship (all *P*_FDR_<0.05). Additionally, the ApoB/ApoA1 ratio played a mediating role in CMD and relative risk factors.

**Conclusions:**

This MR study provides evidence supporting the significant causal relationship between the ApoB/ApoA1 ratio and CMD and its risk factors. Moreover, it demonstrates the mediating effect of the ApoB/ApoA1 ratio in CMD and its risk factors. These findings suggest that the ApoB/ApoA1 ratio may serve as a potential indicator for identifying the risk of developing CMD in participants.

**Supplementary Information:**

The online version contains supplementary material available at 10.1186/s12933-024-02140-2.

## Introduction

Cardiometabolic diseases (CMD), a collection of disorders involving the cardiovascular system and metabolic functionalities, pose a significant threat to human health. These diseases include hypertension, hyperlipidemia, diabetes, and obesity, among others. CMD is currently among the leading causes of mortality worldwide, contributing substantially to global healthcare costs [1, 2]. Consequently, CMD prevention and suppression have emerged as noteworthy global public health concerns.

Dyslipidemia, identified as a primary risk factor for CMD [3], has led to the ongoing development of lipid-dependent cardiovascular risk markers. Low-density lipoprotein cholesterol (LDL-c) plays a vital role as a primary risk factor for cardiovascular diseases pertaining to primary and secondary disease prevention [4, 5]. However, some observational studies have found that although maintaining LDL-c levels within a favorable range, the occurrence rate of initial or recurrent atherosclerotic cardiovascular disease (ASCVD) cannot be completely prevented due to residual risk [6–9]. These residual risks mainly originate from the triglyceride-rich lipoproteins (TRLs) and cholesterol components of metabolized TRLs (commonly referred to as remnant cholesterol) [10, 11]. Non-high density lipoprotein cholesterol (non-HDL-c) is essentially comprised of remnant cholesterol and LDL-c, encompassing the total cholesterol content of all atherogenic lipoprotein particles [12]. Research suggest that non-HDL-c possesses superior predictive capabilities for cardiovascular disease risk compared to LDL-c [13, 14], specifically among patients on statin therapy [15]. Although non-HDL-c is highly correlated with apolipoprotein B (ApoB) levels, ApoB seems to be a more accurate predictor of ASCVD. ApoB, a pivotal element in addressing this issue, indicates the total number of atherosclerosis-inducing lipoprotein particles present in the bloodstream, thus displaying the atherosclerotic disease’s risk level [16, 17]. The 2019 guidelines from the European Society of Cardiology and the European Atherosclerosis Society state that ApoB provides a more accurate assessment of cardiovascular risk and adequacy of lipid-lowering therapy than LDL-c or non-HDL-c. The measurement of ApoB is more accurate, particularly at lower concentrations, compared to LDL-c or non-HDL-c [18]. Apolipoprotein A1 (ApoA1) is the primary structural protein of high-density lipoprotein cholesterol (HDL-c) and plays a key role in reverse cholesterol transport and cellular cholesterol homeostasis [19]. Concurrently, several studies indicate that the ratio of ApoB/ApoA1 can reflect the cholesterol balance between atherosclerosis and anti-atherosclerotic lipoprotein particles, and can better predict cardiovascular risk [20–23]. A multinational case-control investigation on acute myocardial infarction (AMI) patients determined that the ApoB/ApoA1 ratio surpasses all cholesterol ratios in assessing AMI risk across varied gender, age, and race demographics [22]. Additionally, Zhou et al. found that a higher ApoB/ApoA1 ratio has predictive value for the occurrence of major adverse cardiac events (MACE) in patients with acute coronary syndrome (ACS) and diabetes [24]. However, there seems to be controversy regarding this result as some observational studies have not found an association between the ApoB/ApoA1 ratio and cardiovascular events [25, 26].

Therefore, it is necessary to elucidate the causal relationship between the ApoB/ApoA1 ratio and CMD as well as its risk factors. Conventional observation studies are frequently subjected to various confounding factors, thereby undermining result accuracy. Mendelian Randomization (MR), a genetic approach utilizing single nucleotide polymorphisms (SNPs) as instrumental variables (IVs), assists in determining the causal relationship between two traits. As MR is unswayed by other confounding factors, its application helps in judging the causal link between the observed exposure and outcome, thereby reducing conventional epidemiological studies’ confounding bias.

The principal aim of this study is to evaluate the causal association between the ApoB/ApoA1 ratio and CMD, as well as its related risk factors. Furthermore, we investigate the potential mediating effect of the ApoB/ApoA1 ratio in CMD and its related risk factors.

## Method

### Research design

**Fig. 1:**
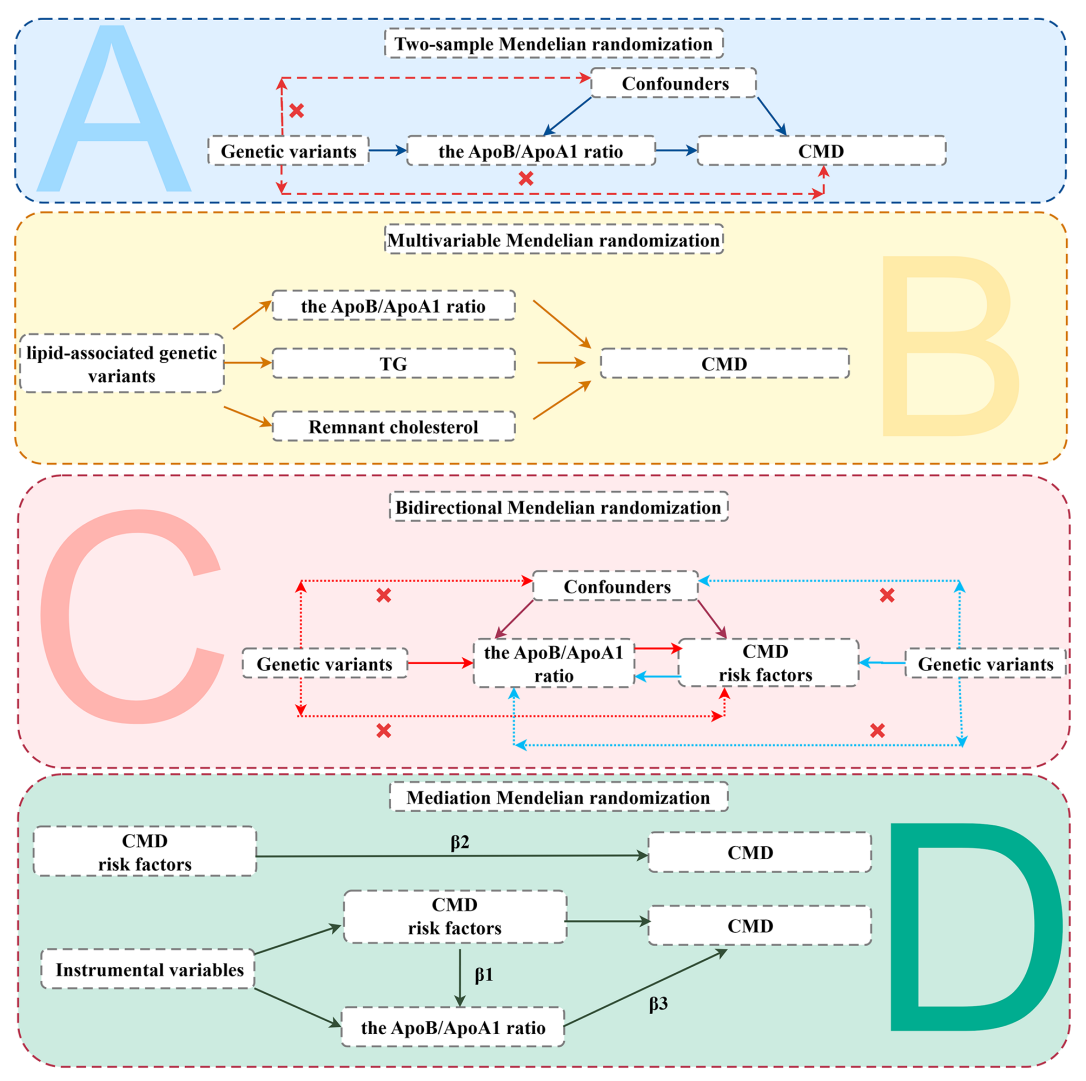
Study design. (Abbreviation: ApoB, apolipoprotein B; ApoA1, Apolipoprotein A1; CMD, cardiometabolic diseases; TG, triglyceride;)

Figure 1 showed the research design and MR assumptions of this study. This MR analysis mainly consisted of four parts. The first part investigated the causal relationship between the ApoB/ApoA1 ratio and the occurrence of CMD. The second part investigated whether the causal effect of the ApoB/ApoA1 ratio on CMD still exists after excluding the confounding of triglyceride (TG) and remnant cholesterol. The third part explored the link between the ApoB/ApoA1 ratio and CMD risk factors through a bidirectional MR analysis. The fourth part verified whether the ApoB/ApoA1 ratio mediates between CMD and its risk factors.

### Data sources of the ApoB/ApoA1 ratio

The Genome-Wide Association Study (GWAS) summary statistics for the ApoB/ApoA1 ratio were sourced from the Integrative Epidemiology Unit (IEU) Open GWAS database and included data from 115,078 individuals of European ancestry (GWAS ID: met-d-ApoB_by_ApoA1).

### Data sources of CMD

The related cases included heart failure (27,304 cases), atrial fibrillation and flutter (45,766 cases), ischemic heart disease (IHD) (63,744 cases), coronary atherosclerosis (47,550 cases), angina pectoris (34,456 cases), unstable angina pectoris (13,304 cases), myocardial infarction (MI) (24,185 cases), major adverse cardiovascular events (43,518 cases), aortic aneurysm (7,395 cases), abdominal aortic aneurysm (AAA) (3,548 cases), hypertension (111,581 cases), non-rheumatic valve diseases (20,772 cases), peripheral artery disease (PAD) (11,924 cases), pulmonary embolism (9,243 cases), cardiomyopathy (5,874 cases), intracerebral hemorrhage (3,749 cases), subarachnoid hemorrhage (3,289 cases), ischemic stroke (34,217 cases), transient ischemic attack (TIA) (18,398 cases), deep vein thrombosis (DVT) of the lower extremities (9,109 cases), type 2 diabetes mellitus (T2DM) (57,698 cases), obesity (21,375 cases), non-alcoholic fatty liver disease (NAFLD) (2,275 cases), and chronic kidney disease (CKD) (9,073 cases). All individuals in the study were of European ancestry. For more detailed information about these data sources is provided in the supplementary file in Table S1.

### Data sources of CMD risk factors

The relative risk factors included in the study were: hemoglobin A1c (HbA1c) (sample size: 46,368), fasting glucose (sample size: 133,010), fasting insulin levels (sample size: 151,013), overweight (sample size: 158,855), body mass index (BMI) (sample size: 315,347), waist circumference (sample size: 232,101), hip circumference (sample size: 213,038), waist-to-hip ratio (sample size: 458,349), LDL-c (sample size: 70,814), HDL-c (sample size: 77,409), coffee intake (sample size: 428,860), alcohol consumption (sample size: 112,117), alcohol intake frequency (sample size: 462,346), smoking (sample size: 249,752), sedentary behavior (sample size: 437,887), depression (43,280 cases), insomnia (4,214 cases), and sleep apnea (38,998 cases). All individuals in the study were of European ancestry. Detailed information about these data sources is provided in the supplementary file in Table S1.

### Selection of IVs

We identified SNPs as IVs based on three criteria: a significant genome-wide association (*p* < 5 × 10^− 8^), proven independent hereditability (r^2^ < 0.001), and absence of linkage disequilibrium (as determined by a dense window of 10,000 kb). However, due to the insufficient number of obtained SNPs, we adopted a more inclusive threshold for statistical significance in selecting IVs for the analysis of insomnia (*p* < 1 × 10^− 5^) and alcohol consumption (*p* < 5 × 10^− 6^). The assessment of weak associations between SNPs and exposures relied on Formulas F = Beta^2^/Se^2^ [27]. We deemed SNPs with an F-statistic over 10 as reliable IVs because of their capacity to lessen bias arising from weak instruments in MR analysis.

### Two-sample MR analysis

Our analysis was performed using the R software (version 4.2.1) utilizing the TwoSampleMR (version 0.5.6) and mendelian randomization pleiotropy residual sum and outlier (MR-PRESSO) (version 1.0) packages. Four different MR methods, namely, including the Inverse Variance Weighted (IVW), MR-Egger, Weighted median and MR-PRESSO methods, were applied in the Two-Sample MR analysis. The majority of our statistical assessments were performed utilizing the random-effects IVW method, known for its robustness in detecting causal relationships in two-sample MR analysis [28]. If horizontal pleiotropy was present, the MR-Egger method can correct for confounding induced by horizontal pleiotropy by adjusting the regression slope and intercept, ultimately yielding a robust estimate [29]. The Weighted median method allowed for obtaining robust estimates of treatment effects when invalid IVs did not exceed 50% [30]. In addition, the MR-PRESSO method was used to verify whether positive results still exist after removing outliers. In order to avoid the generation of false-positive results caused by multiple testing, we conducted the calculation of the false discovery rate (FDR)-adjusted *P* values in the main analyses. Significant results were defined as those with *P*_FDR_ < 0.05. Conversely, findings with *P* < 0.05 but *P*_FDR_ > 0.05 were categorized as nominally significant.

To ensure the accuracy of the experimental findings and adhere to the assumptions of MR, we conducted a comprehensive set of sensitivity analyses. These analyses encompassed the utilization of Cochran’s Q test, the implementation of MR-Egger regression, the leave-one-out method. The Cochran’s Q test served as a primary method for detecting heterogeneity. If its *P*-value was less than 0.05, it indicated significant heterogeneity [31]. The intercept of the MR-Egger regression test can provide an estimate of the degree of directional pleiotropy [29]. The leave-one-out analysis was performed to evaluate whether the significant results were driven by a single SNP. We also visually presented the heterogeneity of causal estimates through forest and funnel plots.

### Multivariable MR (MVMR)

Considering TG and remnant cholesterol, which are often discussed as lipid-related risk factors causing CMD, we used the MVMR-IVW method for MR analysis. Our aim was to determine whether the causal relationship between the ApoB/ApoA1 ratio and CMD still exists after adjusting for these possible confounding factors.

### Mediation MR analysis

We conducted a mediation analysis to examine the role of the ApoB/ApoA1 ratio in the context of CMD and associated risk factors. Initially, we assessed the effects (β1) of 16 CMD risk factors on the ApoB/ApoA1 ratio using univariable MR (UVMR). Subsequently, we investigated the effects (β2) of statistically significant CMD risk factors on the ApoB/ApoA1 ratio for 24 distinct CMD through UVMR. Finally, employing MVMR and adjusting for CMD risk factors, we evaluated the effects (β3) of the ApoB/ApoA1 ratio on the 24 different types of CMD. The mediation proportion between the ApoB/ApoA1 ratio and CMD, as well as its risk factors, was calculated as (β1 *β3)/β2. Details were shown in Fig. 1D.

## Results

The F statistics for IVs and the estimated power for all analyses were presented in Additional file, Tables S2-S4. None of these IVs had an F-statistic below the threshold of 10, suggesting limited evidence of weak instrument bias in this study. The summary information of the genetic instruments identified for the ApoB/ApoA1 ratio, CMD and CMD risk factors can be found in Additional file, Tables S2-S4.

### Causal effect of the ApoB/ApoA1 ratio on CMD from MR Analysis

**Fig. 2:**
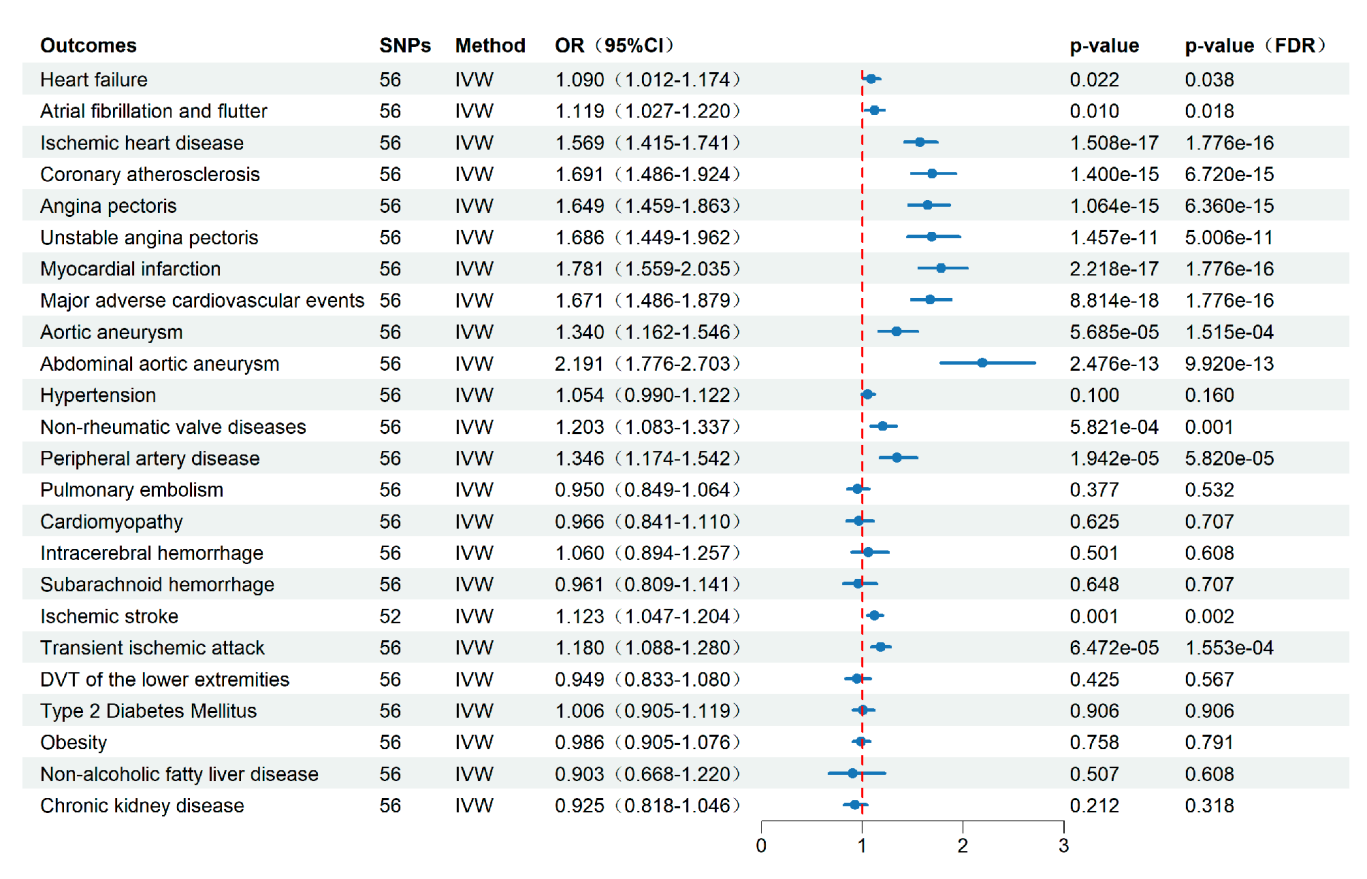
Causal effect of the ApoB/ApoA1 ratio on CMD from MR analysis. (Abbreviation: OR, Odds ratio; CI, Confidence interval; FDR, False discovery rate; DVT, Deep vein thrombosis)

The genetic prediction of the ApoB/ApoA1 ratio was significantly positively correlated with diseases like IHD, coronary atherosclerosis, angina pectoris, unstable angina pectoris, and MI. This result had been confirmed in IVW, Weighted median, MR-Egger, and MR-PRESSO analysis methods (all *P*_FDR_<0.05) (Fig. 2, Additional file, Table S5, Figure S1). On the other hand, an increase in the ApoB/ApoA1 ratio can significantly increase the incidence of MACE, which had been confirmed in all four analysis methods (*P*_FDR_<0.05) (Fig. 2, Additional file, Table S5, Figure S1). In terms of aortic aneurysm, there was a significant positive association between the ApoB/ApoA1 ratio and the incidence rates of aortic aneurysm, especially AAA (all *P*_FDR_<0.05) (Fig. 2, Additional file, Table S5, Figure S1). Also, the genetically predicted the ApoB/ApoA1 ratio had a significant positive association with the occurrence rates of PAD, non-rheumatic valve diseases, atrial fibrillation and atrial flutter (*P*_FDR_<0.05 in at least three MR methods in the four methods) (Fig. 2, Additional file, Table S5, Figure S1). Moreover, the ApoB/ApoA1 ratio was associated with the incidence rate of cerebral ischemic disease. Specifically, an elevated ApoB/ApoA1 ratio demonstrated a positive association with the occurrence rate of TIA (IVW, odds ratio (OR) = 1.180, 95% confidence interval (CI) = 1.088,1.280, *P*_FDR_=1.553e-04; MR-PRESSO, *P*_FDR_=4.233e-04) (Fig. 2, Additional file, Table S5, Figure S1). However, in other MR methods, no statistical significance has been observed (MR-Egger, OR = 1.151, 95%CI = 1.002,1.323, *P*_FDR_=0.104; Weighted median, OR = 1.080, 95%CI = 0.962,1.212, *P*_FDR_=0.257) (Fig. 2, Additional file, Table S5, Figure S1). The increase in ApoB/ApoA1 was positively correlated with the incidence rate of ischemic stroke (the *P*_FDR_-values in the analyses of IVW, Weighted median, MR-PRESSO were all significantly less than 0.05) (Fig. 2, Additional file, Table S5, Figure S1). The ApoB/ApoA1 ratio had a significant causal relationship with the incidence rate of heart failure in IVW and MR-PRESSO analysis (IVW, OR = 1.090, 95%CI = 1.012,1.174, *P*_FDR_=0.038; MR-PRESSO, *P*_FDR_=0.045) (Fig. 2, Additional file, Table S5, Figure S1), however, *P*-values were greater than 0.05 in MR Egger and Weighted median results. There was no evidence showing ApoB/ApoA1 was associated with the incidence rates of hypertension, pulmonary embolism, cardiomyopathy, intracerebral hemorrhage, subarachnoid hemorrhage, DVT of the lower extremities, T2DM, obesity, NAFLD, CKD, although the MR analysis of DVT of lower extremities, hypertension showed *P*_FDR_-values less than 0.05 in Weighted median results (Fig. 2, Additional file, Table S5, Figure S1).

### Causal effect of CMD risk factors on the ApoB/ApoA1 ratio from MR analysis

**Fig. 3:**
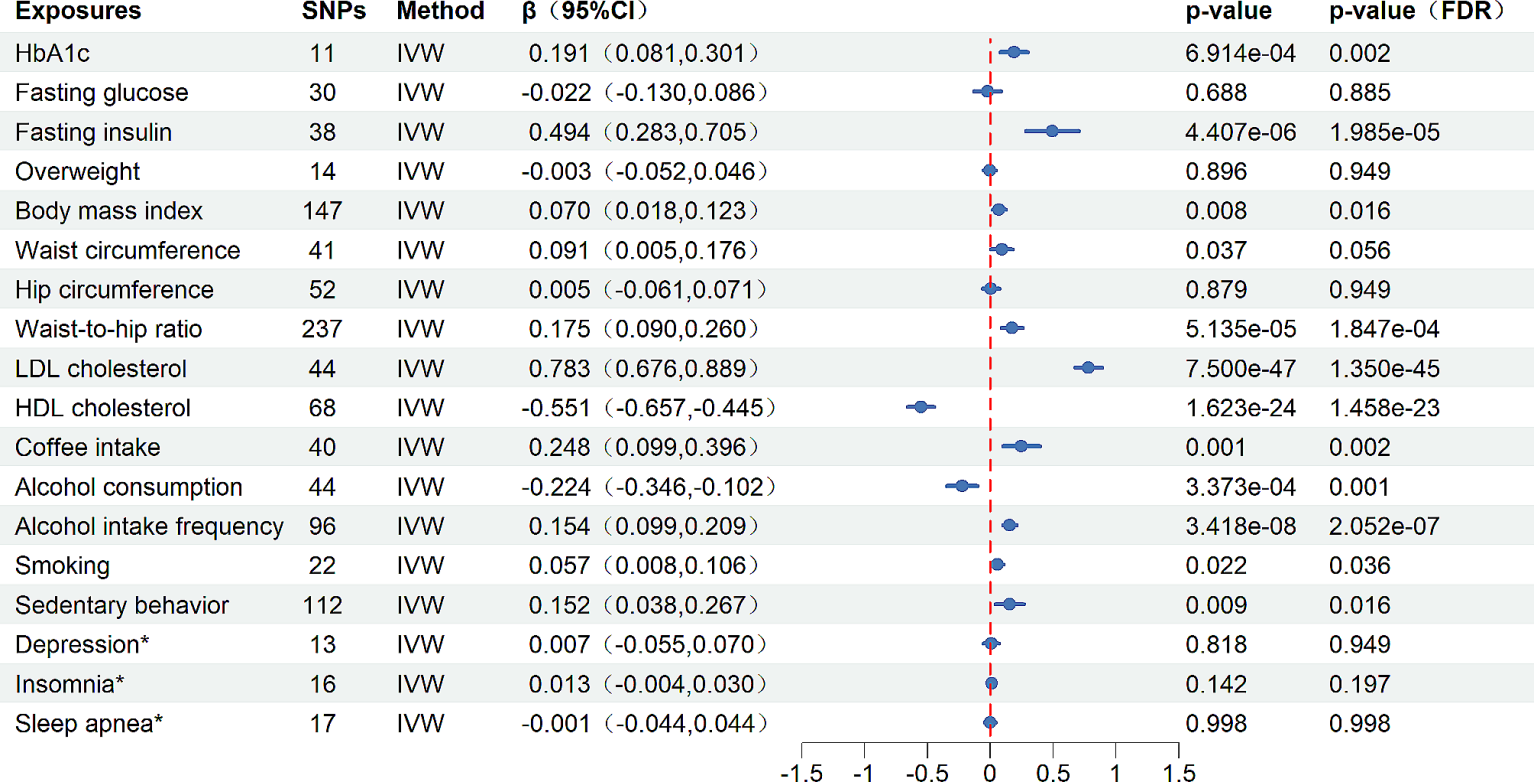
Causal effect of CMD risk factors on the ApoB/ApoA1 ratio from MR analysis. (*Represents a binary variable. Abbreviation: CI, Confidence interval; FDR, False discovery rate; HbA1c, hemoglobin A1c; LDL-c, Low-density lipoprotein cholesterol; HDL-c, High-density lipoprotein cholesterol)

The results of the MR analysis indicated that elevated levels of HbA1c, fasting insulin levels, LDL-c and waist-to-hip ratio may significantly increase the ApoB/ApoA1 ratio. It was also demonstrated that alcohol intake frequency and coffee intake can significantly contribute to the increase in the ApoB/ApoA1 ratio. However, alcohol consumption and HDL-c can significantly reduce this ratio (in at least three out of four methods the *P*_FDR_-value was less than 0.05) (Fig. 3, Additional file, Table S5, Figure S2). An increase in the ApoB/ApoA1 ratio was associated with BMI and smoking (IVW, *P*_FDR_<0.05), but this association was not statistically significant in Weighted median, MR-Egger, and MR-PRESSO methods (Fig. 3, Additional file, Table S5, Figure S2). Furthermore, there was a nominal positive correlation between waist circumference and the ApoB/ApoA1 ratio in the IVW analysis (β = 0.091, 95%CI 0.005,0.176, *P* = 0.037, *P*_FDR_=0.056), but in the Weighted median and MR-PRESSO analyses, both *P*_FDR_ values were less than 0.05 (Fig. 3, Additional file, Table S5, Figure S2). Genetic evidence also suggested that prolonged sedentary behavior may significantly increase ApoB/ApoA1 levels (IVW, β = 0.152, 95%CI 0.038,0.267, *P*_FDR_=0.016; MR-PRESSO, *P*_FDR_=0.019) (Fig. 3, Additional file, Table S5, Figure S2). However, this effect was not statistically significant in the Weighted median and MR-Egger methods. Fasting glucose, overweight, hip circumference, depression, insomnia, and sleep apnea showed no genetic association with changes in the ApoB/ApoA1 ratio (Fig. 3, Additional file, Table S5, Figure S2).

### Causal effect of the ApoB/ApoA1 ratio on CMD risk factors from MR analysis

**Fig. 4:**
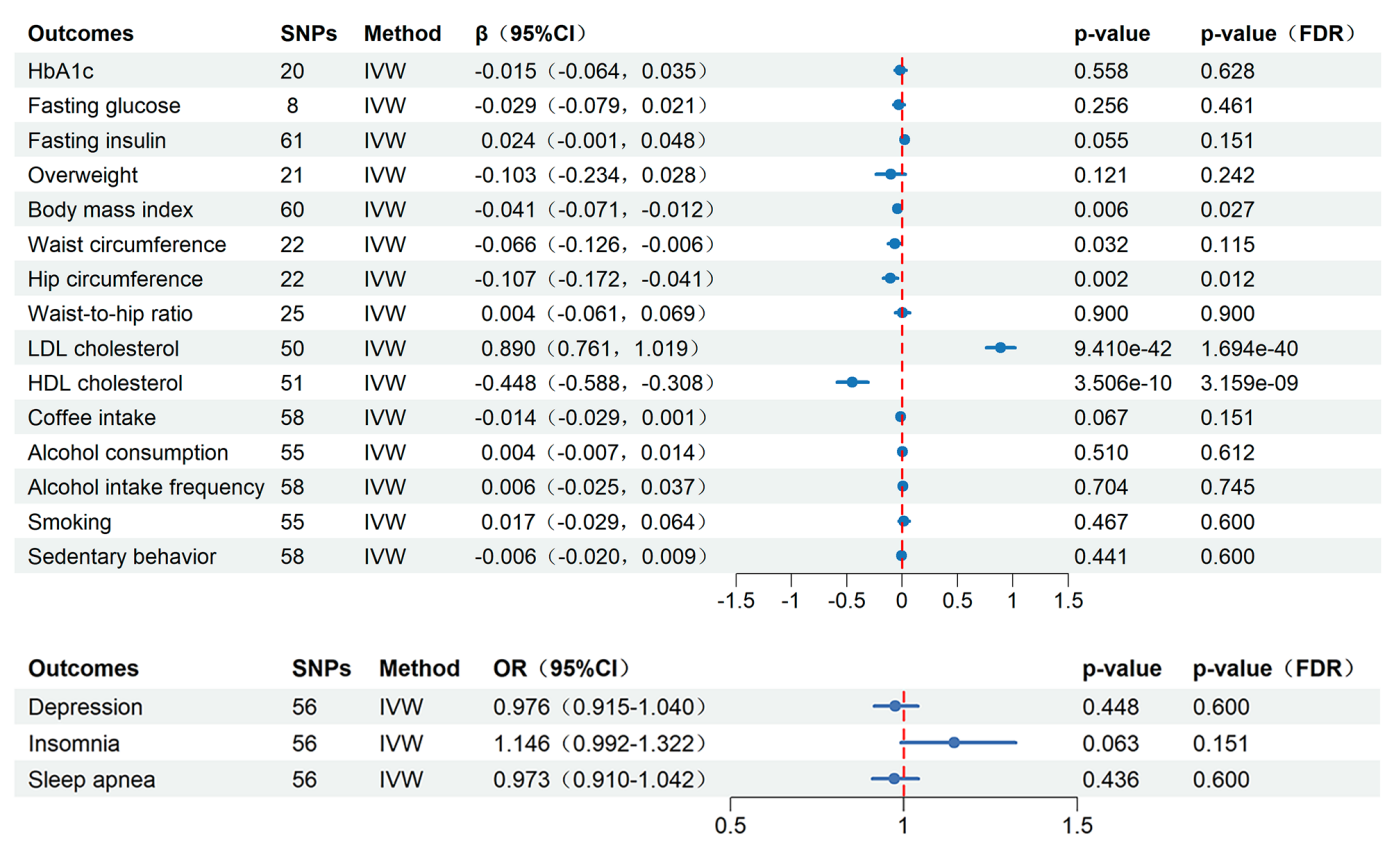
Causal effect of the ApoB/ApoA1 ratio on CMD risk factors from MR analysis. (Abbreviation: OR, Odds ratio; CI, Confidence interval; FDR, False discovery rate; HbA1c, hemoglobin A1c; LDL-c, Low-density lipoprotein cholesterol; HDL-c, High-density lipoprotein cholesterol)

The genetic prediction analysis of MR demonstrated a significant positive association between the ApoB/ApoA1 ratio and BMI, hip circumference, as well as LDL-c levels, and a negative association with HDL-c levels. This was confirmed by a *P*_FDR_-value of less than 0.05 in the IVW method, and by at least one or more *P*_FDR_-value of less than 0.05 in the Weighted median, MR-Egger, and MR-PRESSO methods (Fig. 4, Additional file, Table S5, Figure S3). In terms of waist circumference, the ApoB/ApoA1 ratio showed a nominally significant association with waist circumference (β= -0.066, 95%CI = -0.126, -0.006, *P* = 0.032, *P*_FDR_ = 0.115) in the IVW analysis.

However, the changes in the ApoB/ApoA1 ratio did not show a significant genetic association with genetic variations related to risk factors such as HbA1c, fasting glucose, fasting insulin levels, overweight, waist-to-hip ratio, coffee intake, alcohol consumption, alcohol intake frequency, smoking, depression, insomnia, sleep apnea, and sedentary behavior (Fig. 4, Additional file, Table S5, Figure S3). Considering the central obesity indicators (waist circumference, hip circumference, waist-to-hip ratio) may have a positive or reverse causal relationship with the ApoB/ApoA1 ratio. We used multivariate MR to assess whether the causal relationship holds after adjusting for different central obesity indicators. The results suggested that after adjusting for waist and hip circumference, there was a strong positive causal relationship between waist-to-hip ratio and the ApoB/ApoA1 ratio (MVMR-IVW, β = 0.180, 95%CI 0.031,0.328, *P* = 0.018). However, the causal relationships between waist circumference (MVMR-IVW, β = 0.101, 95%CI -0.222,0.425, *P* = 0.540) and hip circumference (MVMR-IVW, β=-0.037, 95%CI -0.345,0.270, *P* = 0.812) with ApoB/ApoA1 disappeared. (Additional file, Table S6).

### Causal effect of the ApoB/ApoA1 ratio on CMD from multivariable MR analysis

**Fig. 5:**
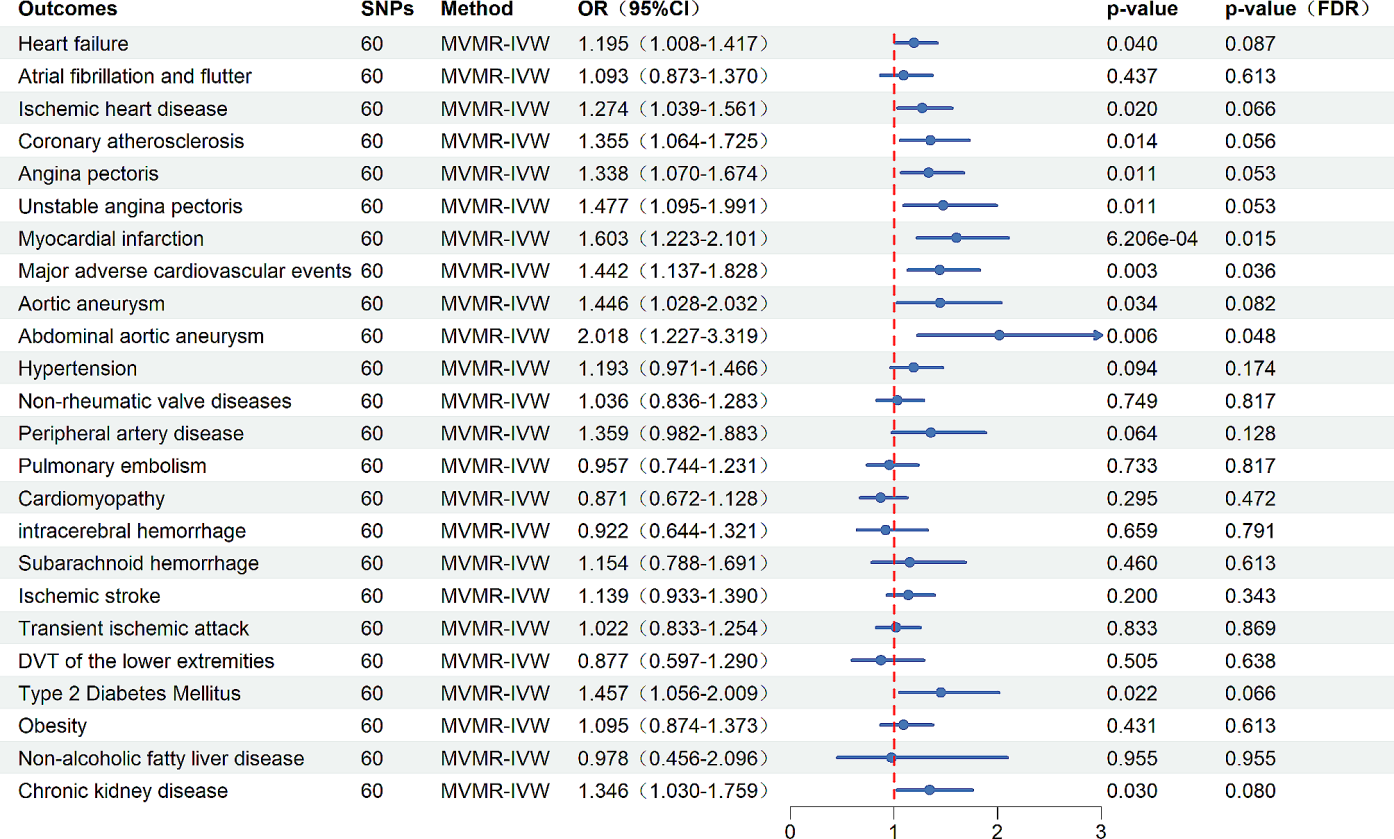
Causal effect of the ApoB/ApoA1 ratio on CMD from multivariable MR analysis. (Abbreviation: OR, Odds ratio; CI, Confidence interval; FDR, False discovery rate; DVT, Deep vein thrombosis)

In the multivariate MR analysis, adjusting for triglycerides (TG) and remnant cholesterol, the ApoB/ApoA1 ratio maintained a significant correlation with the occurrence of MI, AAA and the incidence of MACE (all *P*_FDR_<0.05). Furthermore, the ApoB/ApoA1 ratio exhibited a nominally significant association with heart failure, IHD, coronary atherosclerosis, angina pectoris, unstable angina pectoris and aortic aneurysm (all *P* < 0.05, *P*_FDR_>0.05). Importantly, in the multivariate MR analysis, the ApoB/ApoA1 ratio demonstrated a nominally significant association with metabolic disorders, including T2DM and CKD (all *P* < 0.05, *P*_FDR_>0.05). However, in the MR analysis adjusted for variables, the association between the ApoB/ApoA1 ratio and PAD, cerebrovascular diseases (including TIA and ischemic stroke), non-rheumatic valve diseases, atrial fibrillation and atrial flutter diseases were significantly weakened (all *P*>0.05) (Fig. 5, Additional file, Table S7).

### Mediation MR of the ApoB/ApoA1 ratio, CMD and relative risk factors

**Fig. 6:**
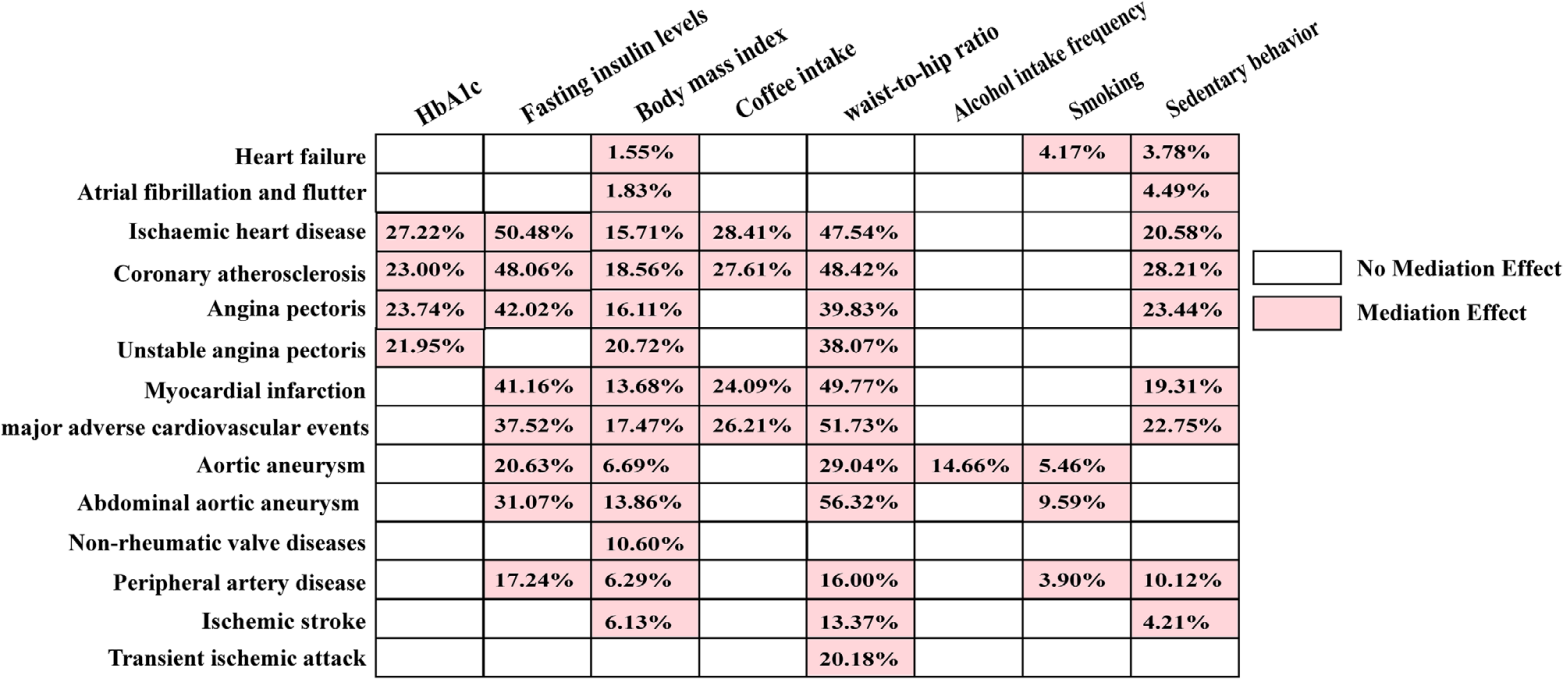
The mediation effect of the ApoB/ApoA1 ratio in CMD and its risk factors. (Abbreviation: HbA1c, hemoglobin A1c)

In mediation analysis, we first assessed the impact of 18 CMD risk factors on the ApoB/ApoA1 ratio. After FDR correction, we observed a significant correlation between 11 CMD risk factors and the ApoB/ApoA1 ratio. Subsequently, we separately evaluated these 11 CMD risk factors with 24 different types of CMD to obtain CMD types that were significantly associated with specific risk factors. We conducted FDR correction and ultimately assessed the mediating effect of the ApoB/ApoA1 ratio in CMD and its risk factors. The findings revealed a significant mediating effect of the ApoB/ApoA1 ratio on 8 risk factors associated with CMD. These factors include HbA1c, fasting insulin levels, BMI, waist-to-hip ratio, coffee intake, alcohol intake frequency, smoking, and sedentary behavior. Additionally, the ApoB/ApoA1 ratio exhibited a mediating effect on 14 different types of CMD. Specific mediation proportions can be found in Fig. 6 (Additional file, Tables S8-9). Furthermore, since HDL-c and LDL-c were key components of the ApoB/ApoA1 ratio, removing either of them would significantly alter the ApoB/ApoA1 ratio. Therefore, it is not suitable to calculate the mediation proportions in multivariate MR analysis.

## Discussion

**Fig. 7:**
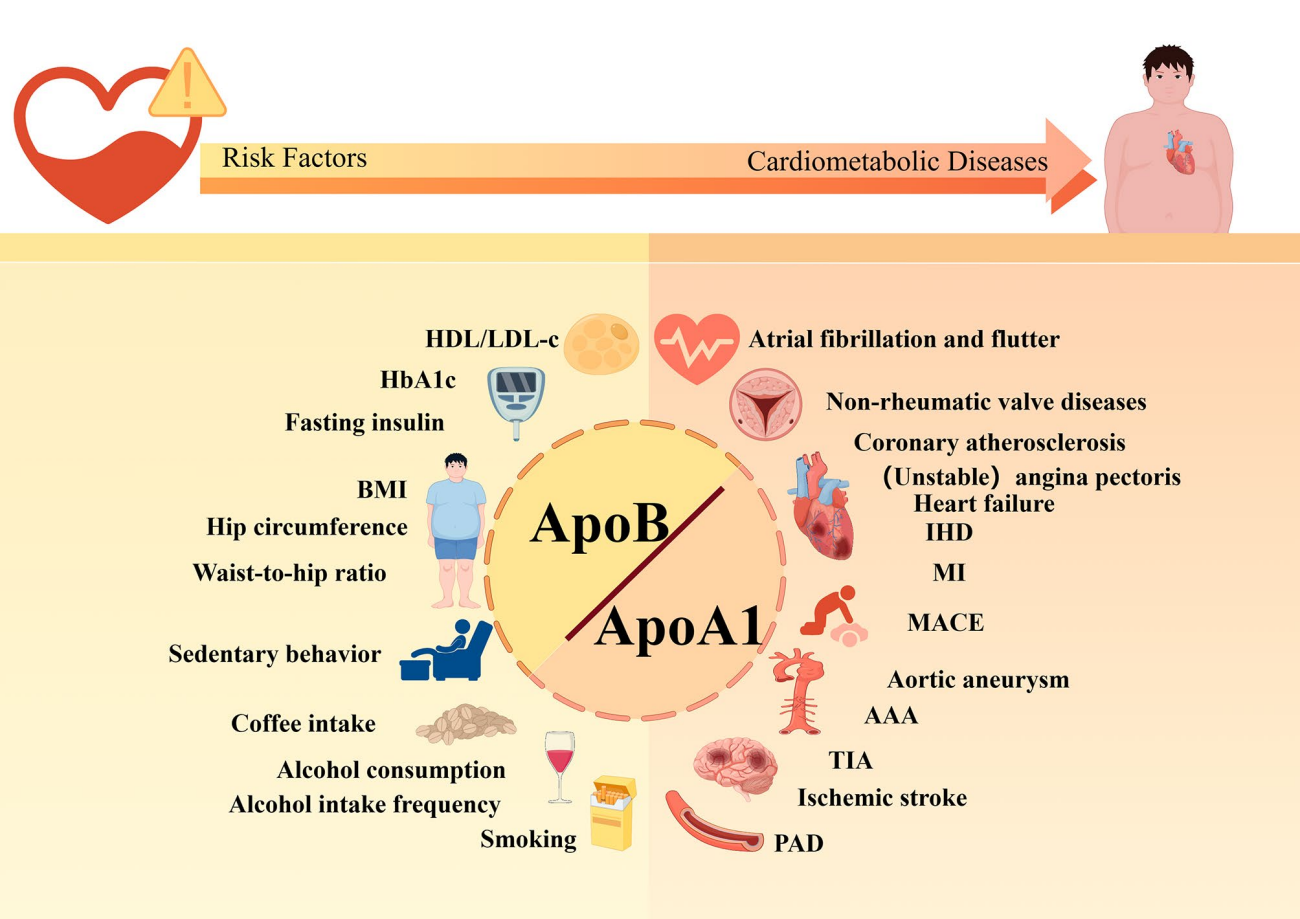
The genetic prediction of the ApoB/ApoA1 ratio with a causal relationship to CMD and risk factors. (Abbreviation: HDL-c, High-density lipoprotein cholesterol; LDL-c, Low-density lipoprotein cholesterol; HbA1c, hemoglobin A1c; BMI, Body mass index; IHD, Ischemic heart disease; MI, Myocardial infarction; MACE, Major adverse cardiovascular events; AAA, Abdominal aortic aneurysm; TIA, Transient ischemic attack; PAD, Peripheral artery disease; ApoB, apolipoprotein B; ApoA1, Apolipoprotein A1)

In this study, MR was employed to elucidate the causal association between the ApoB/ApoA1 ratio and CMD alongside its associated risk factors. The findings provide confirmation of the mediating influence of the ApoB/ApoA1 ratio on both CMD and its associated risk factors. Our study found the following conclusions: (1) In terms of CMD, the increase in the ApoB/ApoA1 ratio was significantly related to ischemic diseases such as heart disease, cerebrovascular disease, and PAD. An increased ratio also led to an increase in MACE. In addition, an elevated ApoB/ApoA1 ratio also increased the incidence of aortic aneurysm (especially AAA), non-rheumatic valve diseases, and atrial flutter and fibrillation. After performing multivariable MR analysis, it was found that the increased ApoB/ApoA1 ratio still had a significant causal relationship with MI, AAA, and an increase in MACE. (2) Regarding CMD risk factors, a significant bidirectional causality existed between the ApoB/ApoA1 ratio and HDL-c, LDL-c. Meanwhile, the ApoB/ApoA1 ratio also had a strong causality with glucose metabolism indicators (like HbA1c, fasting insulin levels), obesity-related indicators (like BMI, waist-to-hip ratio), and behaviorally relevant indicators (such as smoking, sedentary behavior, (frequent) alcohol consumption, and coffee intake) (specific results are shown in Fig. 7).

Compared to traditional LDL-c, the ApoB/ApoA1 ratio has significant implications in predicting coronary atherosclerosis disease. Many observational studies have also validated its application value in cardiovascular diseases. Lu et al. have found that the ApoB/ApoA1 ratio was an important predictor of coronary disease in overweight and obese patients [32]. Moreover, high ApoB/ApoA1 levels were associated with subclinical atherosclerosis and unstable plaque disease [33]. Bodde et al. found that the ApoB/ApoA1 ratio was associated with the first occurrence of ST-segment elevation myocardial infarction (STEMI), but cannot predict its incidence of MACE during follow-up [25]. Recent observational studies showed that the ApoB/ApoA1 ratio can predict the progression of non-major coronary artery lesions and the occurrence of MACE in patients with diabetes complicated with acute coronary syndrome after percutaneous coronary intervention [24]. Despite many studies on the ApoB/ApoA1 ratio usually being limited to observational research, and potential confounding factors that may affect the accuracy of the results. However, considering the potential value of ApoB/ApoA1 ratio in predicting IHD, our study analyzed its common representative diseases, such as coronary atherosclerosis, angina pectoris and ACS (including unstable angina pectoris and MI). The results showed that the rise in the ApoB/ApoA1 ratio significantly increased the incidence of these related diseases. Previous studies on the relationship between the ApoB/ApoA1 ratio and the incidence of MACE have been different. Our study showed that as the ApoB/ApoA1 ratio increased, the incidence of MACE also increased. The research results of Huang and others were consistent with our findings. They found that the sensitivity of predicting MACE in the 3-month follow-up period with the ApoB/ApoA1 ratio was 84%, and the specificity of predicting the number of atherosclerosis vessels in the 1-year follow-up period was 81% [34].

In cerebrovascular ischemic diseases, the ApoB/ApoA1 ratio still has a significant predictive role. Sabino et al. found that an increase in the ApoB/ApoA1 ratio was independently associated with the occurrence of ischemic stroke in young patients, which was consistent with our research results [35]. TIA is often referred to as a “small stroke” and is considered a precursor to ischemic stroke. However, it is unclear whether the ApoB/ApoA1 ratio has predictive value for it, so we conducted relevant verification. The results showed that as the ApoB/ApoA1 ratio increased, the incidence of TIA also increased. In addition, the incidence of PAD was also significantly correlated with the ApoB/ApoA1 ratio. These studies suggested that in addition to its predictive role in IHD, the ApoB/ApoA1 ratio also had important value in cerebrovascular disease and PAD. On the other hand, atherosclerosis is one of the important causes of aortic aneurysm formation [36, 37]. Our research has found that an increase in the ApoB/ApoA1 ratio was significantly related to the formation of aortic aneurysm. Hernesniemi’s meta-analysis reported a positive association between coronary heart disease and the occurrence of subclinical AAA, and indicated that coronary heart disease was a strong predictor of future AAA events [38]. AAA and coronary heart disease are considered different manifestations of atherosclerosis. On this basis, Xiao et al. found that the ApoB/ApoA1 ratio had some value in AAA prediction [39], which was consistent with our research results. Unlike cerebrovascular ischemic diseases, our research did not find a causal relationship between the ApoB/ApoA1 ratio and cerebrovascular hemorrhagic diseases such as intracerebral hemorrhage and subarachnoid hemorrhage. However, Rasha et al. found that an increase in the ApoB/ApoA1 ratio at admission was independently associated with poor functional prognosis and all-cause mortality at one year follow-up in patients with intracerebral hemorrhage [40]. This suggested that the ApoB/ApoA1 ratio had some indicative meaning in the prognosis of patients with cerebral hemorrhage.

Obesity is one of the common diseases of CMD, which can be divided into central obesity and peripheral obesity according to different fat distributions [41, 42]. Central obesity is an important risk factor for CMD, with waist circumference, hip circumference and waist-to-hip ratio as important measurement standards [43]. A prospective study found that these central obesity-related indicators could be combined with BMI to predict mortality risk [44]. Lee et al. found that in the adolescent metabolic syndrome population, the ApoB/ApoA1 ratio was significantly associated with BMI, waist circumference, waist-to-hip ratio and abdominal fat area, suggesting its predictive value in adolescent metabolic syndrome [45]. Similarly, in our study, we found a significant causal relationship between the ApoB/ApoA1 ratio and BMI, hip circumference and waist-to-hip ratio. An interesting observation is that, in univariate MR analysis, an increase in obesity-related indicators significantly elevates the ApoB/ApoA1 ratio. However, after reverse MR analysis, the elevation of the ApoB/ApoA1 ratio seems to be correlated with the decrease in obesity-related indicators. This phenomenon may be attributed to lipid transformation, as an increase in the ApoB/ApoA1 ratio implies an increase in free lipids, which primarily originate from the breakdown of subcutaneous adipose tissue. Consequently, this may lead to a decrease in indicators associated with obesity [46]. Furthermore, in an MR study on residual cholesterol and CMD risk factors, similar results were obtained [47].

In terms of biological behavior characteristics, coffee intake, smoking, and sedentary behavior have a positive causality with ApoB/ApoA1 ratio. However, alcohol consumption was negatively correlated with the ApoB/ApoA1 ratio, but frequent alcohol consumption can increase the ApoB/ApoA1 ratio. Previous MR studies have found that low-to-moderate alcohol consumption was associated with increased levels of HDL-c [48]. In alcohol consumption database, the mean alcohol intake was 15.13 units per week, which falls within this range, so the effect of alcohol consumption on the ApoB/ApoA1 ratio may be caused by an increase in HDL-c levels [49]. Furthermore, through mediation MR analysis, it was not found that the decrease in the ApoB/ApoA1 ratio caused by alcohol consumption had a mediating effect on CMD and its risk factors. The relationship between alcohol consumption and cardiovascular diseases is complex, sometimes even contradictory, but excessive and frequent alcohol consumption is undoubtedly harmful [50]. In terms of glucose metabolism disorders, blood glucose-related indicators (HbA1c, fasting insulin levels) play an important role. Observational studies found that the ApoB/ApoA1 ratio was significantly associated with insulin resistance in non-diabetic subjects and could become an independent predictor of insulin resistance [51]. However, due to these findings being derived from observational studies, the causal relationship remains unclear. Notably, our MR study found a significant positive association between fasting insulin level and ApoB/ApoA1 ratio. Diaf et al. found that compared to the traditional fasting and postprandial lipid ratios, BMI, waist circumference and HbA1c levels had a greater impact on the ApoB/ApoA1 ratio in men and women, and our research results also support this finding [52]. However, we did not find a causal relationship between the ApoB/ApoA1 ratio and fasting glucose or sleep-related disorders (depression, insomnia, sleep apnea). This result is understandable. While current research has established association between sleep disorders and the incidence of CMD [53, 54], a significant amount of research indicates that sleep disorders can contribute to proinflammatory immune responses and endothelial dysfunction, pivotal factors in the pathogenesis of CMD [55, 56]. Our study indirectly indicated that the development of CMD due to sleep-related disorders was not associated with lipid abnormalities.

Our research provides several insights for clinical practice. First, the ApoB/ApoA1 ratio might be a more comprehensive lipid-related evaluation standard. Although many studies have proven the severe harm of residual cholesterol in CMD and its value in predicting CMD, this method has certain limitations, it has predictive efficacy only under the condition of well-controlled LDL-c levels. Second, in patients treated with statins and PCSK9 inhibitors, although the LDL-c level has reached the standard, the plasma TG has risen, which might or might not be accompanied by low levels of HDL-c, a common lipid abnormality in patients after treatment. In addition, traditional residual cholesterol evaluation criteria overlook the influence of HDL-c, which actually has a significant impact on CMD and ASCVD. Although current research has not yet proven that an increase in HDL-c levels can effectively protect ASCVD patients, epidemiological data have consistently linked a decrease in HDL-c to an increased risk of ASCVD [57, 58]. Third, in order to determine the influence and effectiveness of the ApoB/ApoA1 ratio in CMD and its risk factors, it is necessary to carry out large-scale randomized controlled trials. In clinical practice, we need to formulate appropriate lipid-lowering strategies based on the ApoB/ApoA1 ratio to reduce the occurrence of CMD and CMD risk factors as much as possible.

However, there were some limitations that need to be emphasized. Firstly, the source of GWAS statistical data was limited to individuals of European descent, therefore, our research findings may only be applicable to the European population and the applicability to other populations requires further verification. Secondly, there were a certain degree of heterogeneity and pleiotropy in our experiment, although we have used a variety of methods to verify the stability and reliability of the results. Thirdly, we did not consider HDL-c and LDL-c as confounding factors and include them in the multivariate Mendelian randomness analysis. The reason was that ApoB and ApoA1 mainly exist in LDL-c and HDL-c, including these two might seriously affect the accuracy of the results.

## Conclusion

In this study, we found from a genetic perspective that an increase in the ApoB/ApoA1 ratio is significantly associated with the occurrence of ischemic diseases, especially IHD, ischemic cerebrovascular disease, and PAD. In addition, there is a clear causal relationship between the ApoB/ApoA1 ratio and indicators related to glucose metabolism, obesity, and biological behavioral characteristics, serving as a mediating effect in CMD and related risk factor.

### Electronic supplementary material

Below is the link to the electronic supplementary material.

Supplementary Material 1

Supplementary Material 2

## Data Availability

The summary statistics utilized in this study are publicly accessible and downloadable from various websites. The genetic data concerning exposures and outcomes were sourced from the ninth data release of the FinnGen study (https://www.finngen.fi/en/access_results) and the UK Biobank database (https://www.ukbiobank.ac.uk/).
