# Supplementary material for: Revisiting an old relationship: the causal associations of the ApoB/ApoA1 ratio with cardiometabolic diseases and relative risk factors—a mendelian randomization analysis
Source: Cardiovasc Diabetol. 2024 Feb 3;23:51. doi: 10.1186/s12933-024-02140-2 (PMC10838437; doi:10.1186/s12933-024-02140-2)
Supplement: Supplementary file 1 — Supplementary Material 1 [file 12933_2024_2140_MOESM1_ESM.docx]

**Supplemental Online Content**

**Figure S1 Two-sample MR analyses: the casual effect of the ApoB/ApoA1 ratio on CMD**

**Figure S2 Two-sample MR analyses: the casual effect of CMD risk factors on the ApoB/ApoA1 ratio**

**Figure S3 Two-sample MR analyses: the casual effect of the ApoB/ApoA1 ratio on CMD risk factors**

**Figure S1. Two-sample MR analyses: the casual effect of the ApoB/ApoA1 ratio on CMD**

**(A) The singly SNP estimated the relationship between the ApoB/ApoA1 ratio and the incidence of CMD; (B) the Leave-one-out analysis in estimating the relationship between the ApoB/ApoA1 ratio and the incidence of CMD; (C) The five methods of MR test; (D) The scatter diagram for showing the tendency of dispersion in CMD.**

**Heart failure**

**
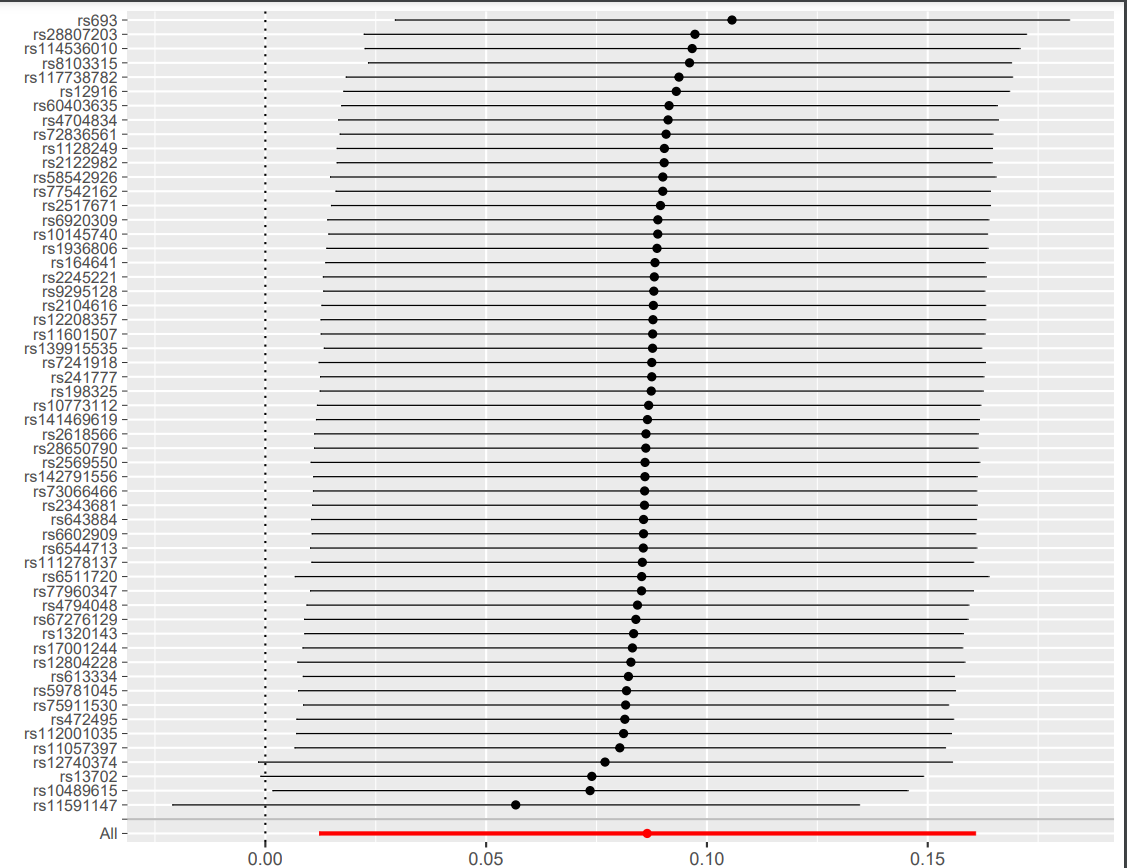

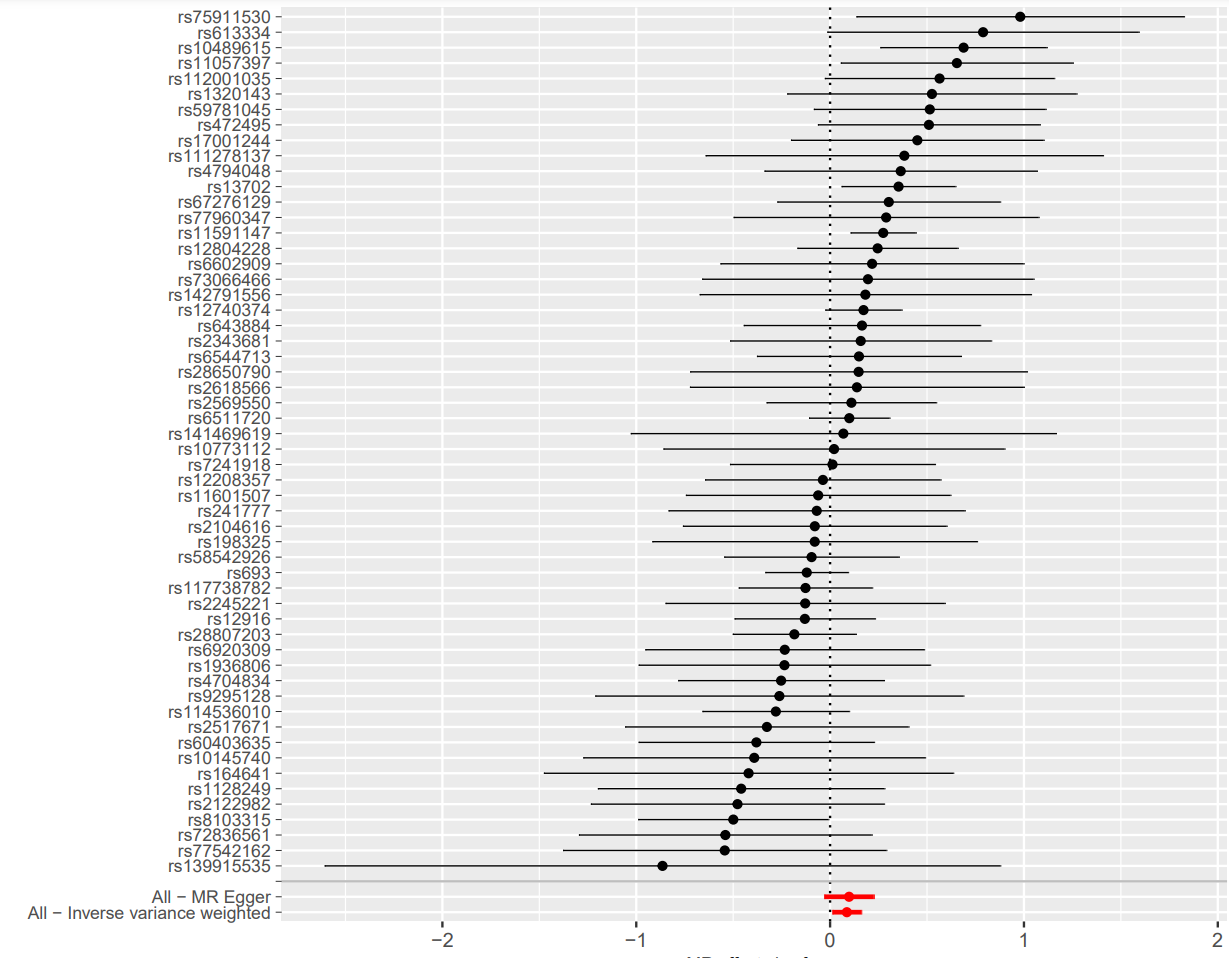
A B**

**
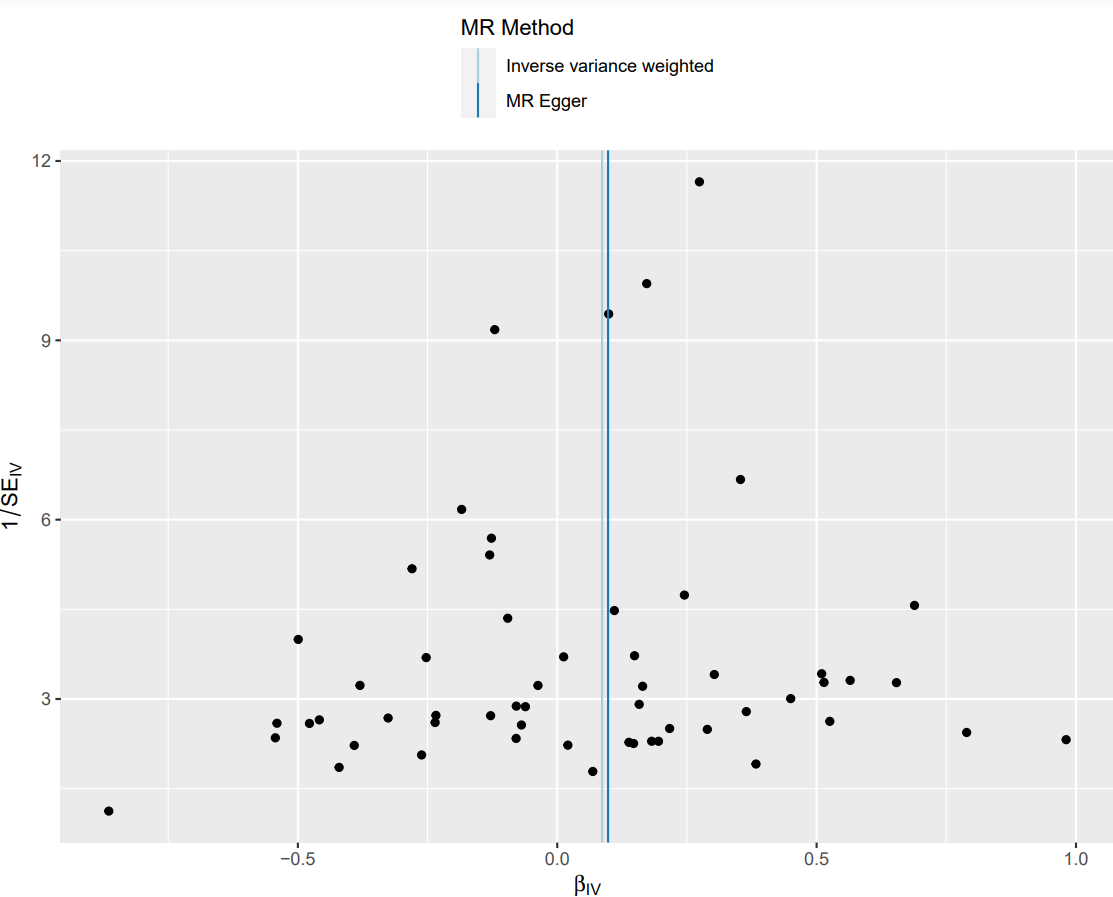

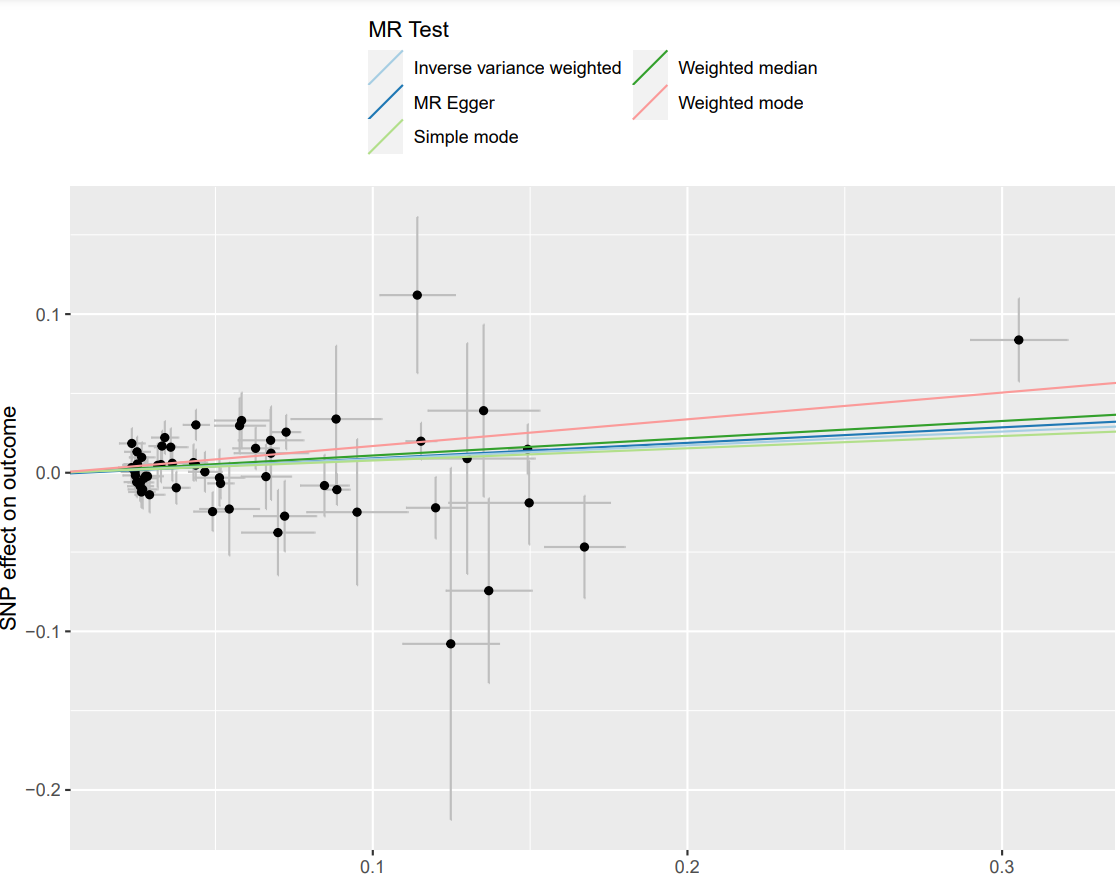
C D**

**Atrial fibrillation and flutter**

**
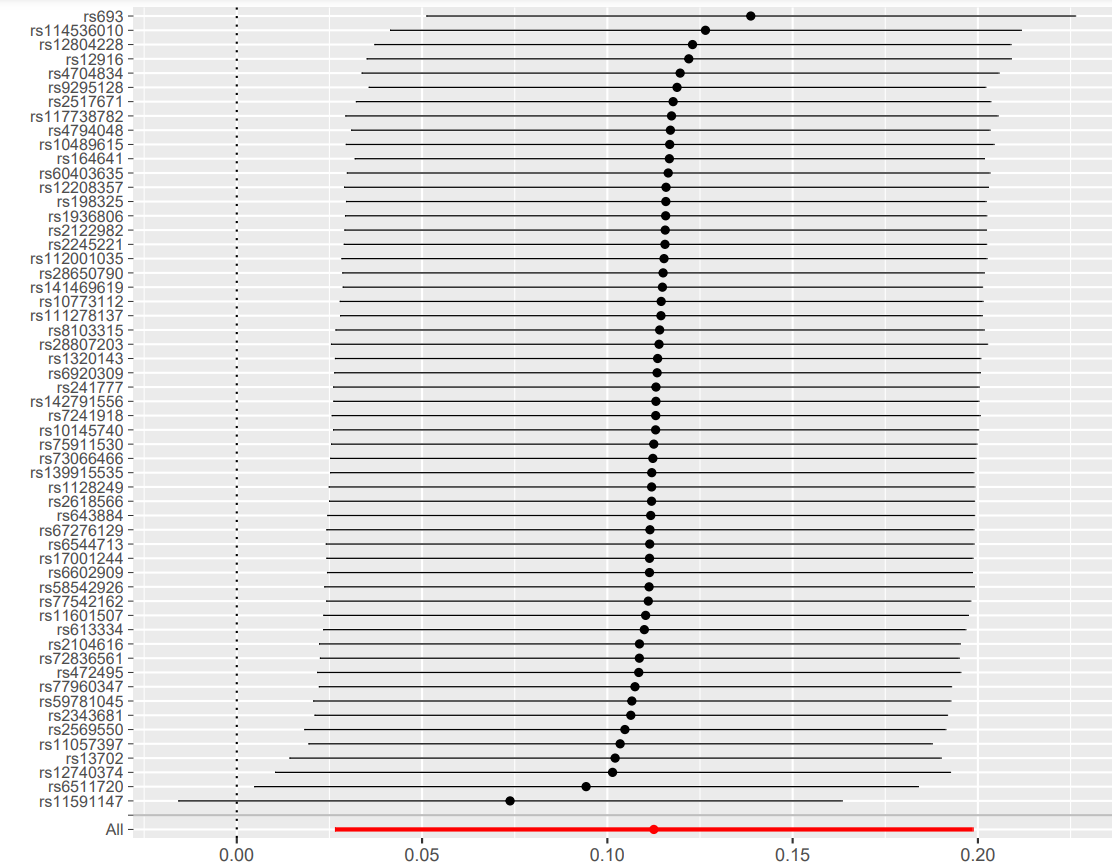

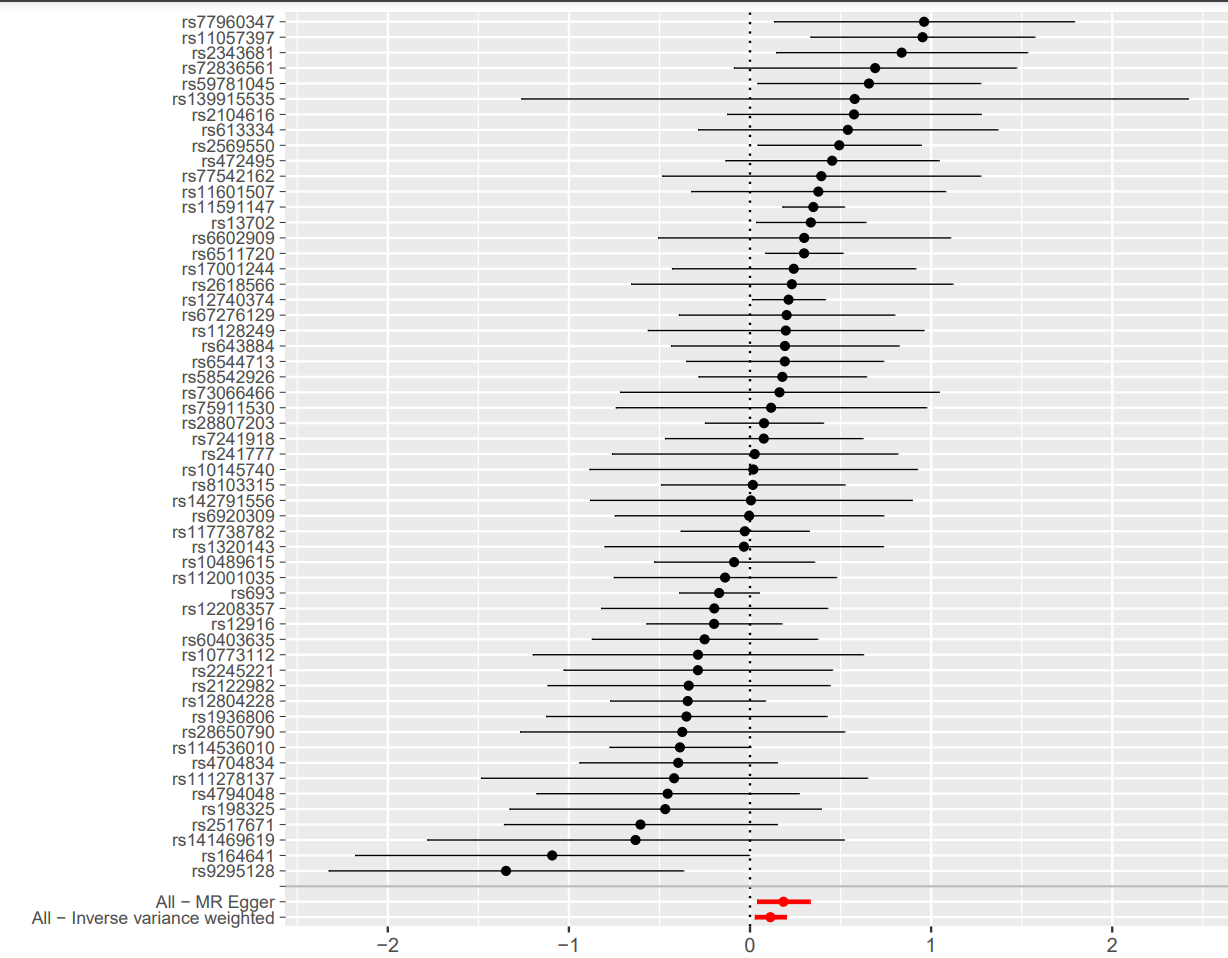
A B**

**
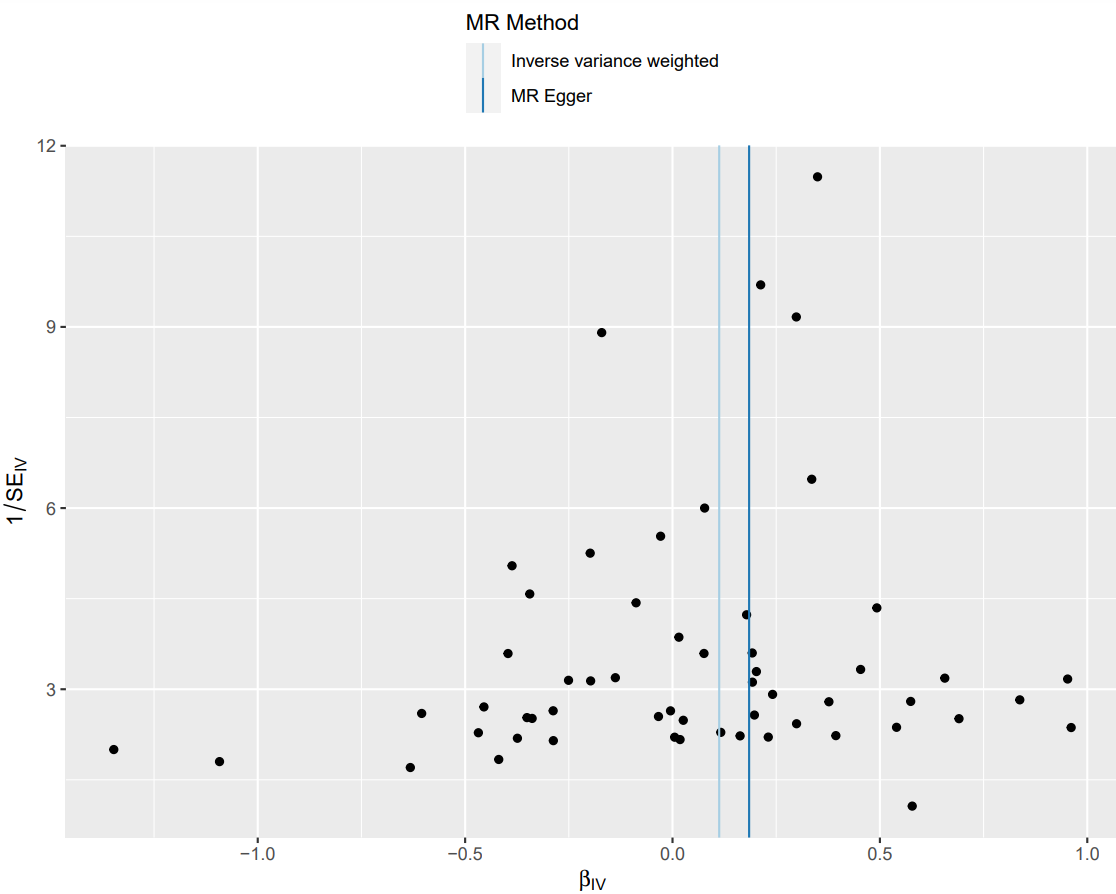

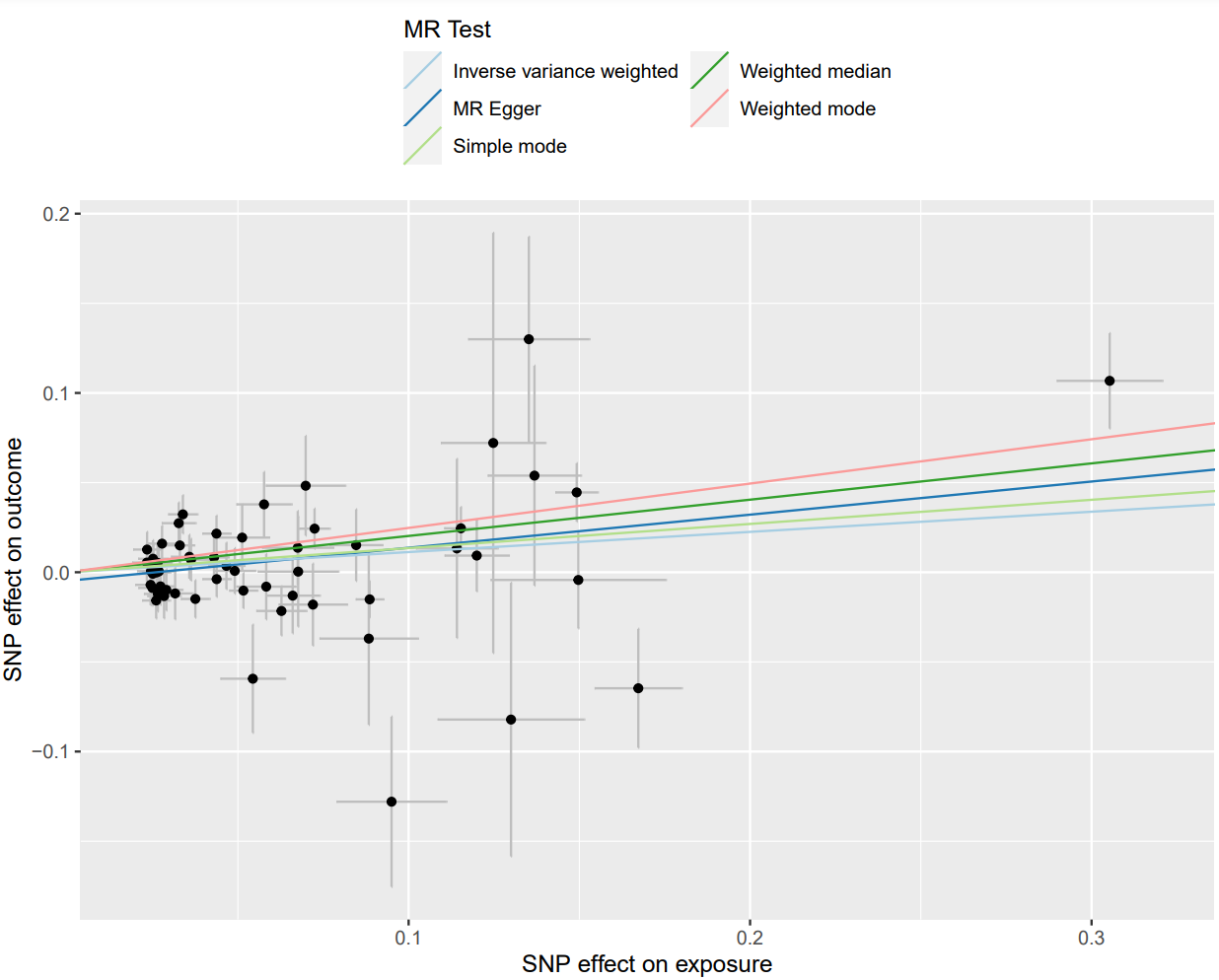
C D**

**Ischemic heart disease**

**
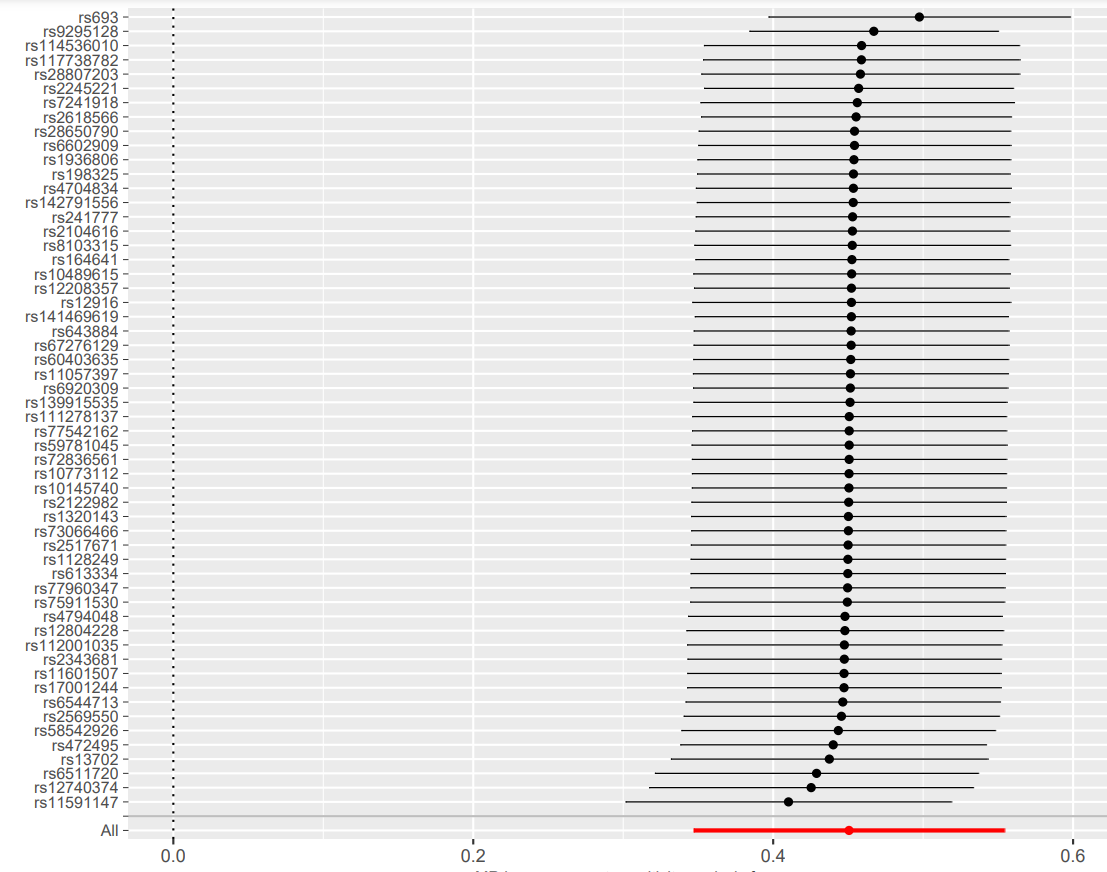

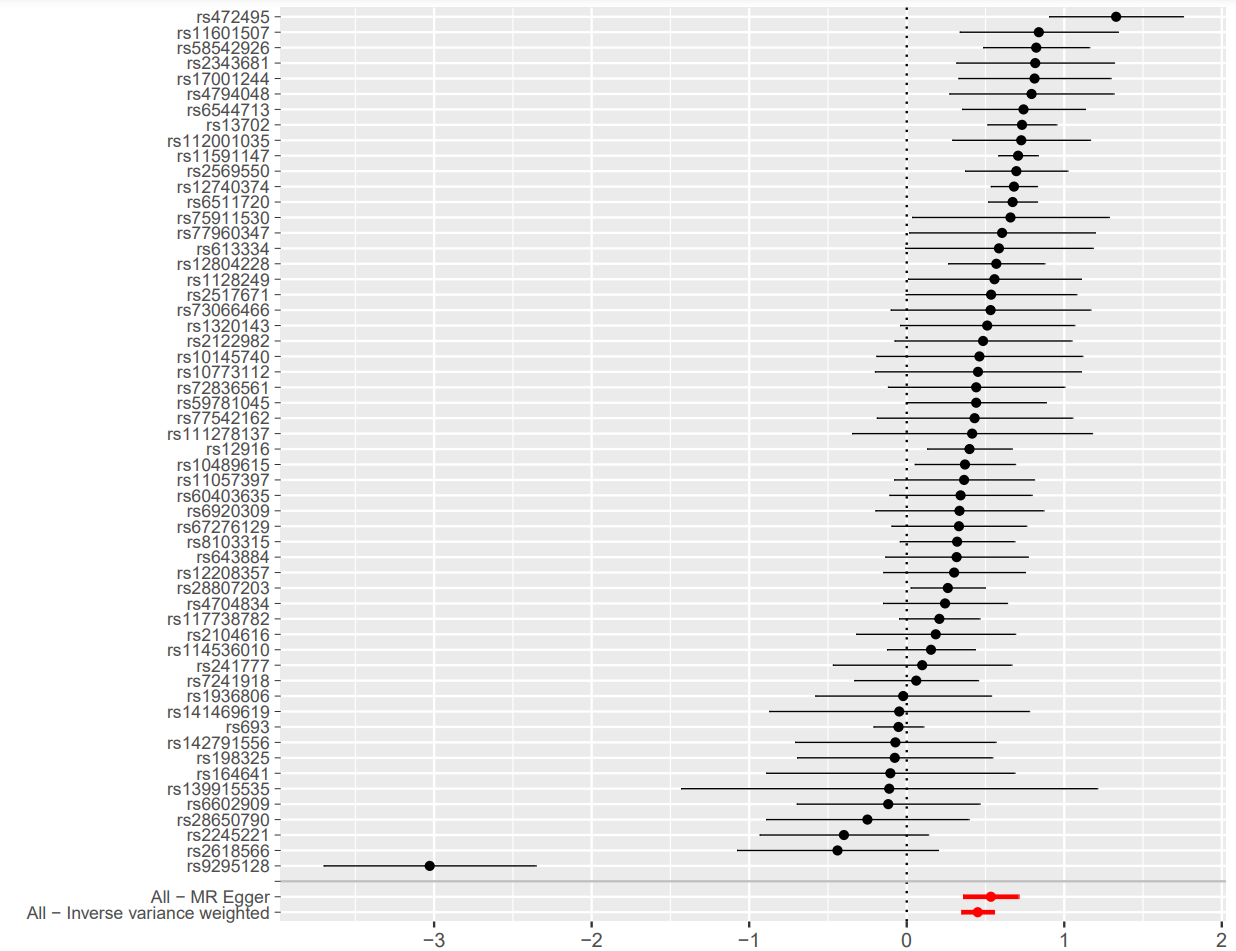
A B**

**C D**

**
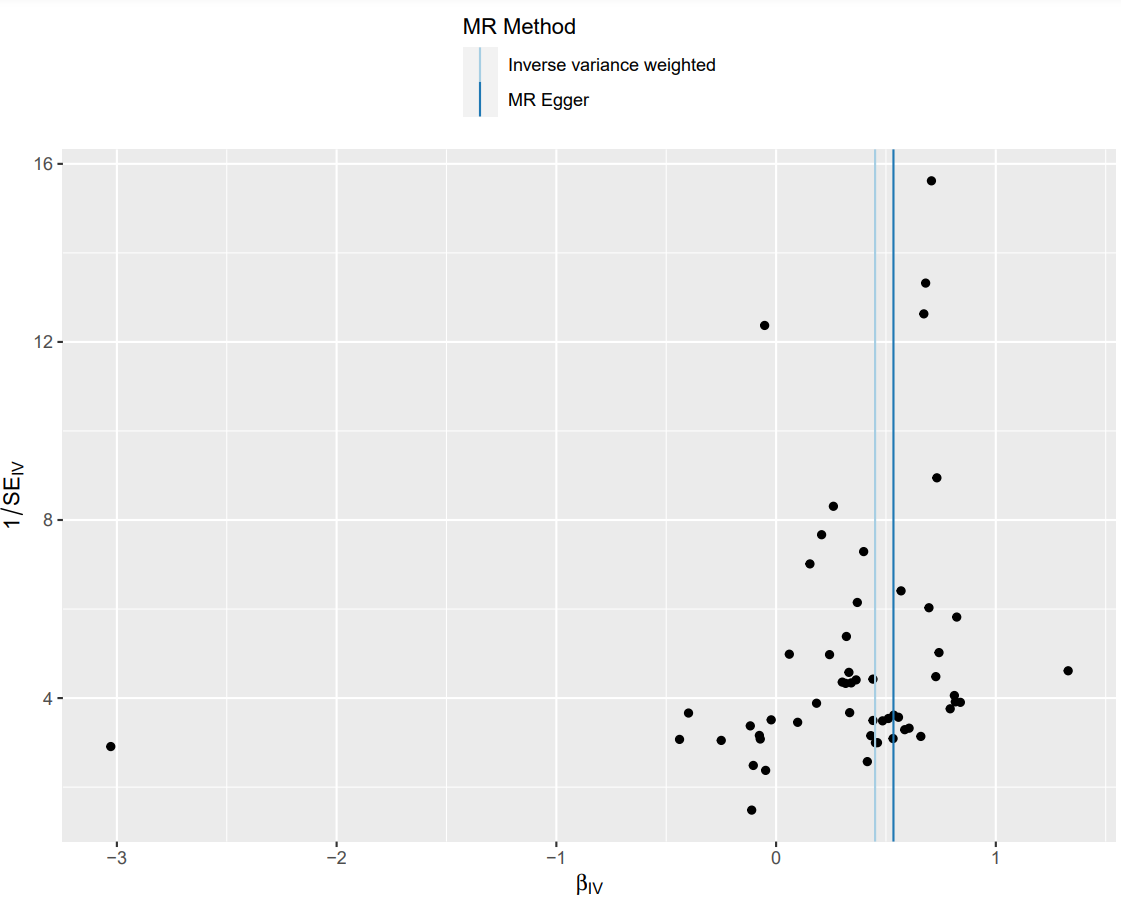

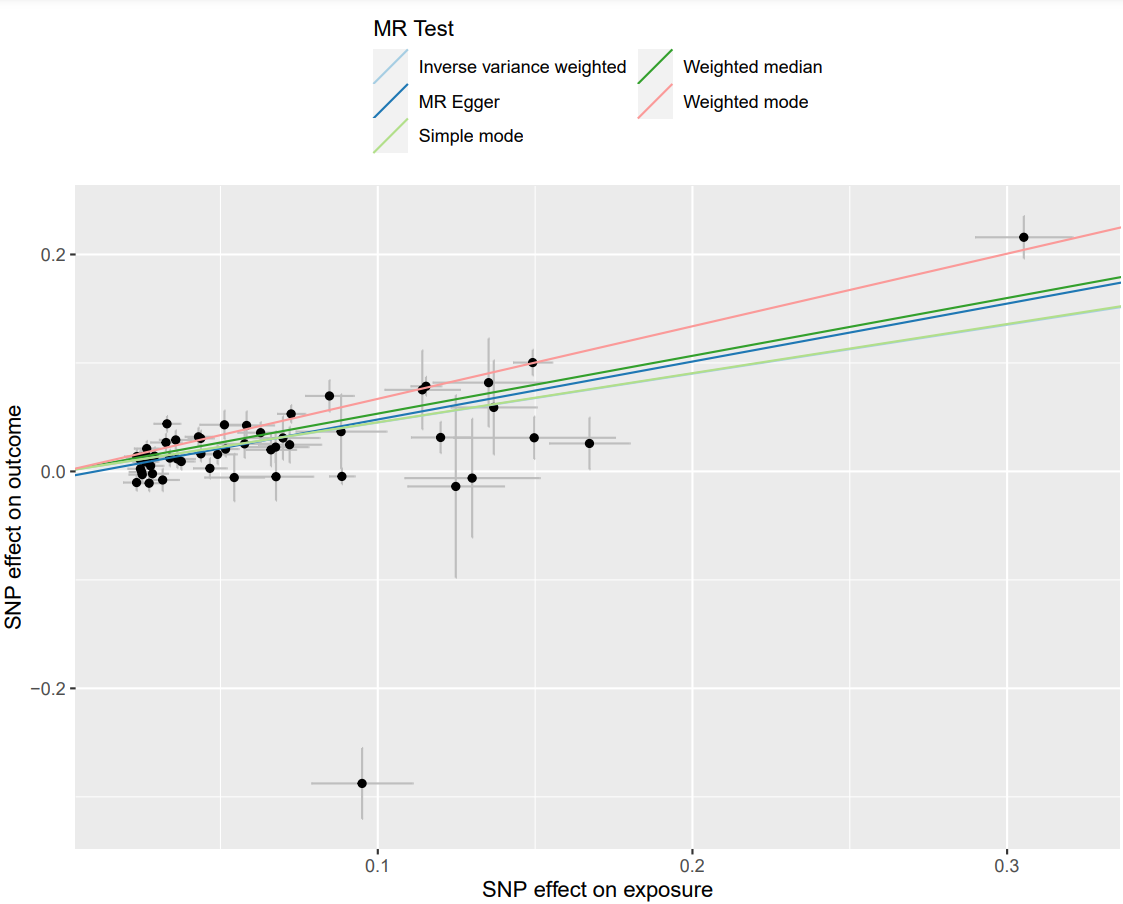
**

**Coronary atherosclerosis**

**
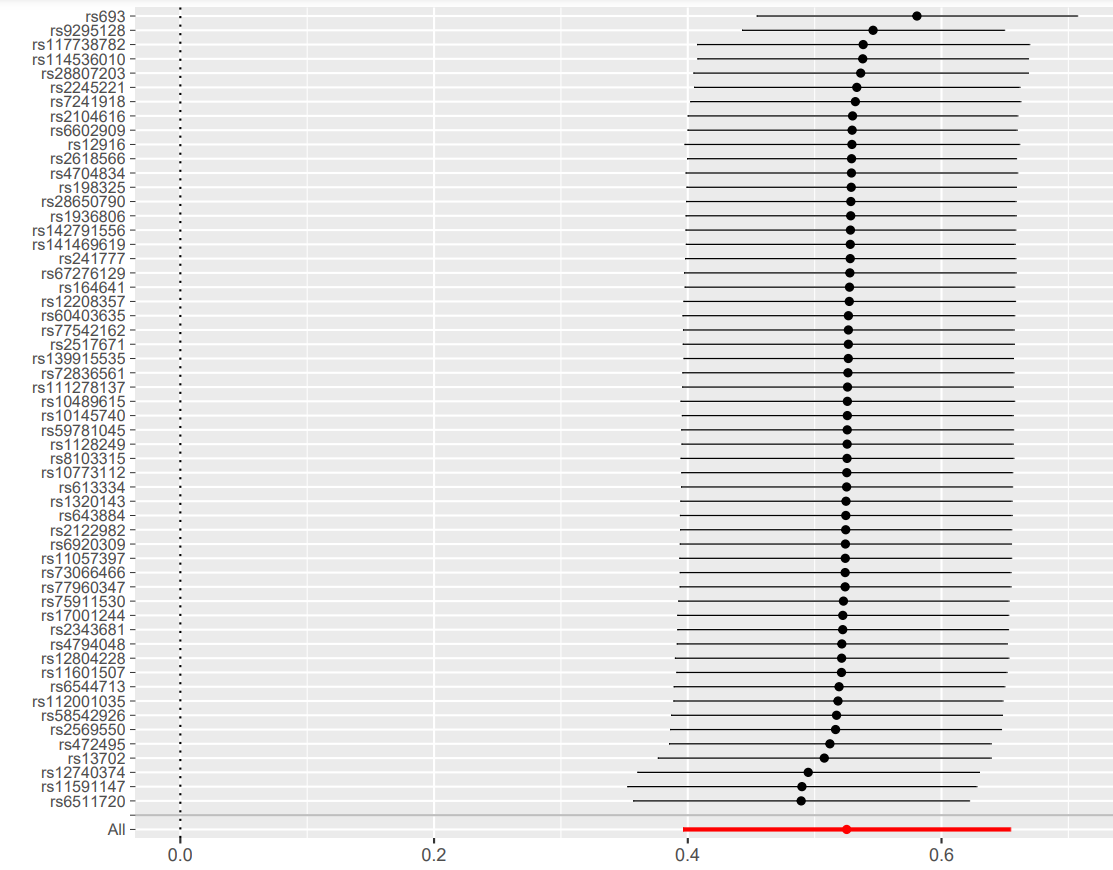

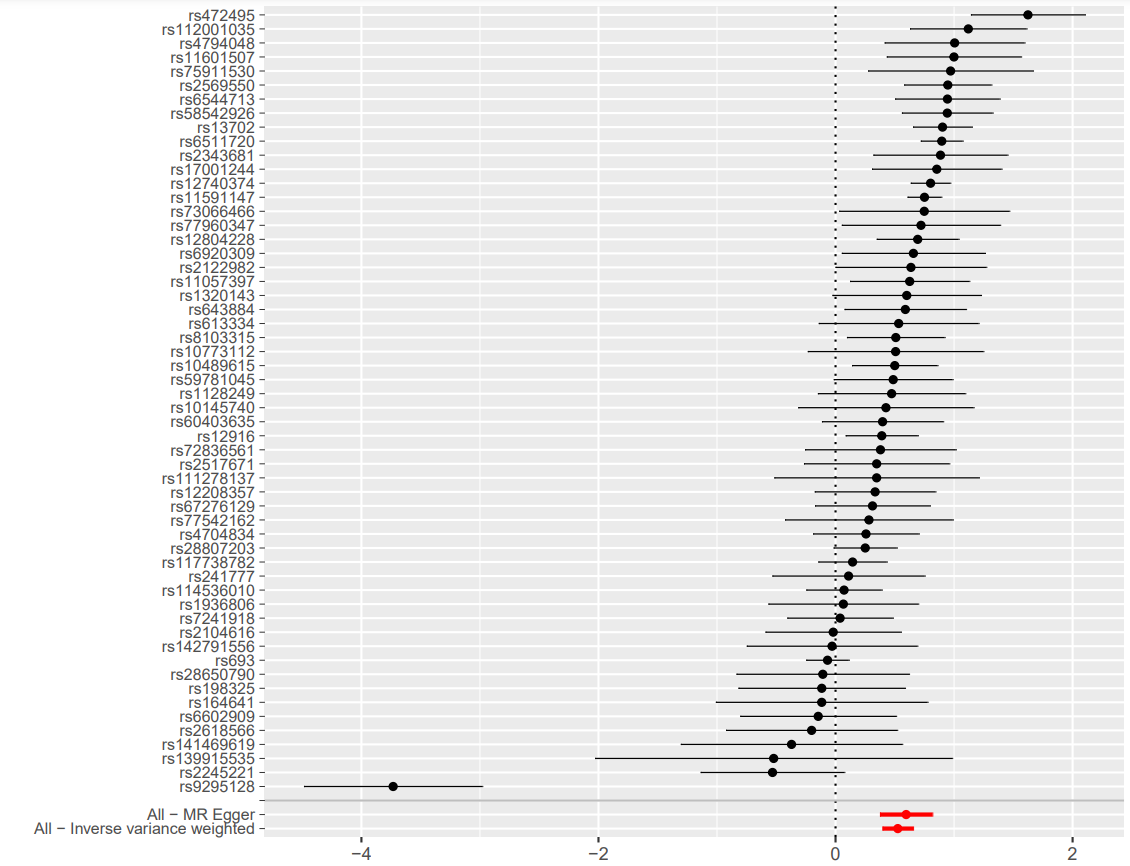
A B**

**
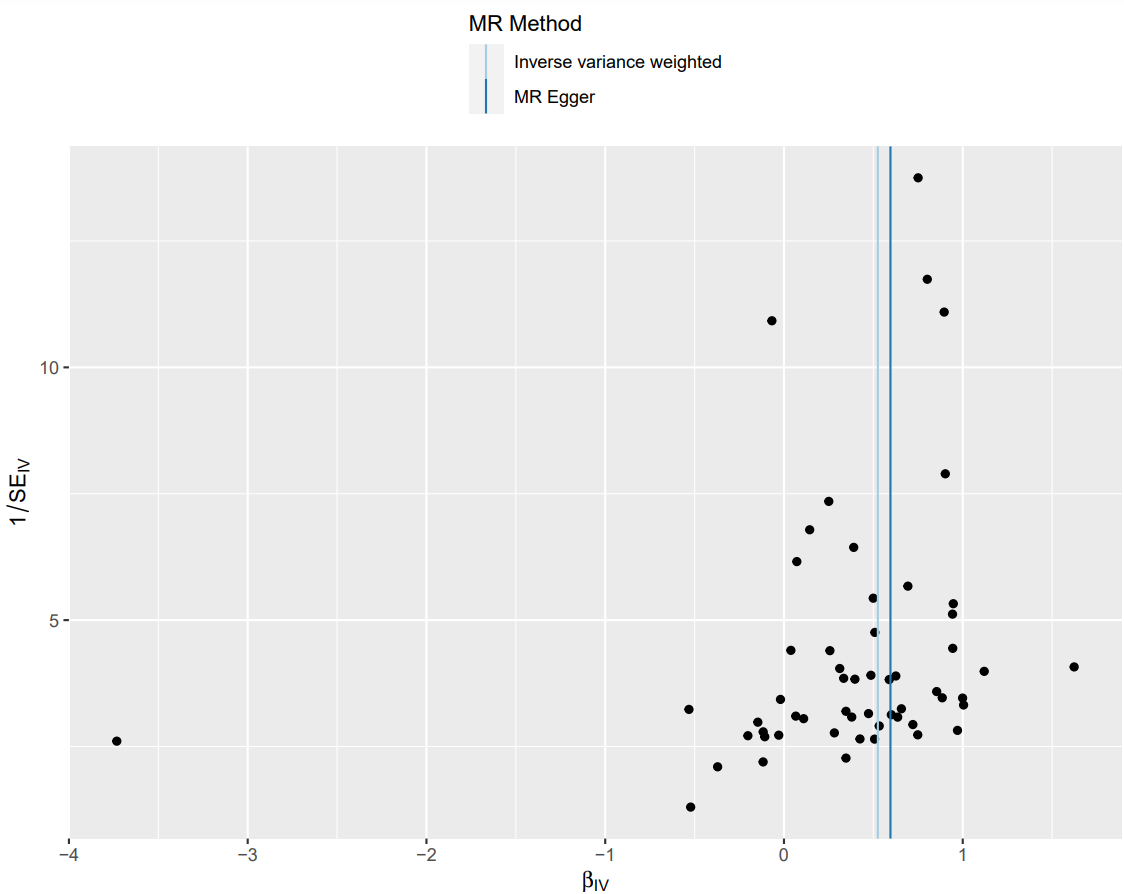

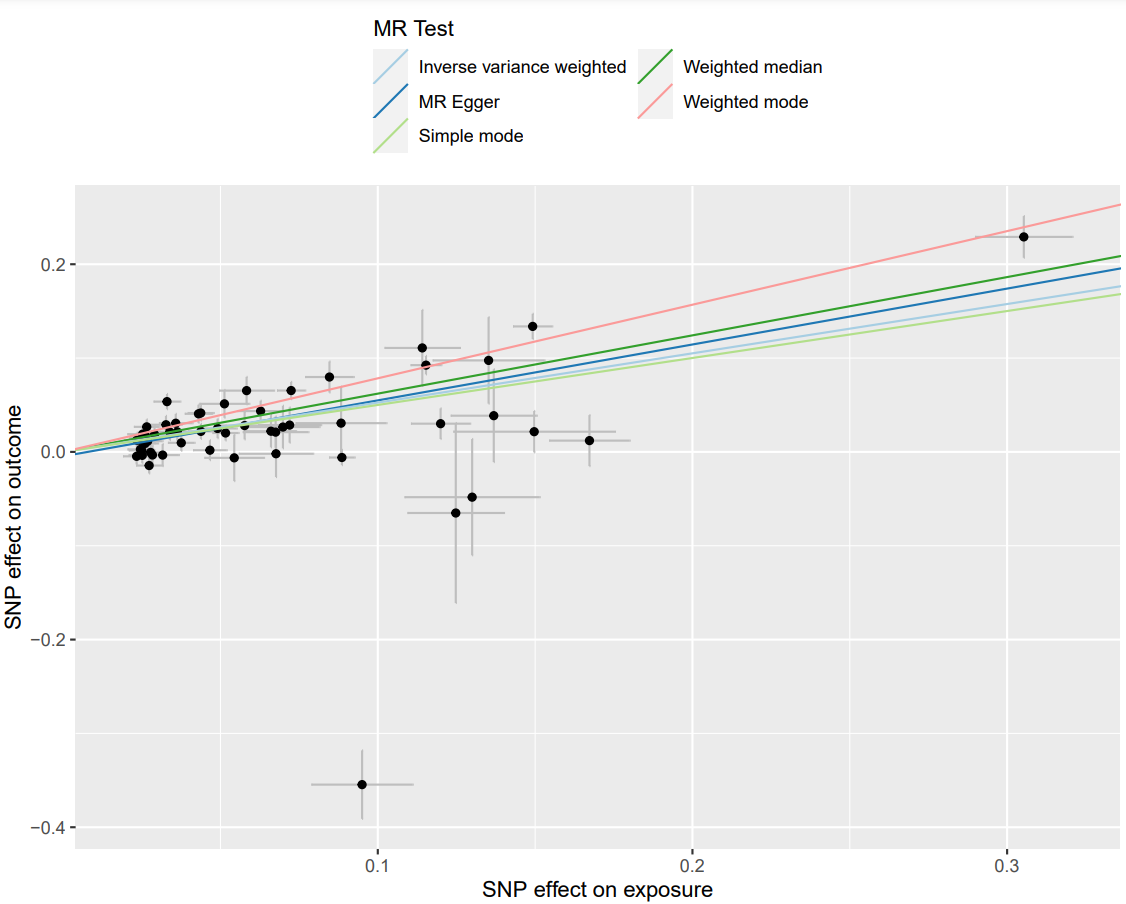
C D**

**Angina pectoris**

**
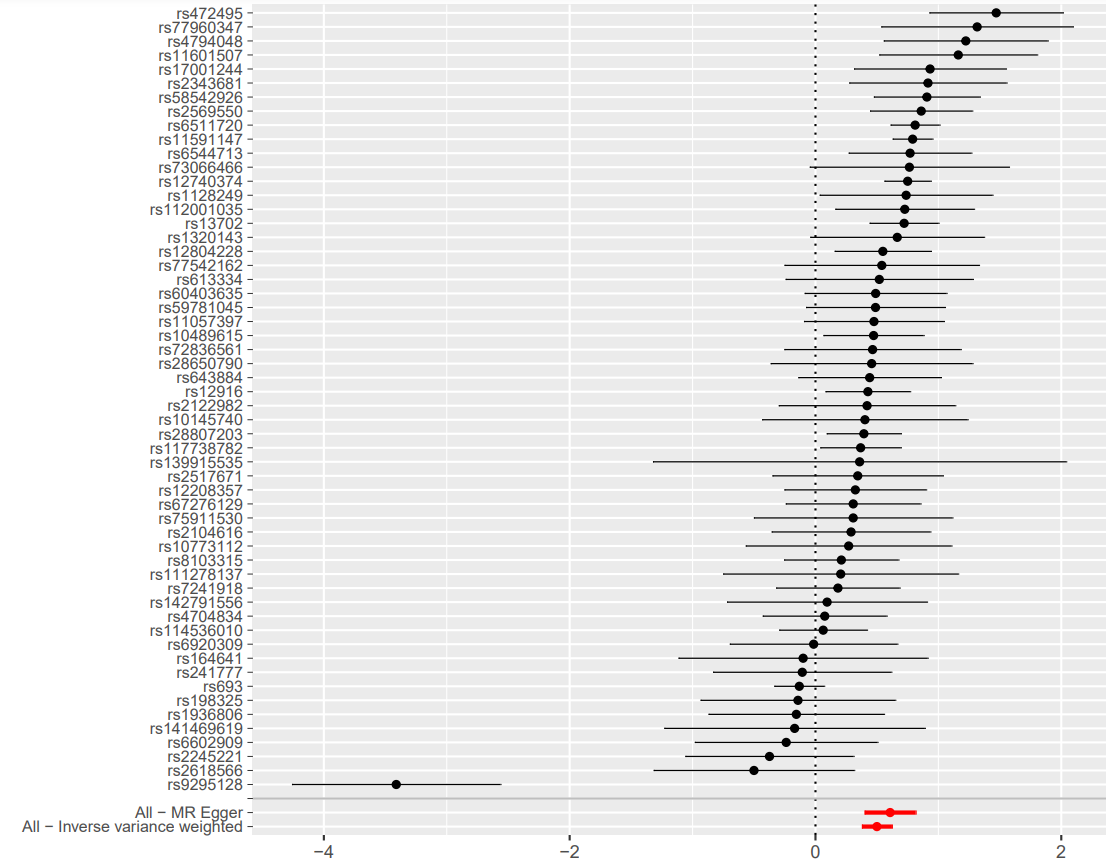

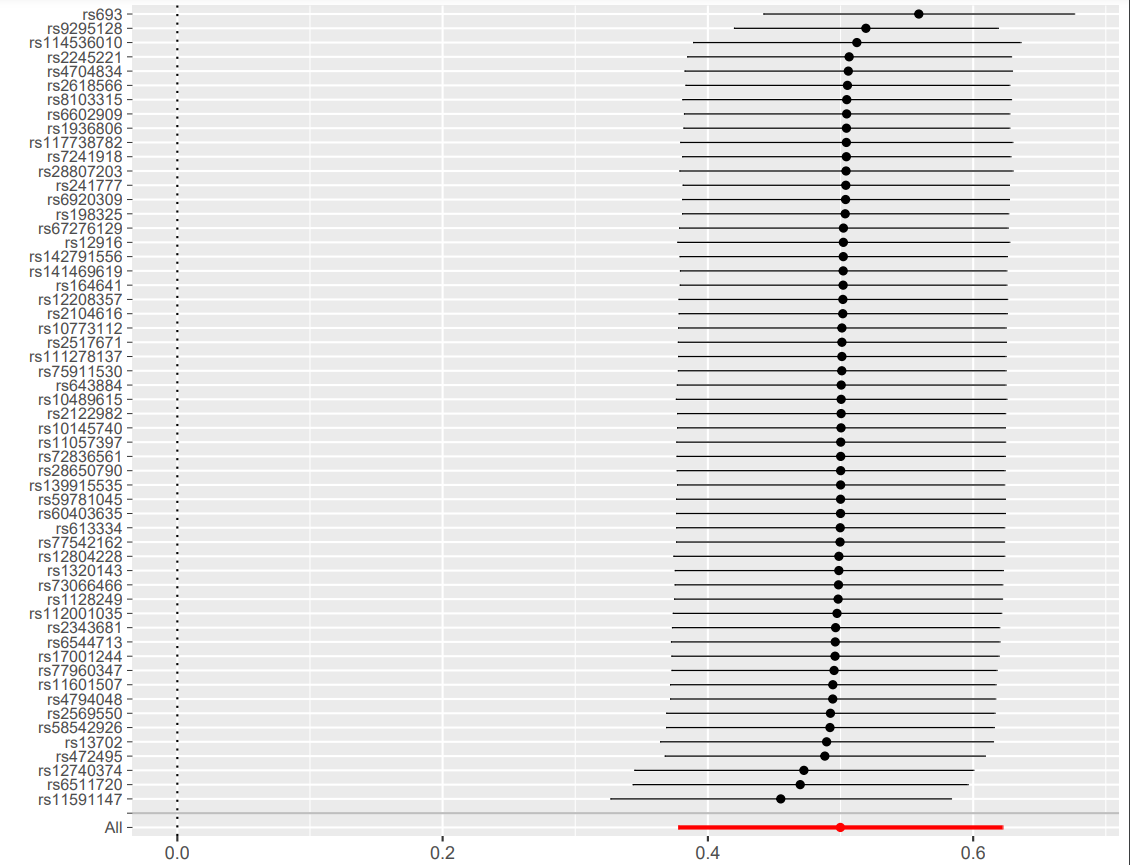
A B**

**
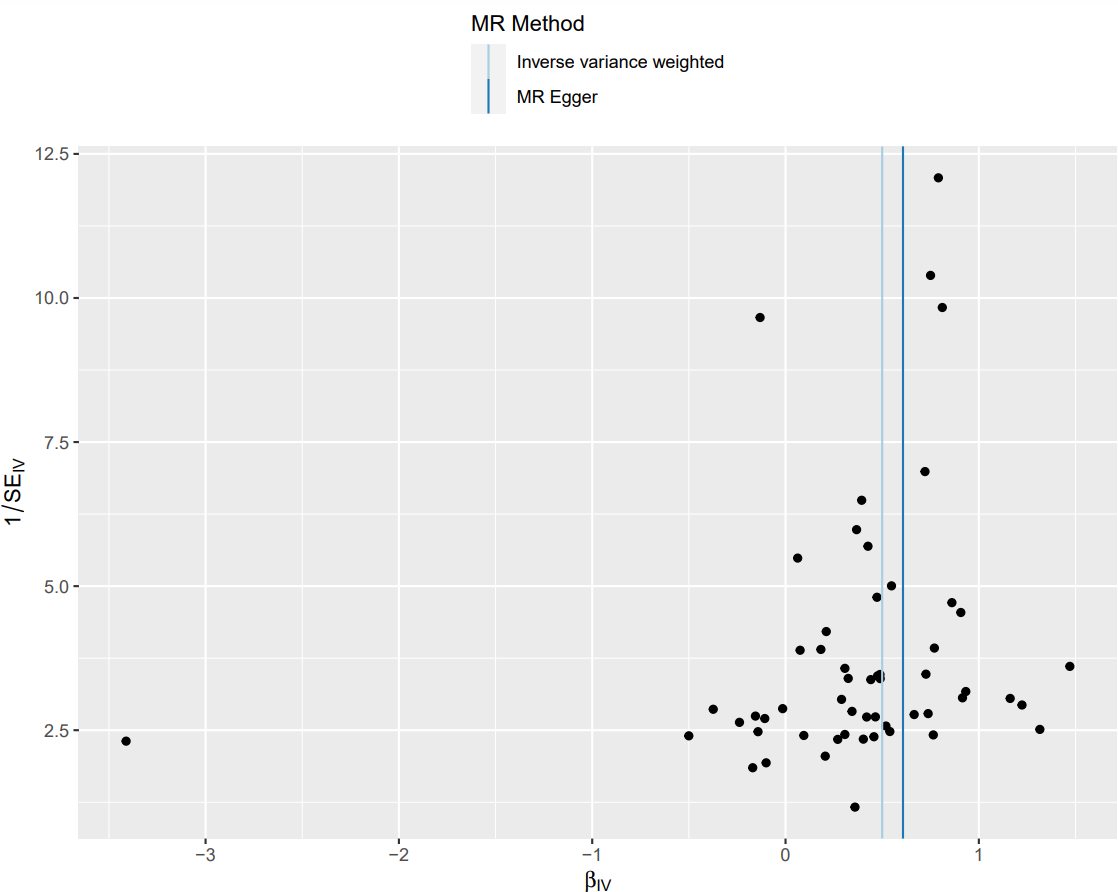

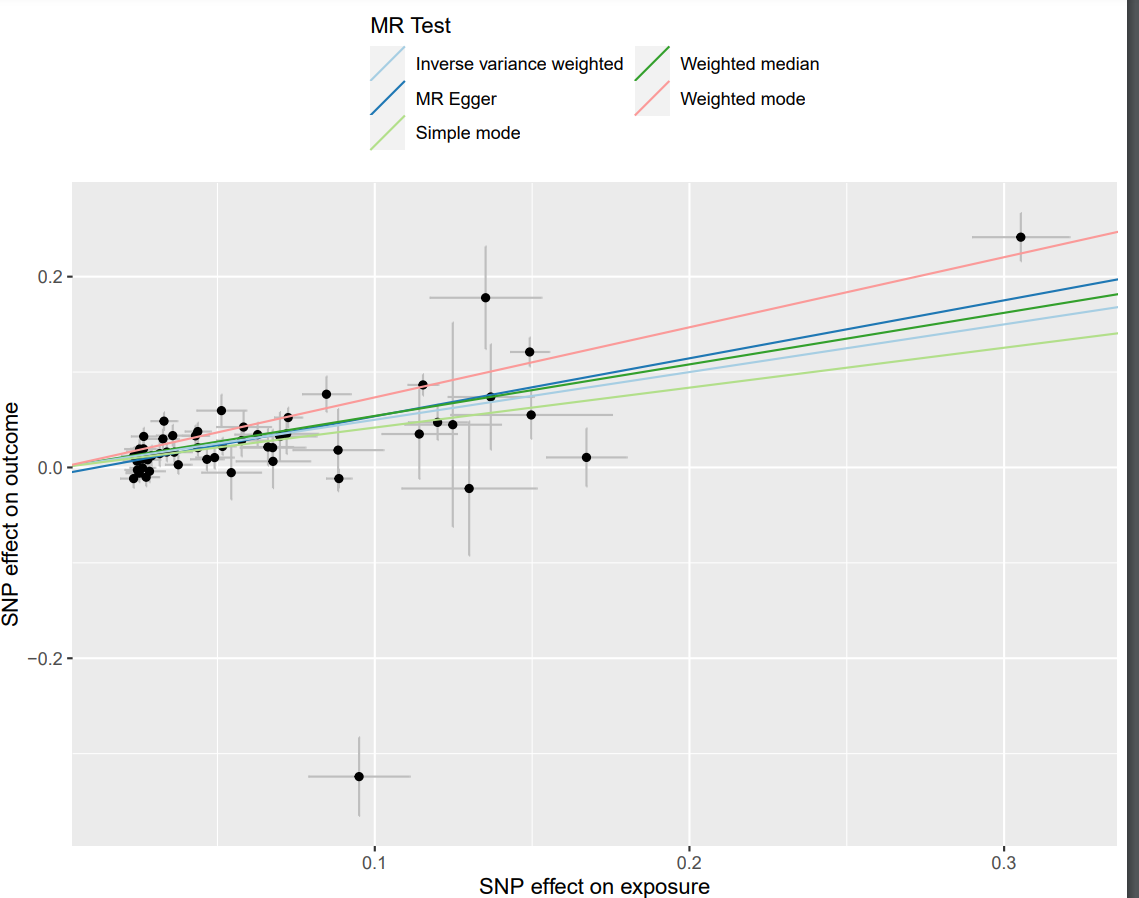
C D**

**Unstable angina pectoris**

**A B**

**
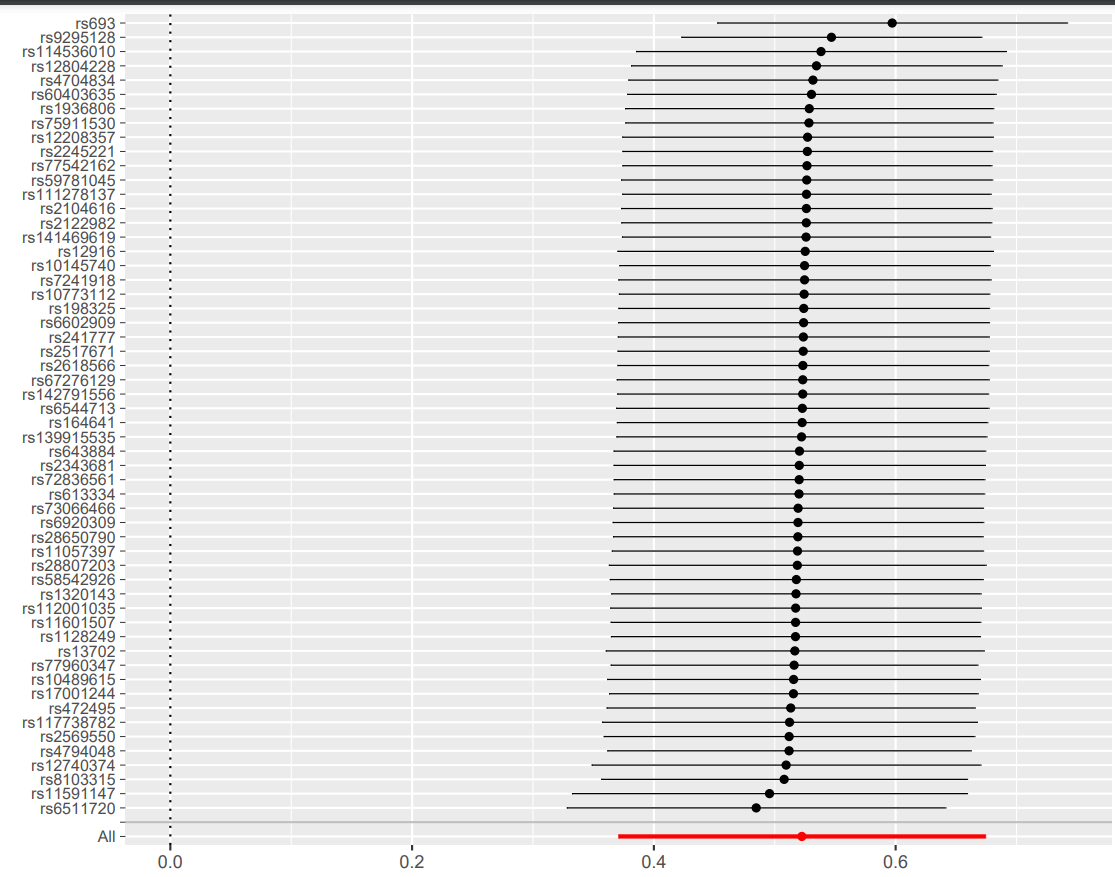

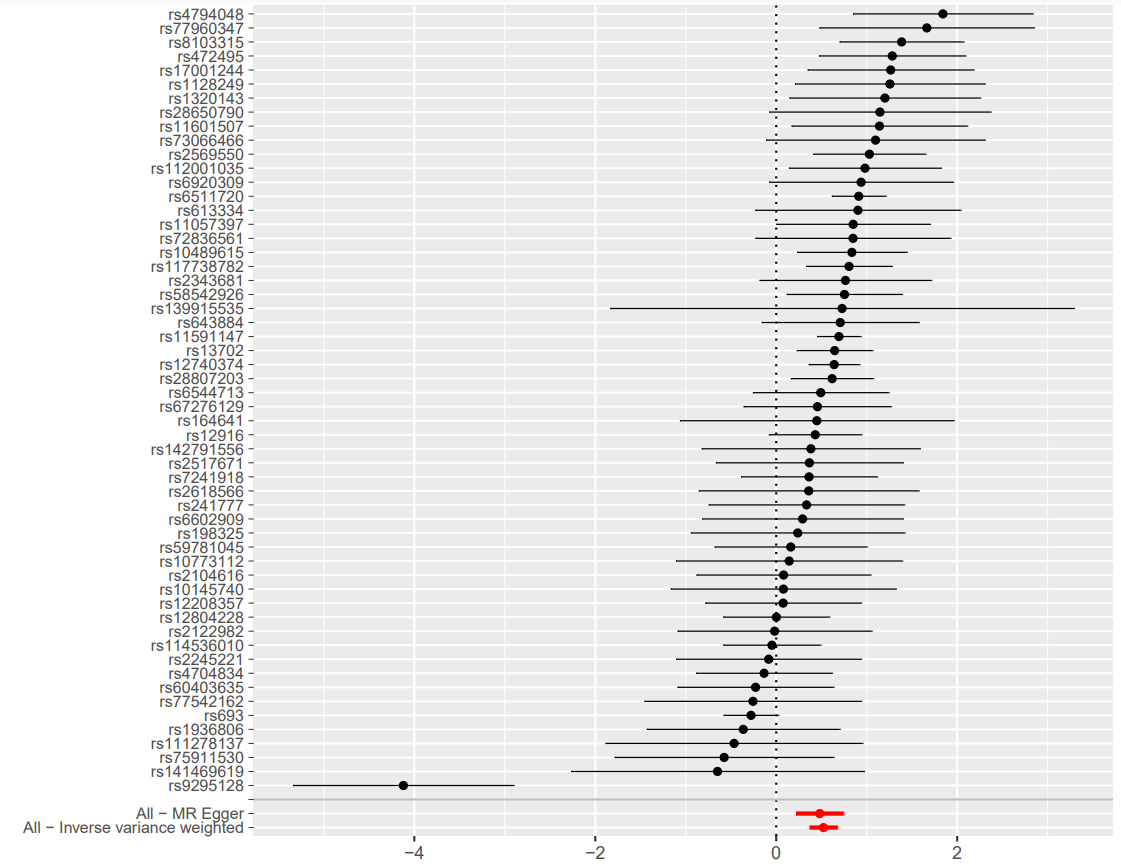
**

**
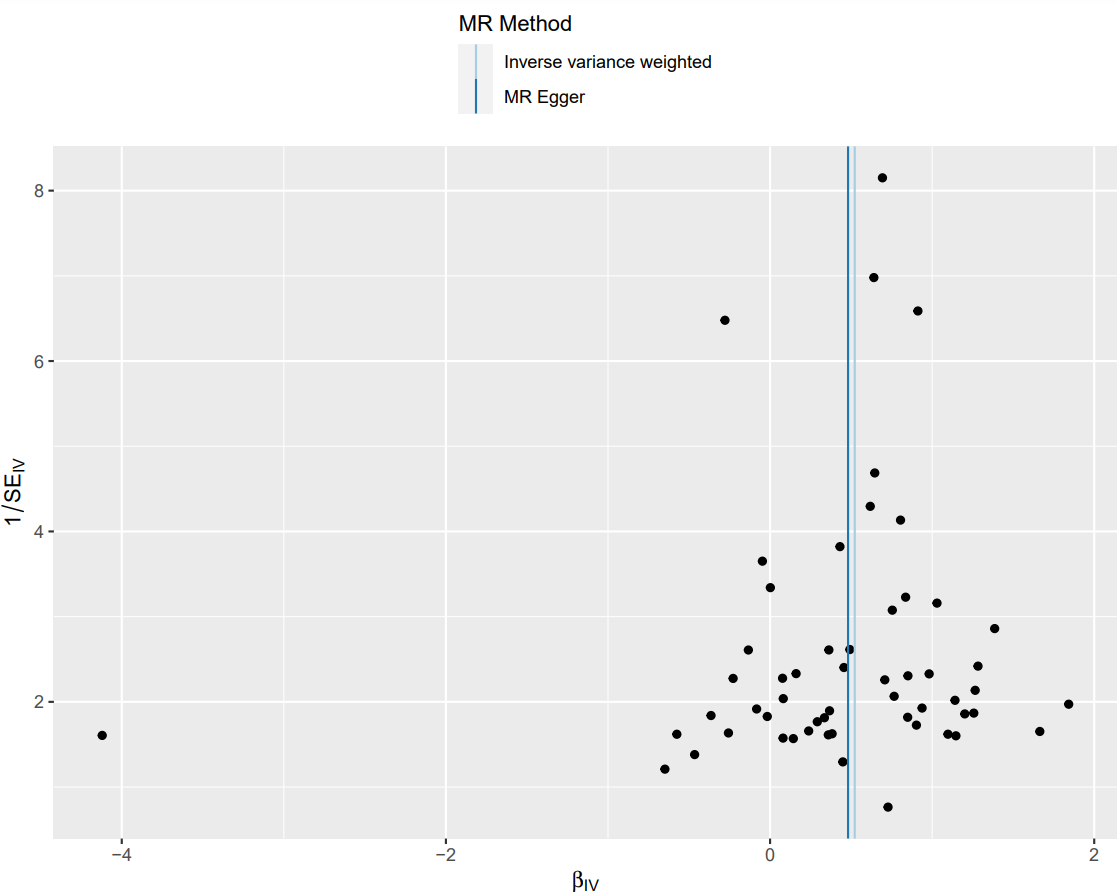

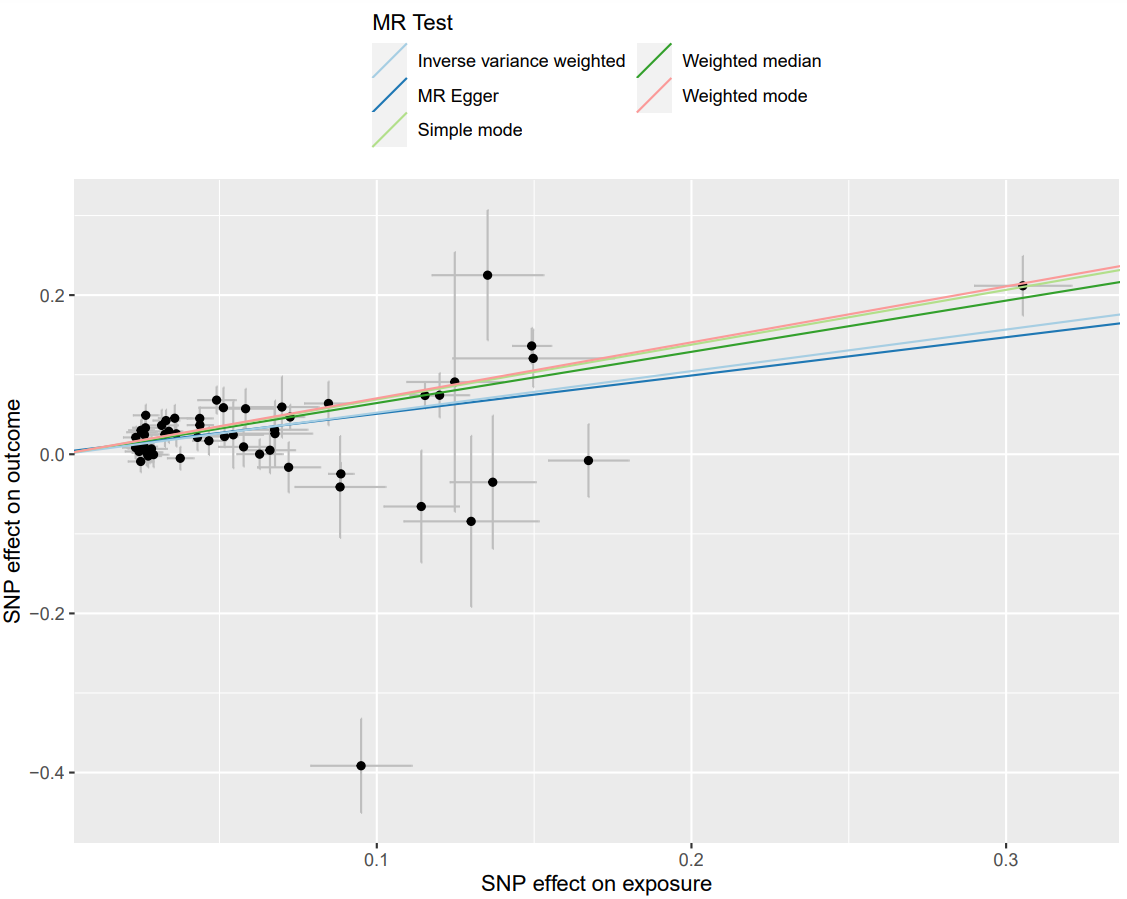
C D**

**Myocardial infarction**

**
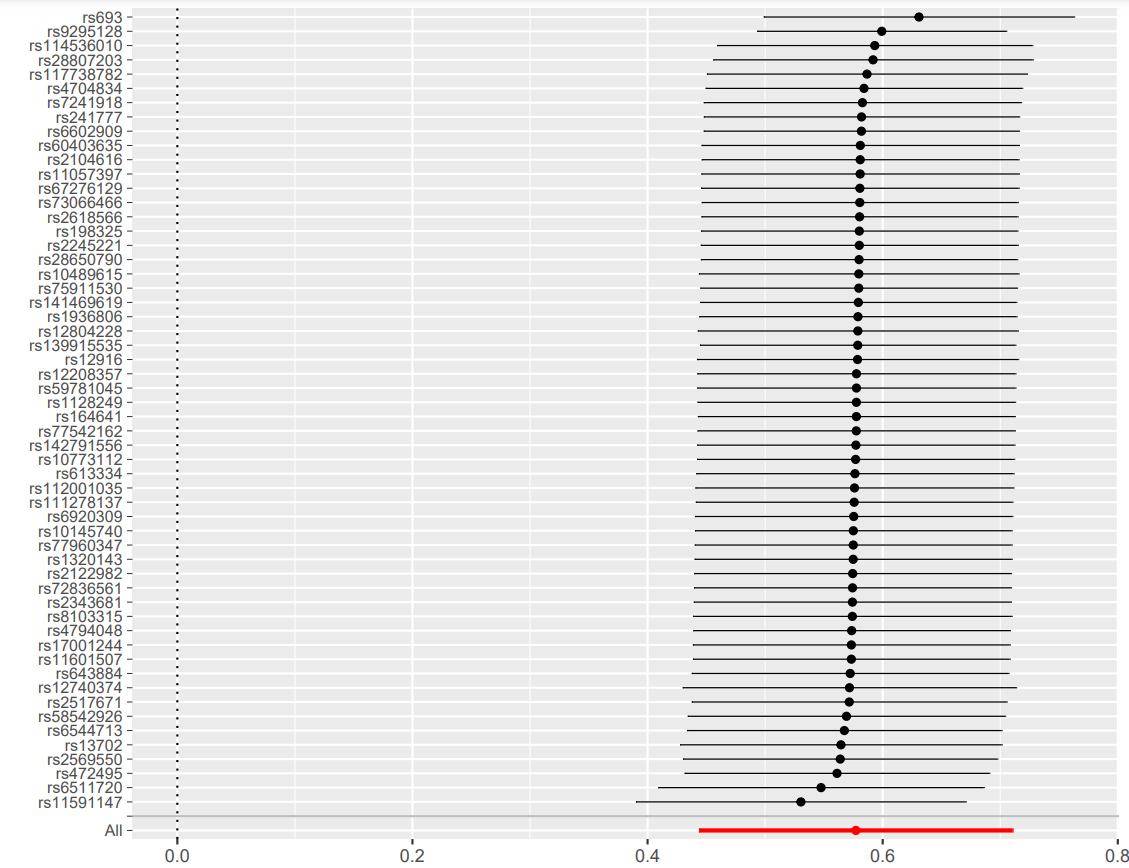

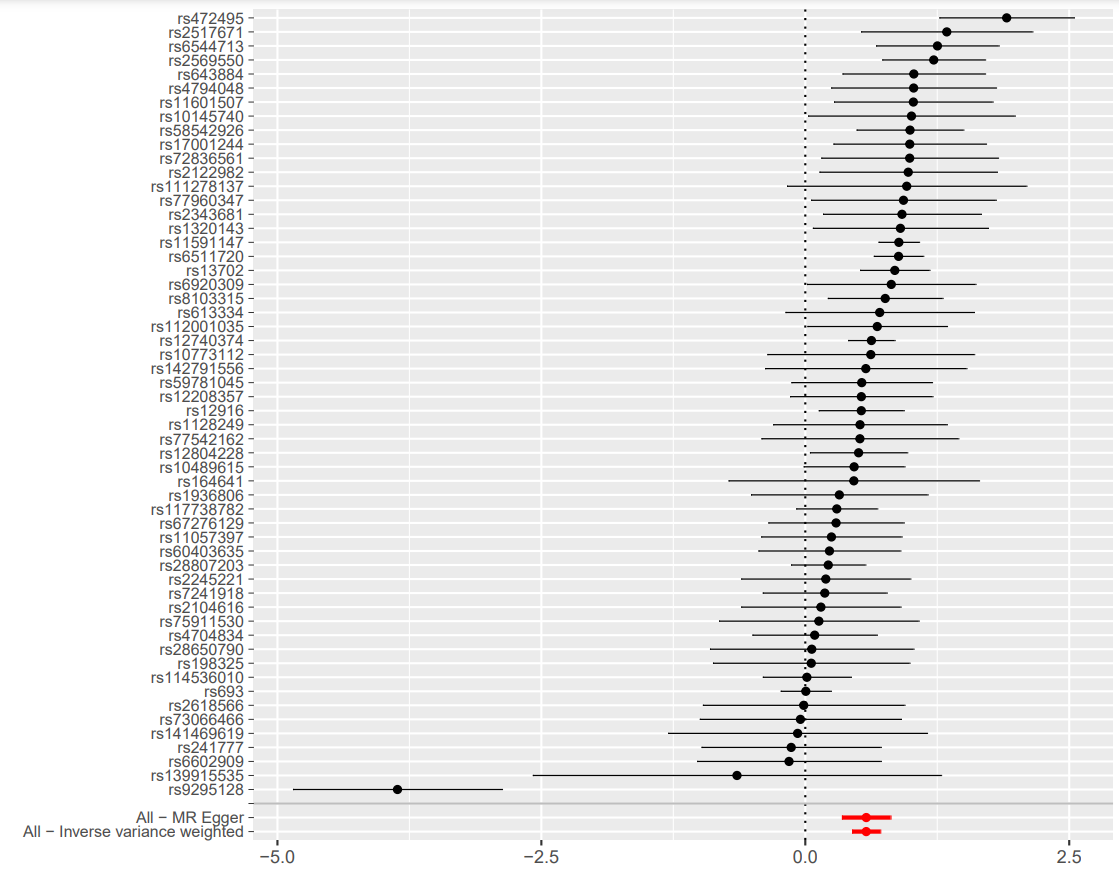
A B**

**
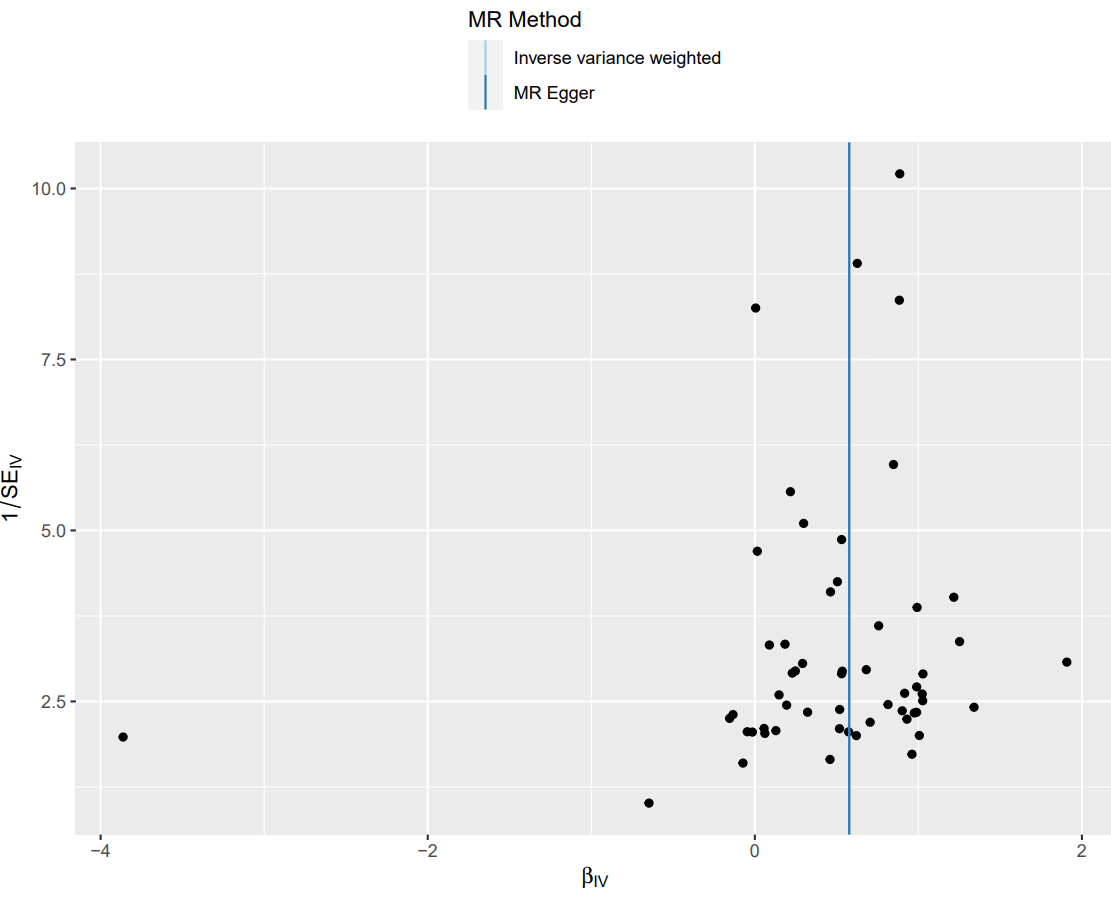

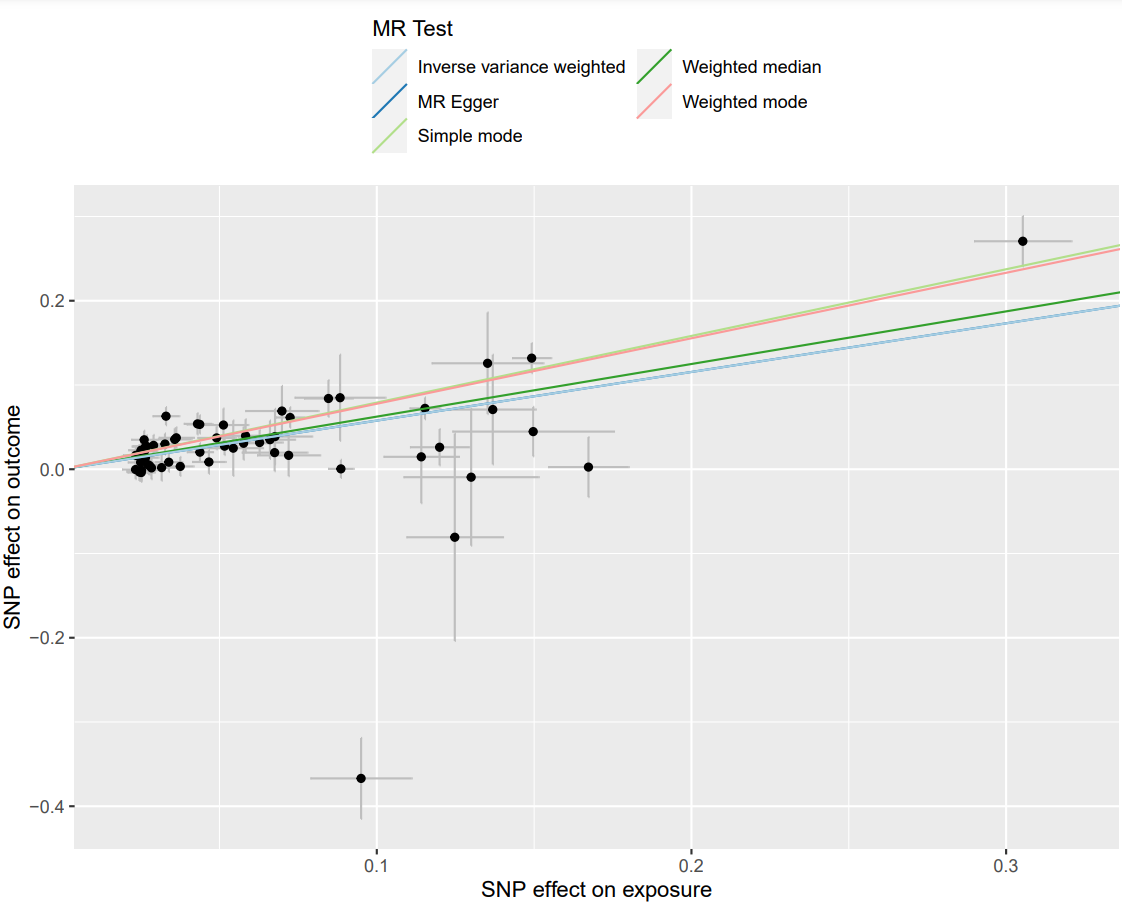
C D**

**Major coronary heart disease event**

**
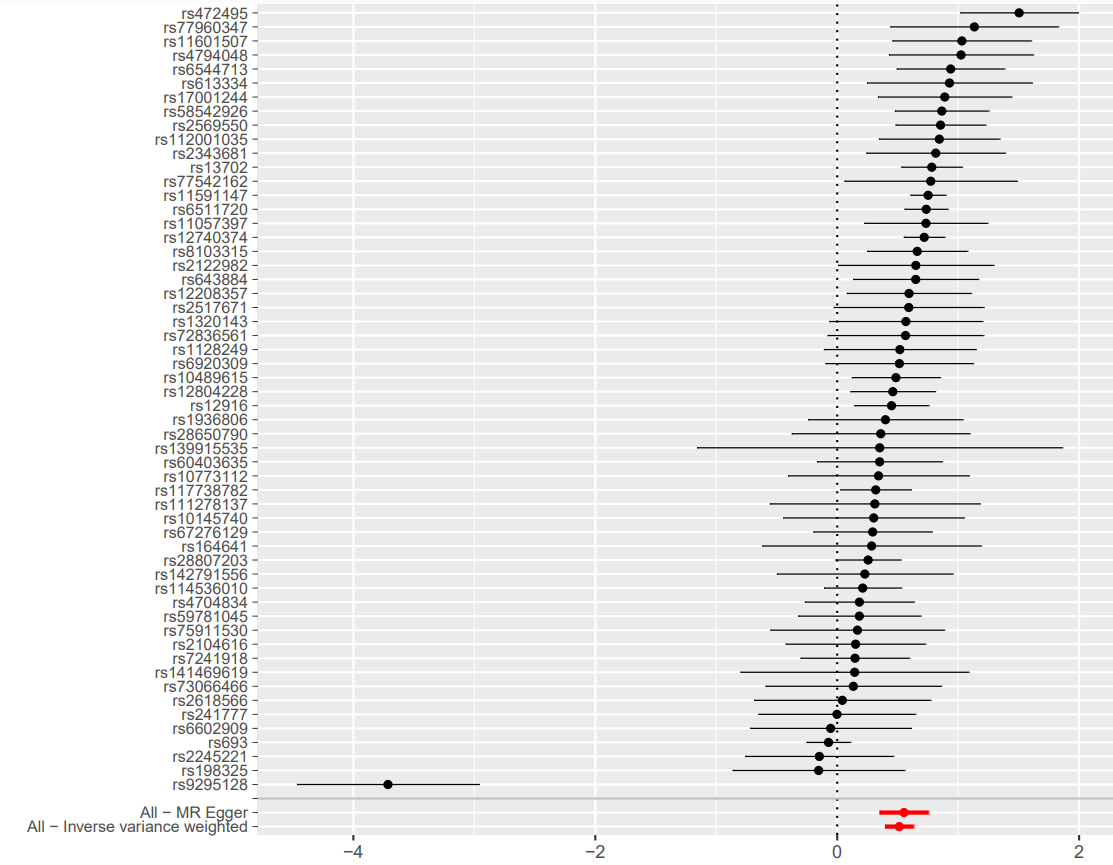

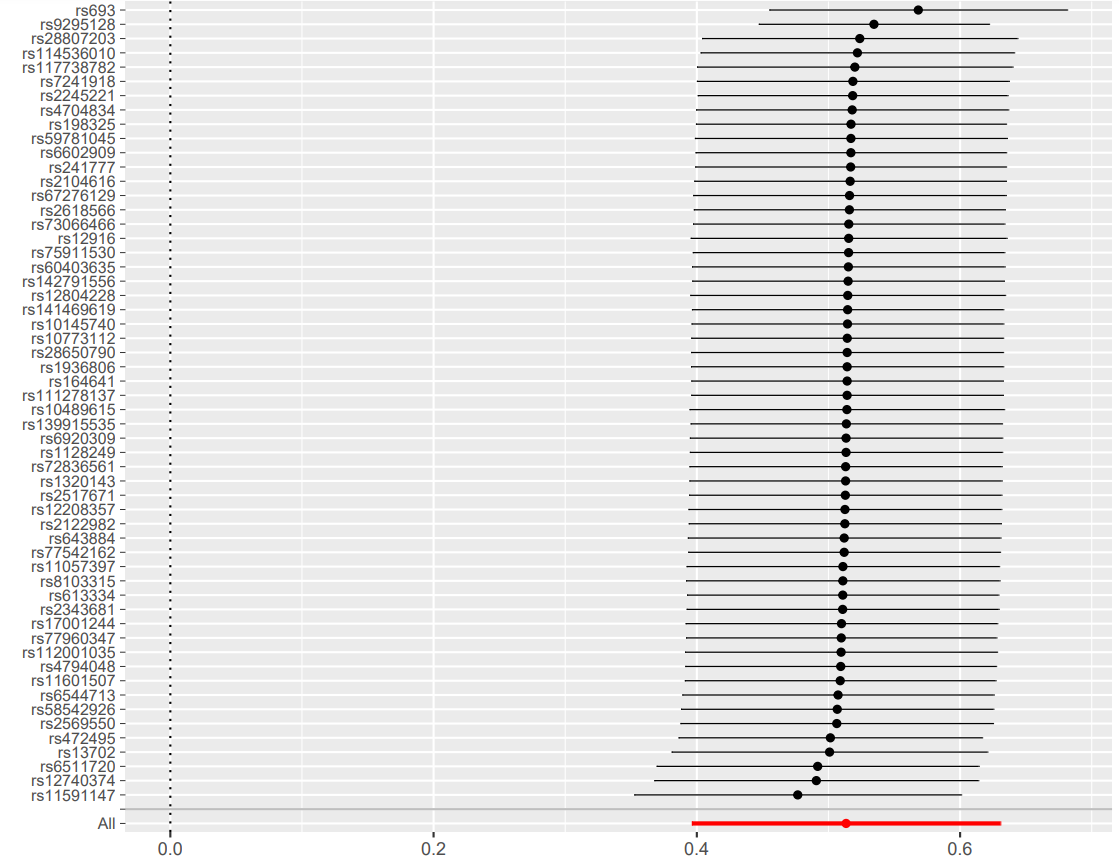
A B**

**
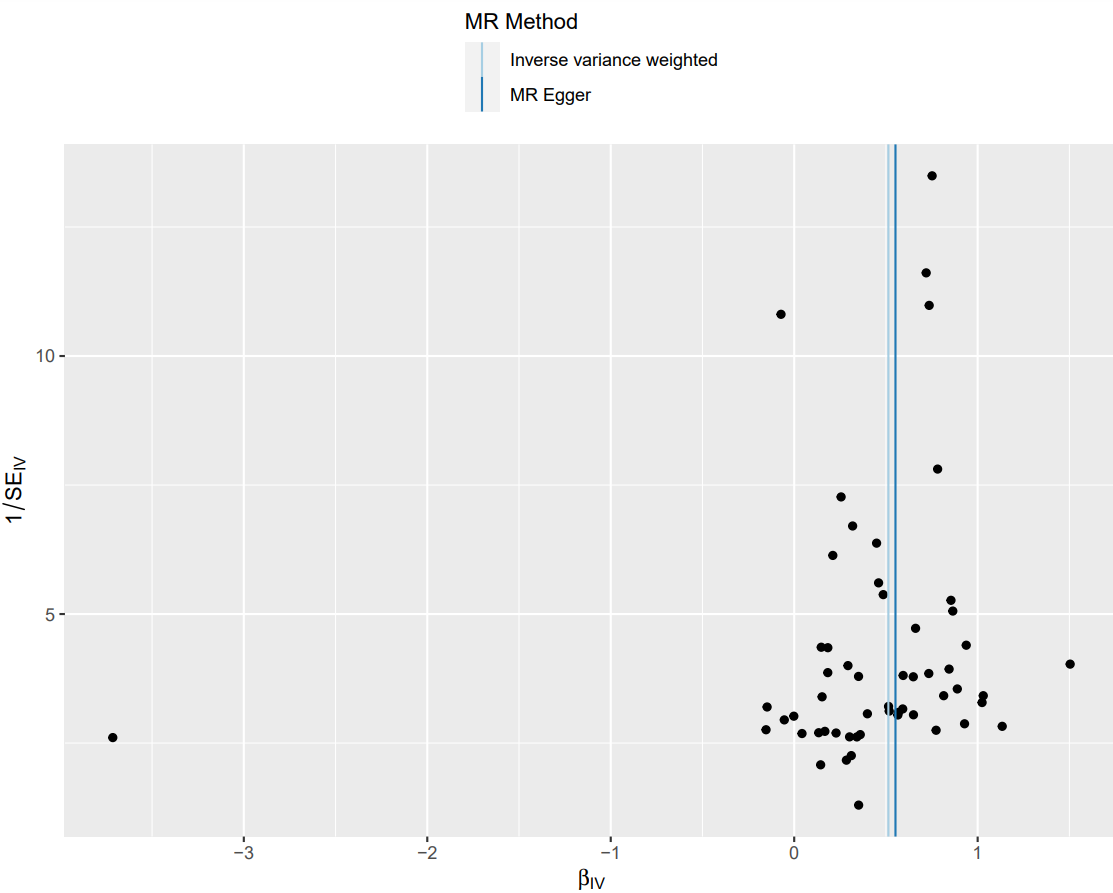

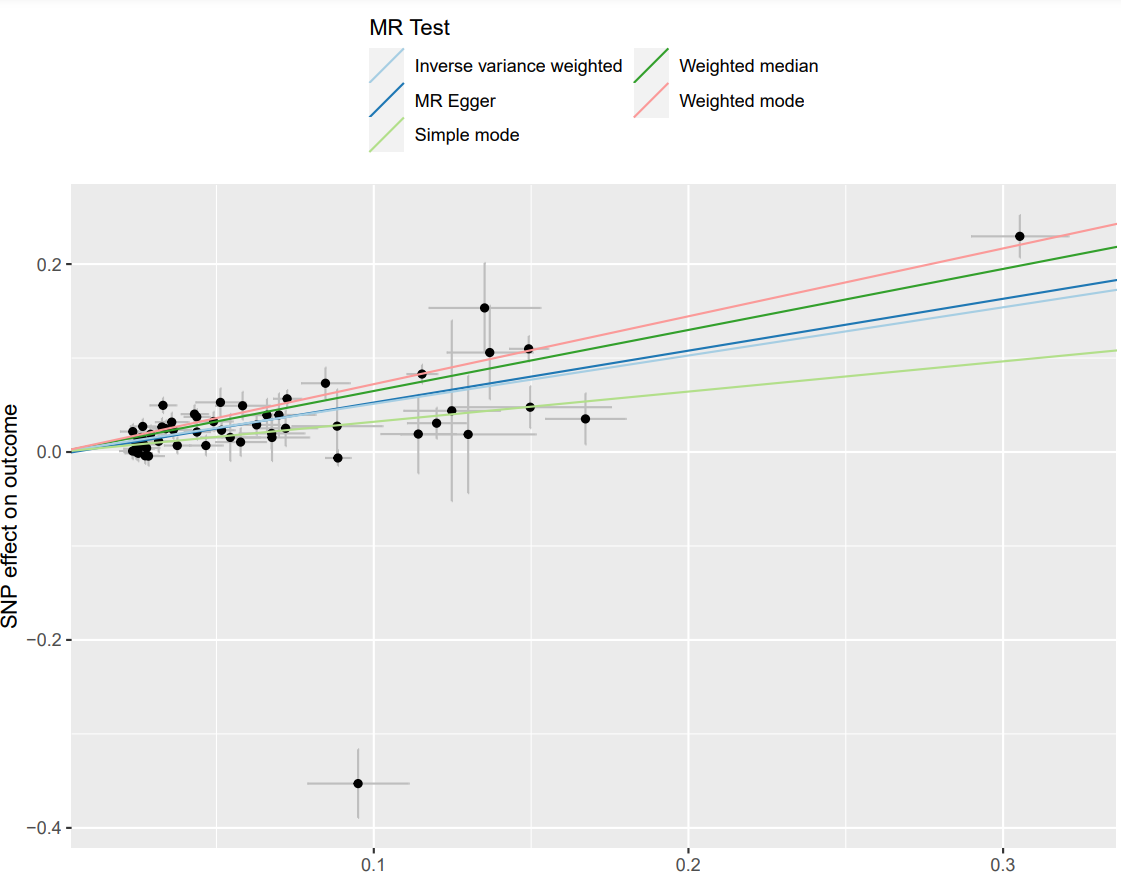
C D**

**Aortic aneurysm**

**
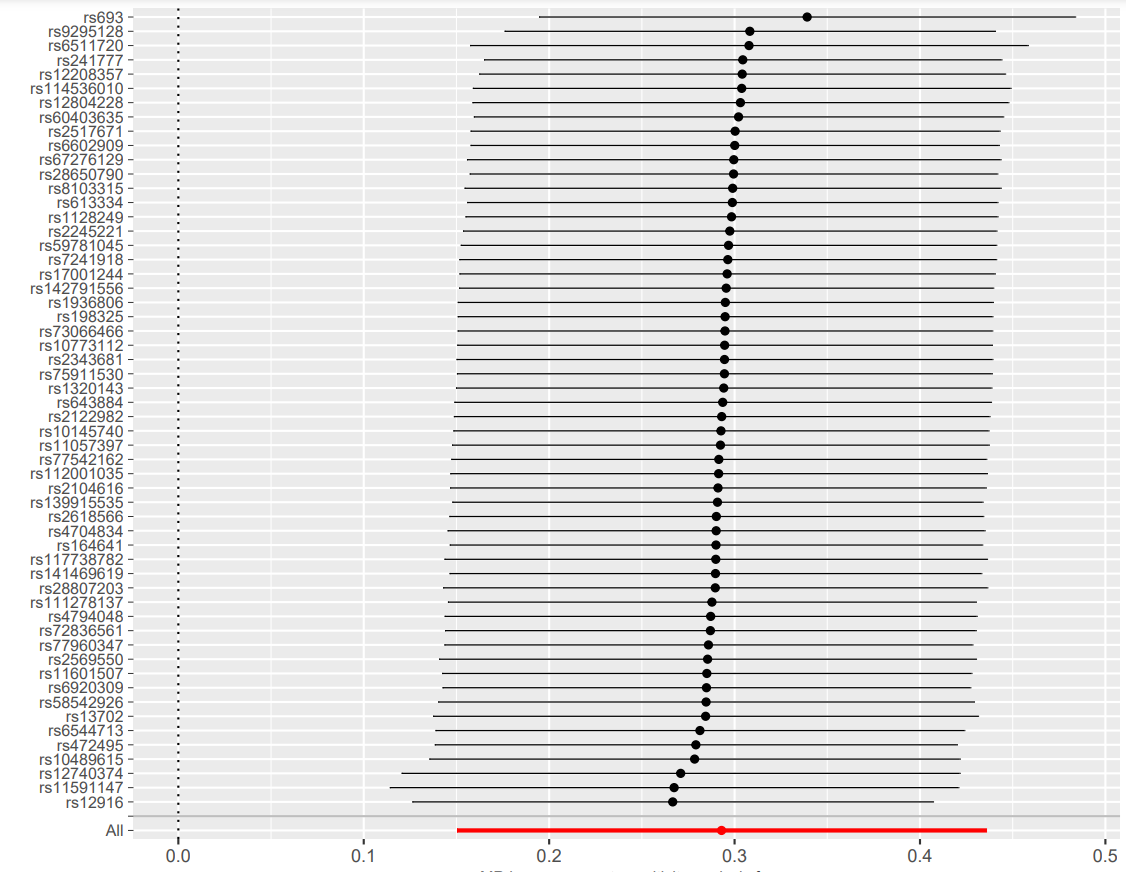

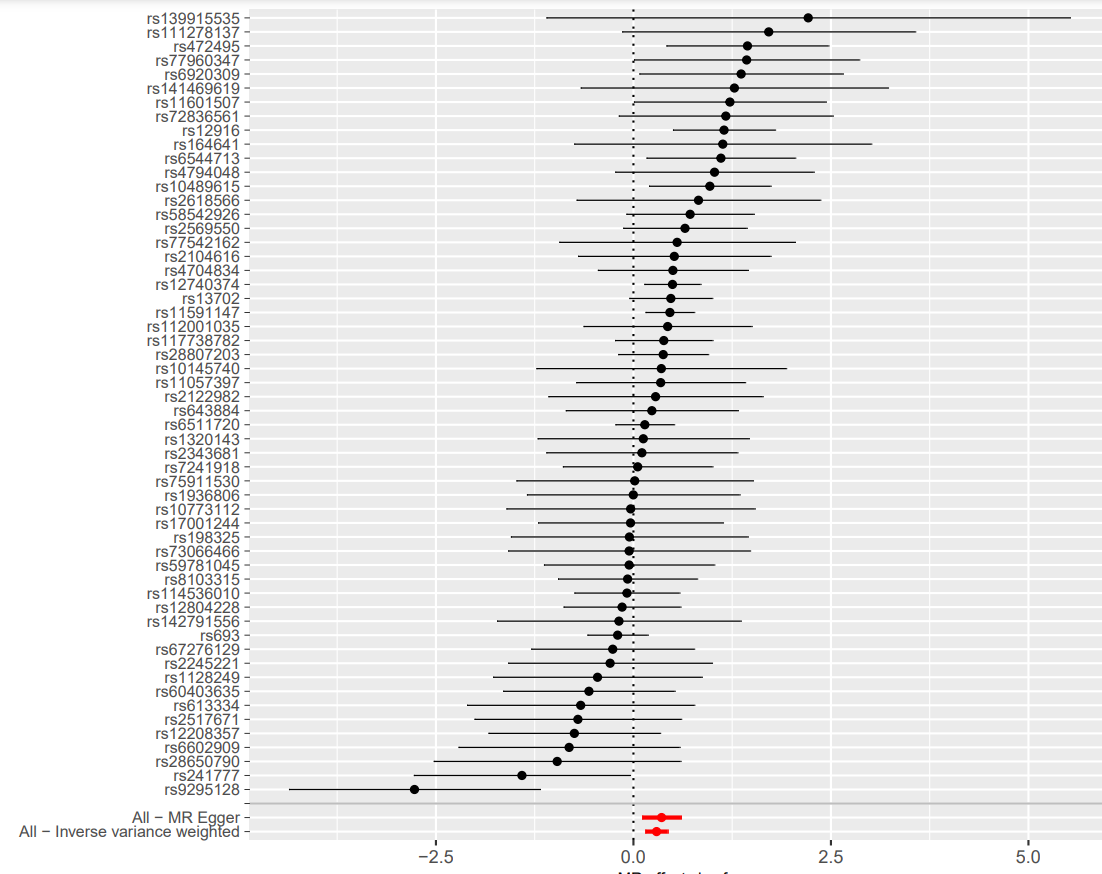
A** **B**

**
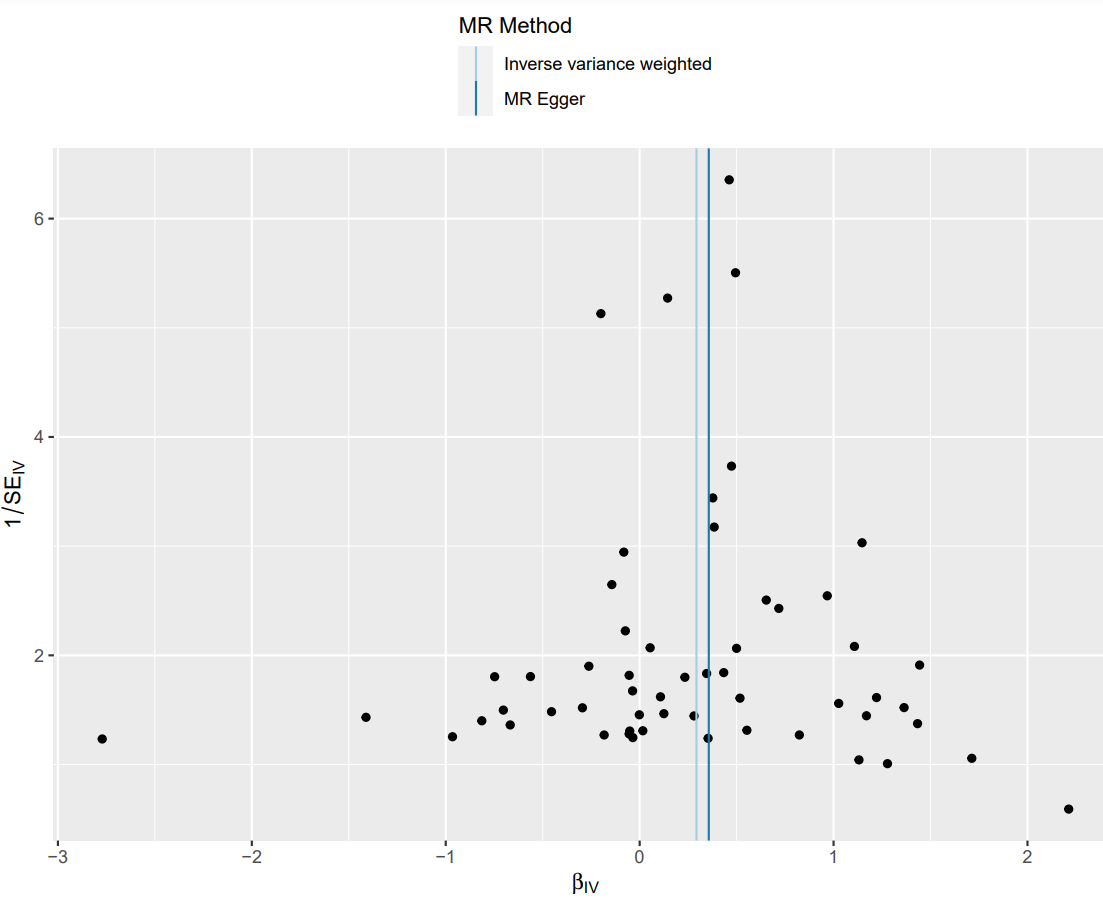

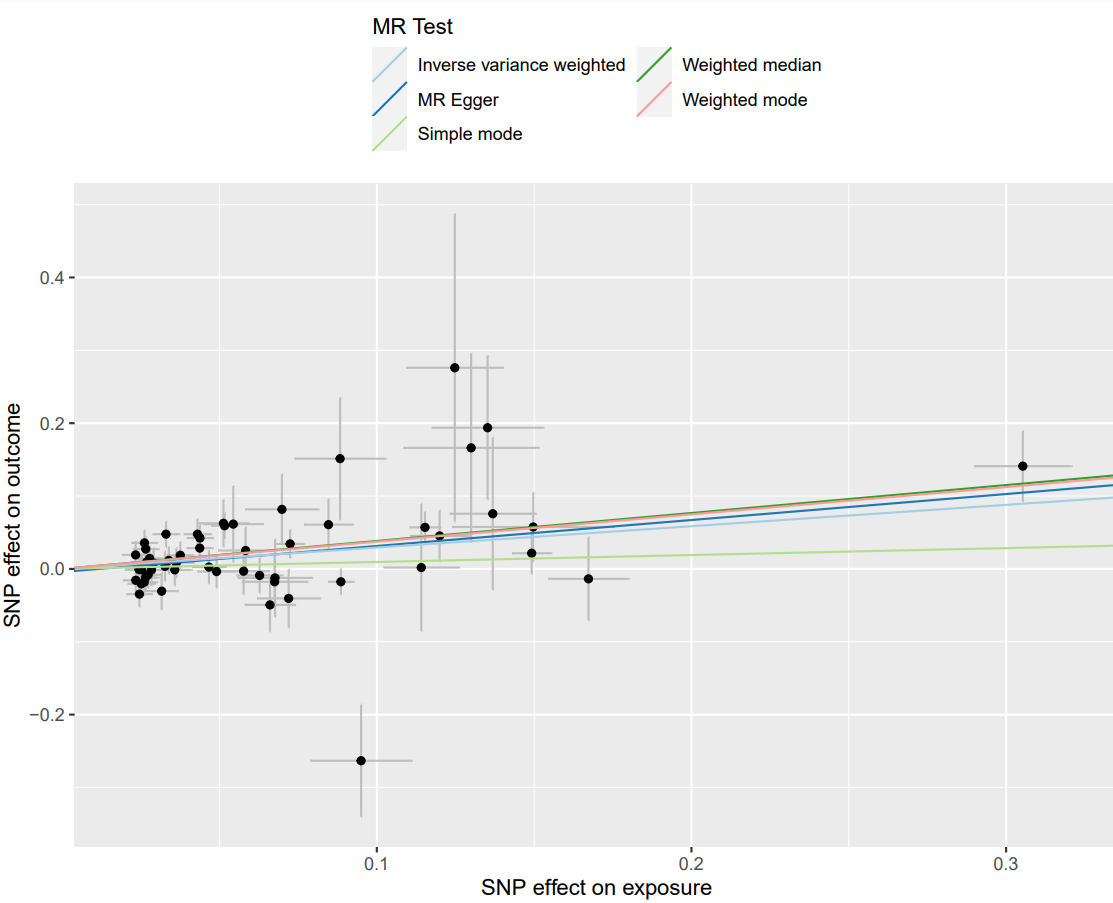
C D**

**Abdominal aortic aneurysm (AAA)**

**
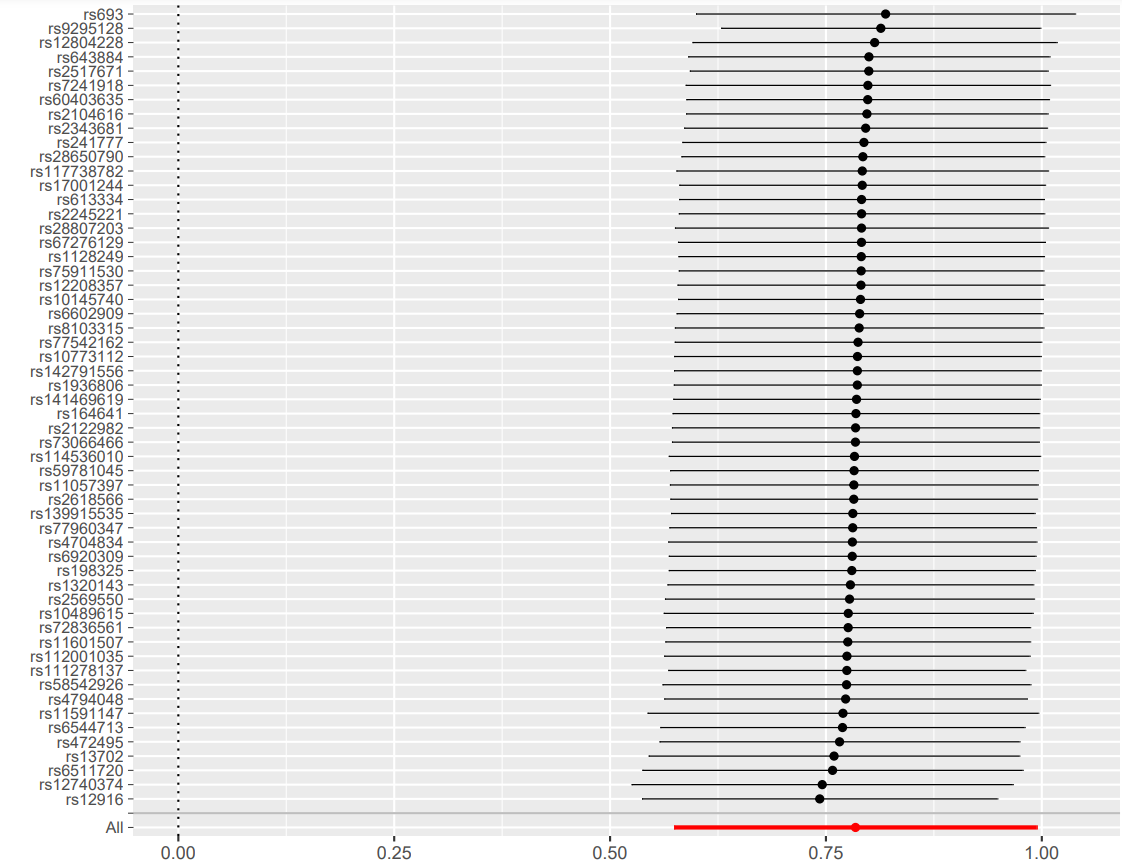

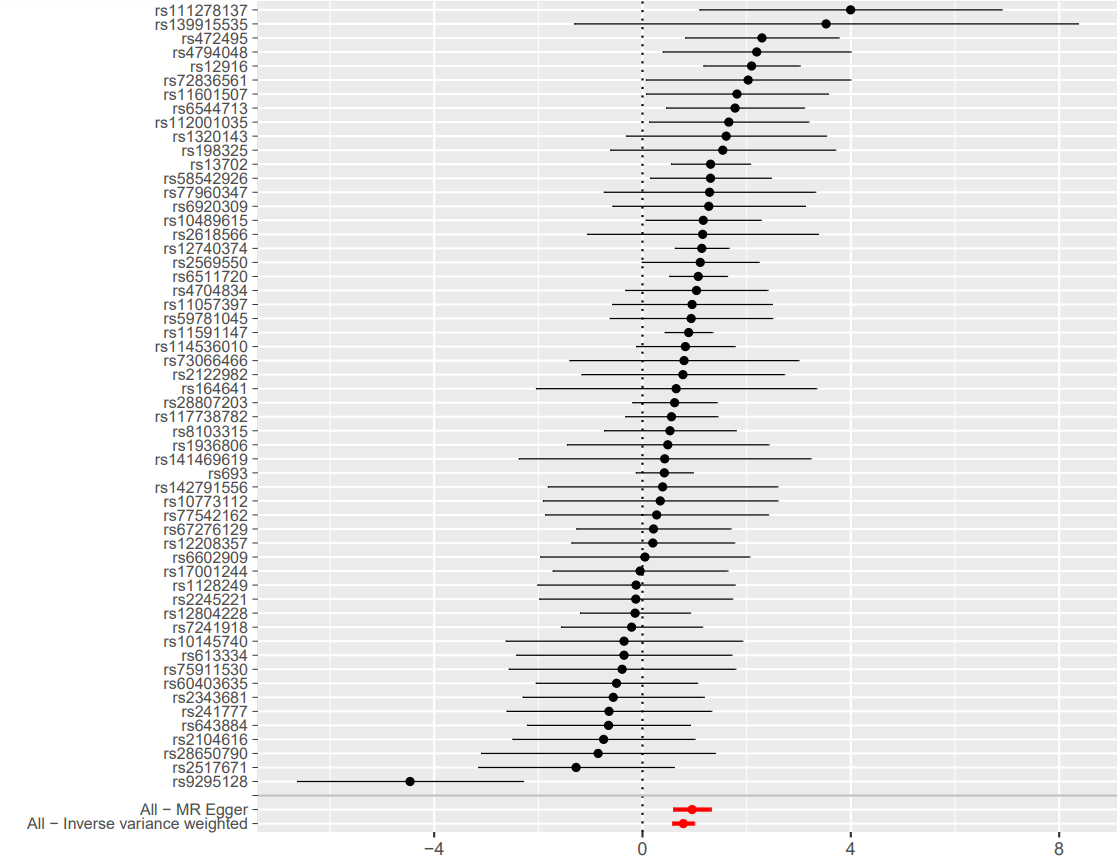
A B**

**
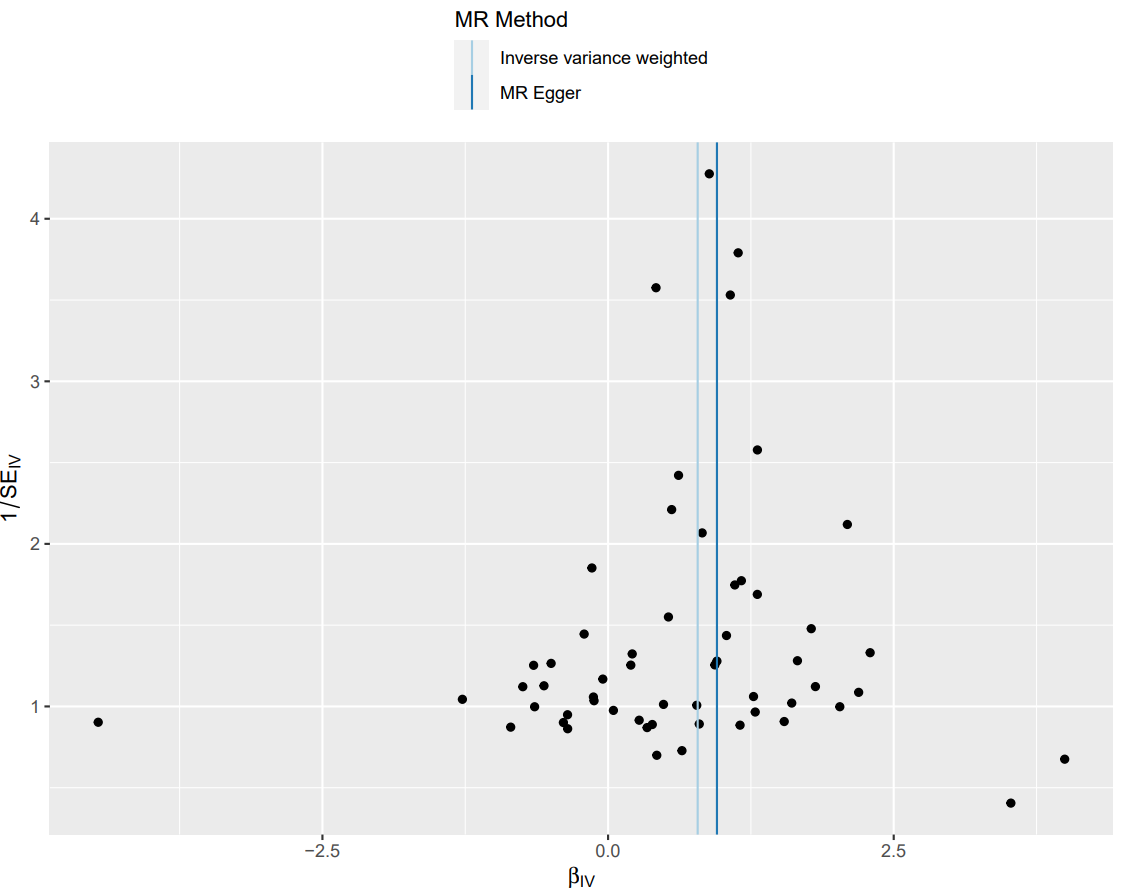

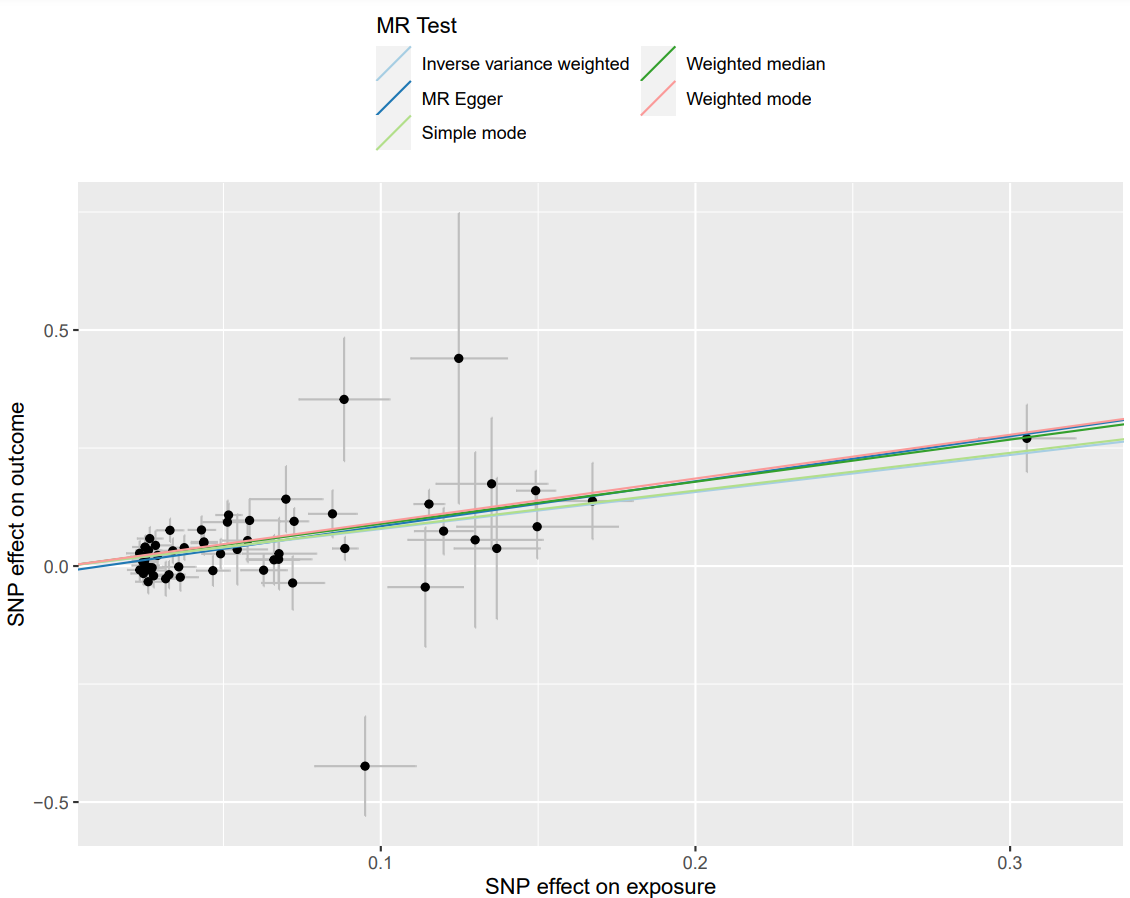
C D**

**Hypertension**

**
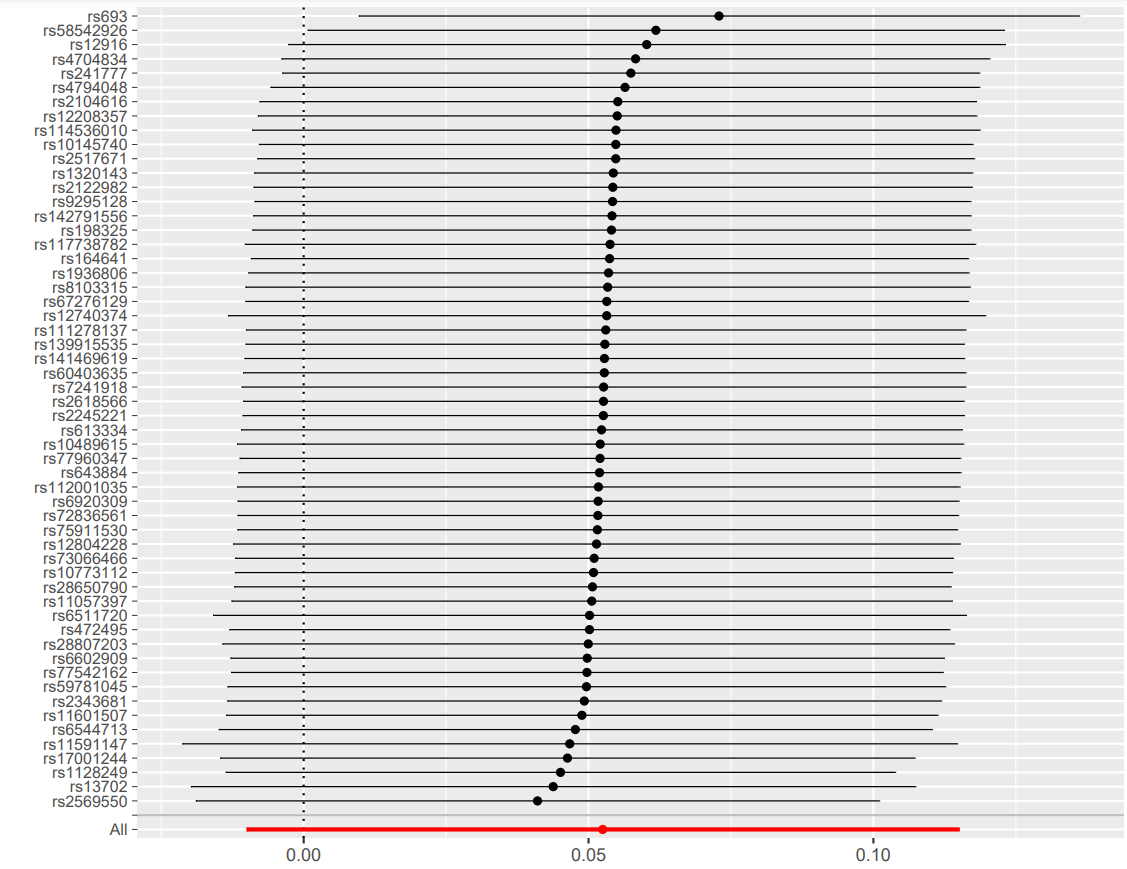
**

**
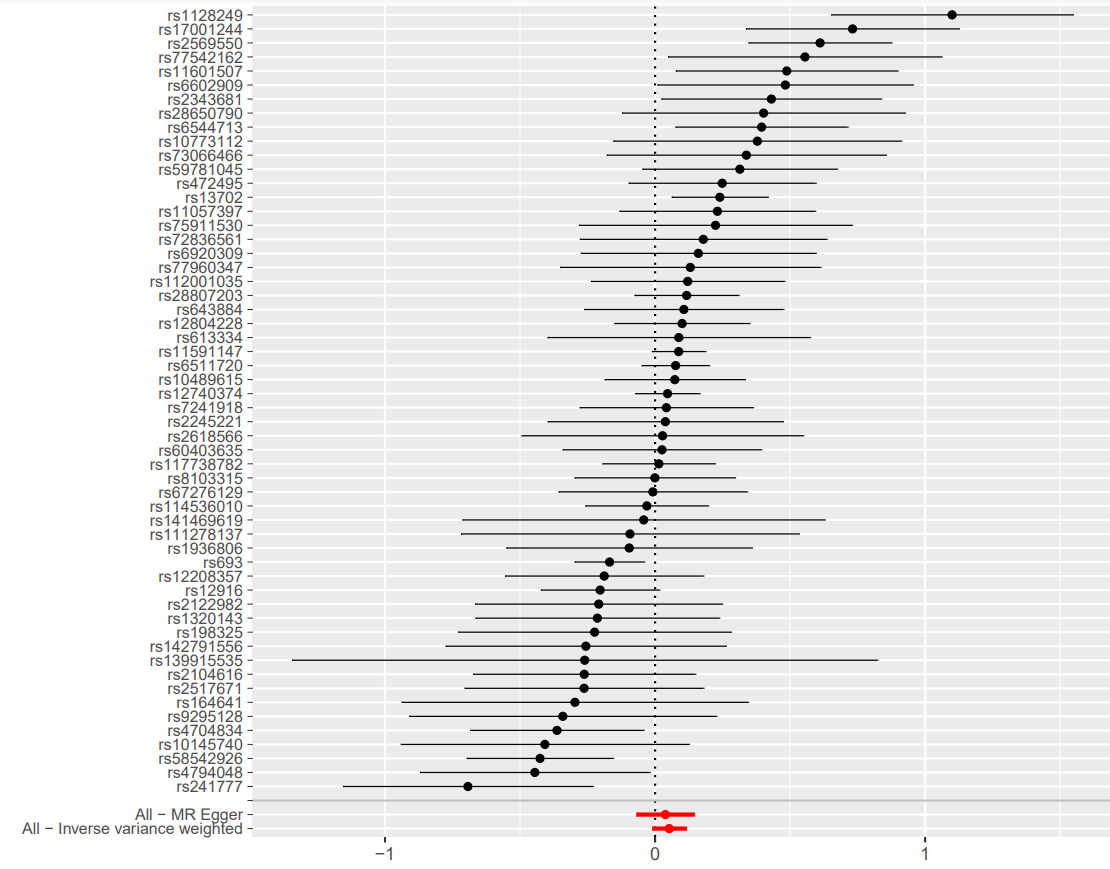
A B**

**
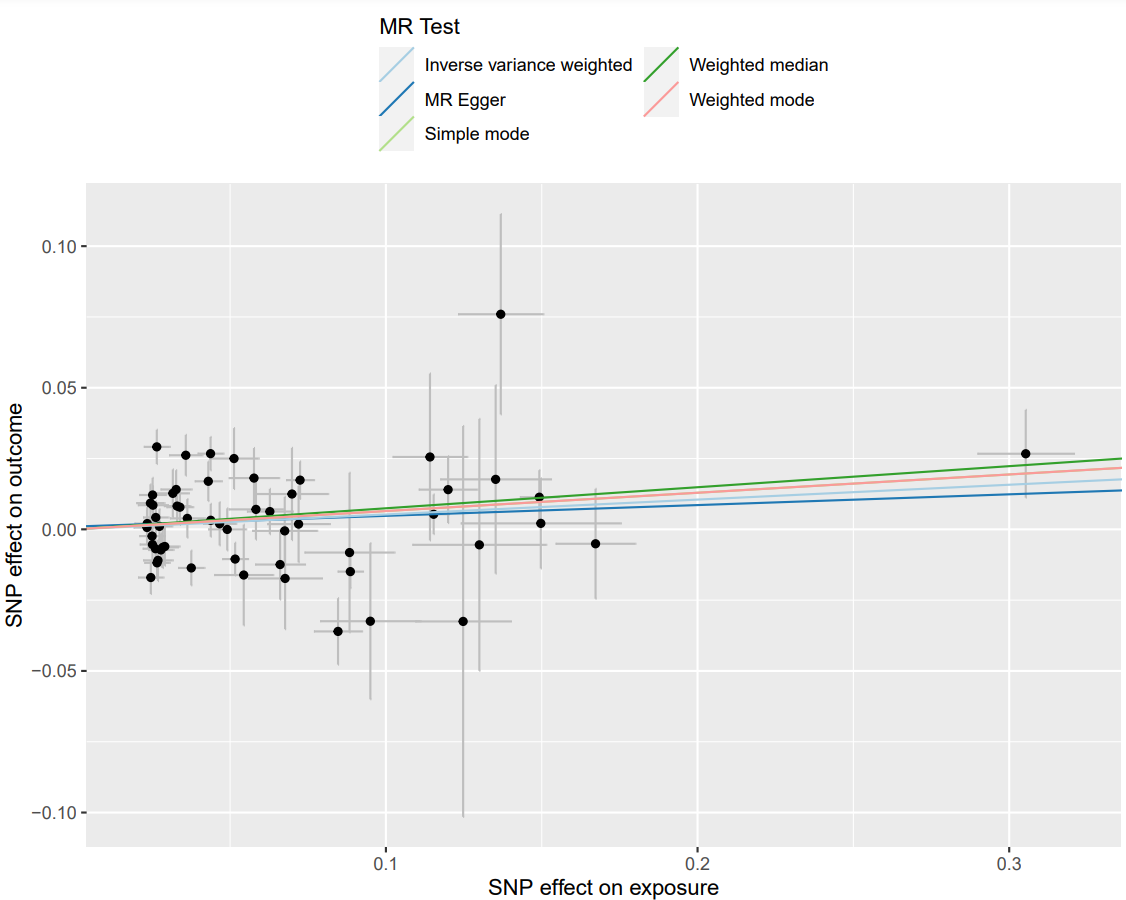
C D**

**Non-rheumatic valve diseases**

**
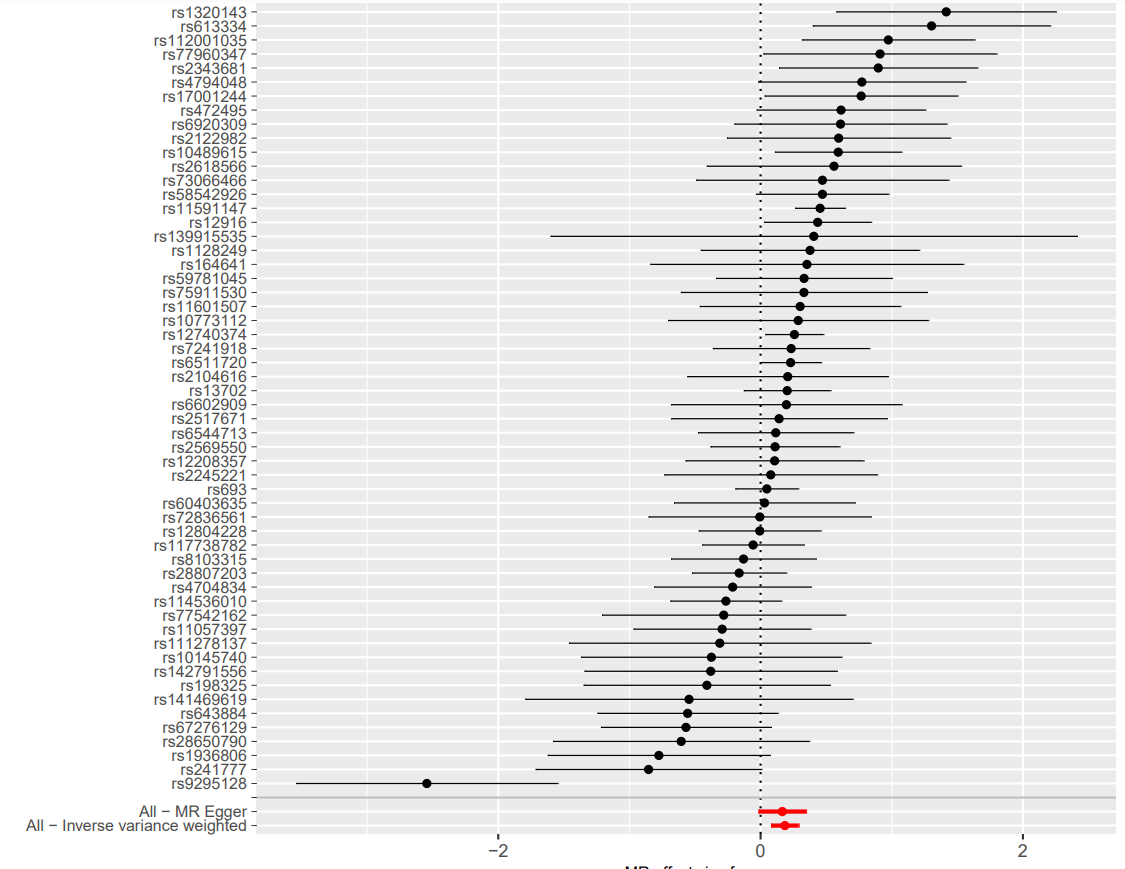

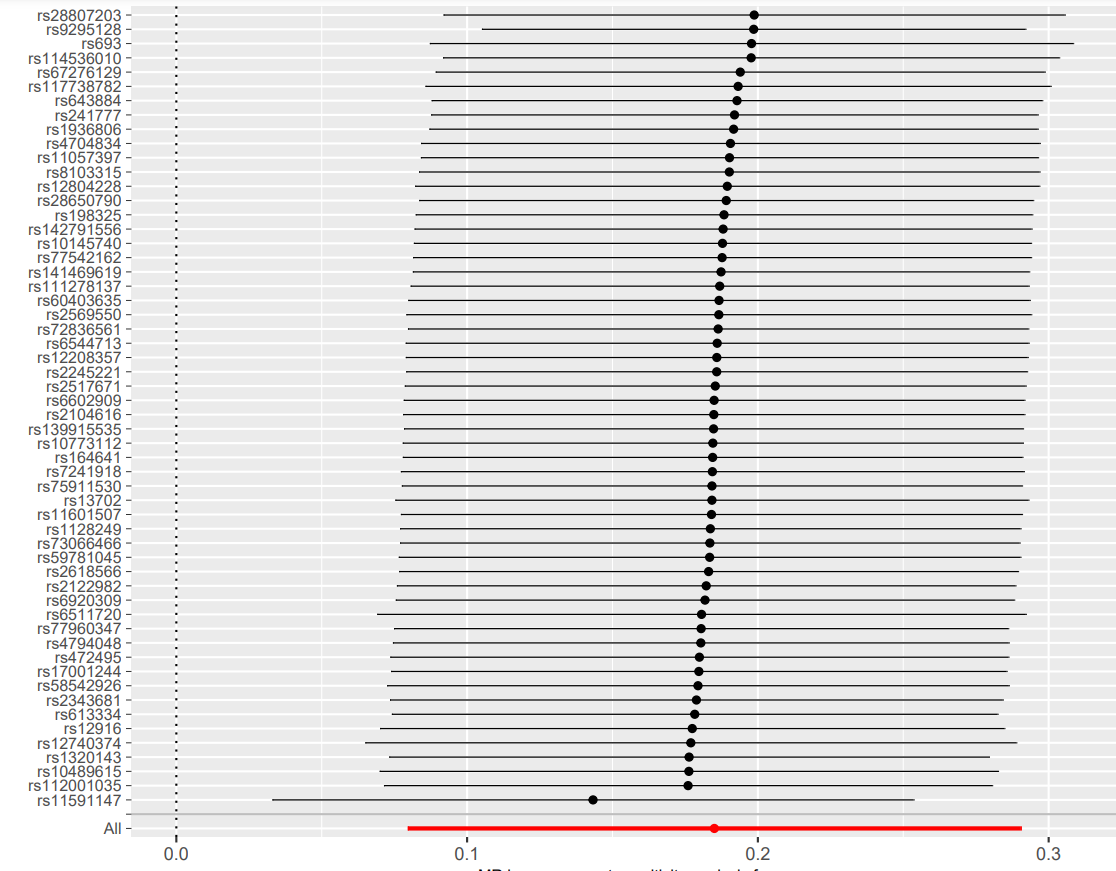

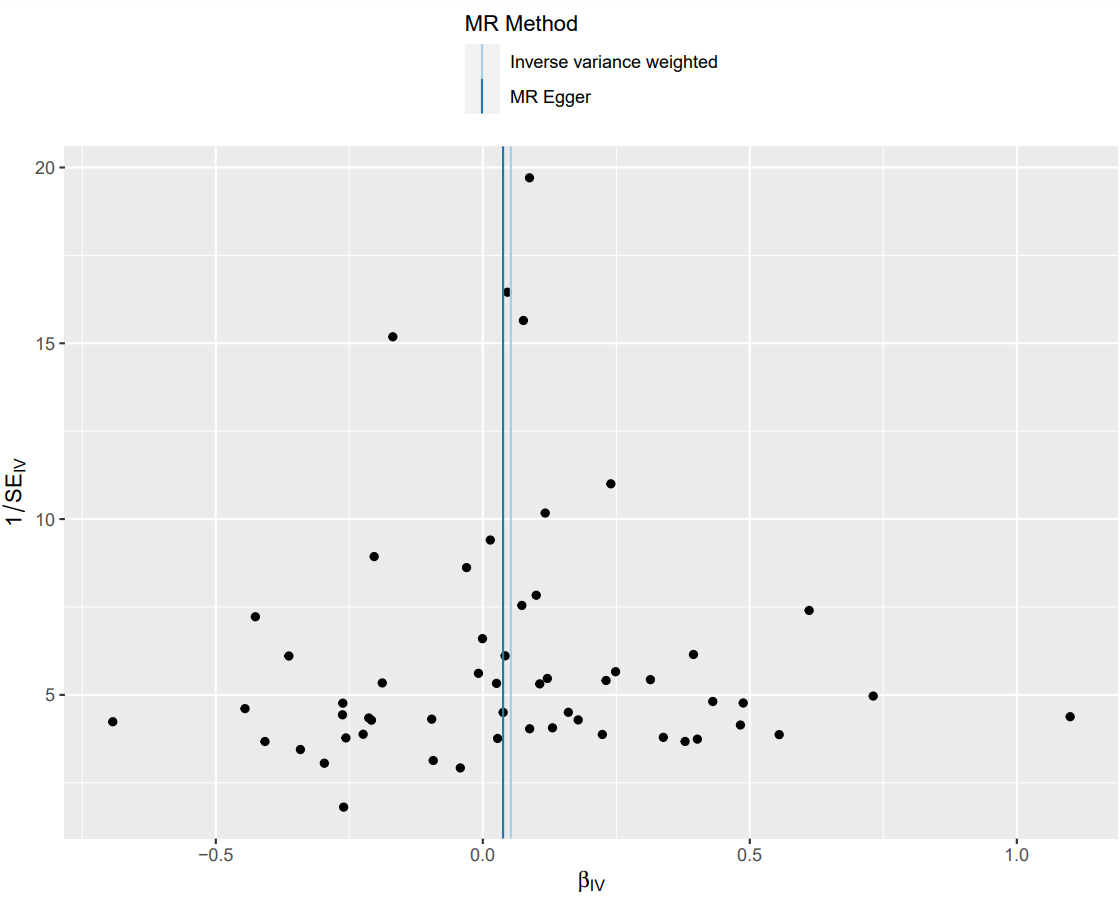
****A B**

**
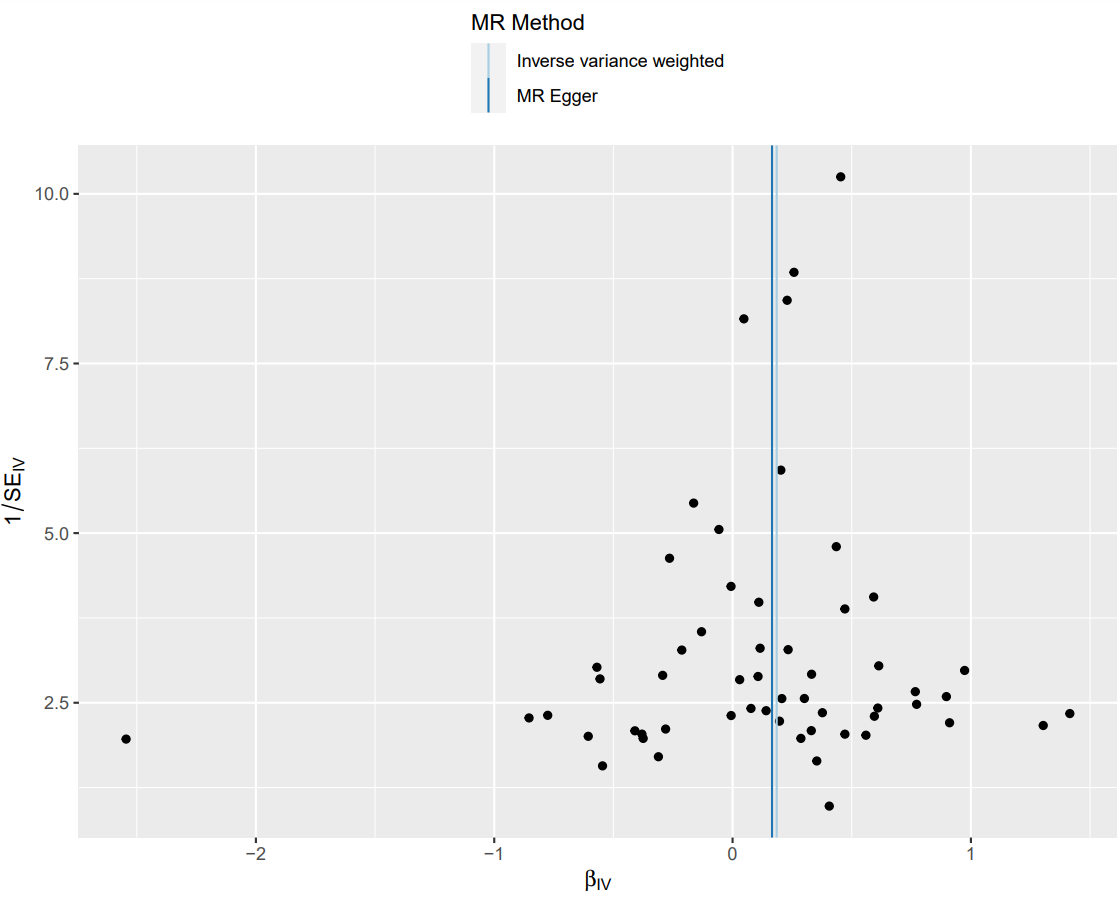

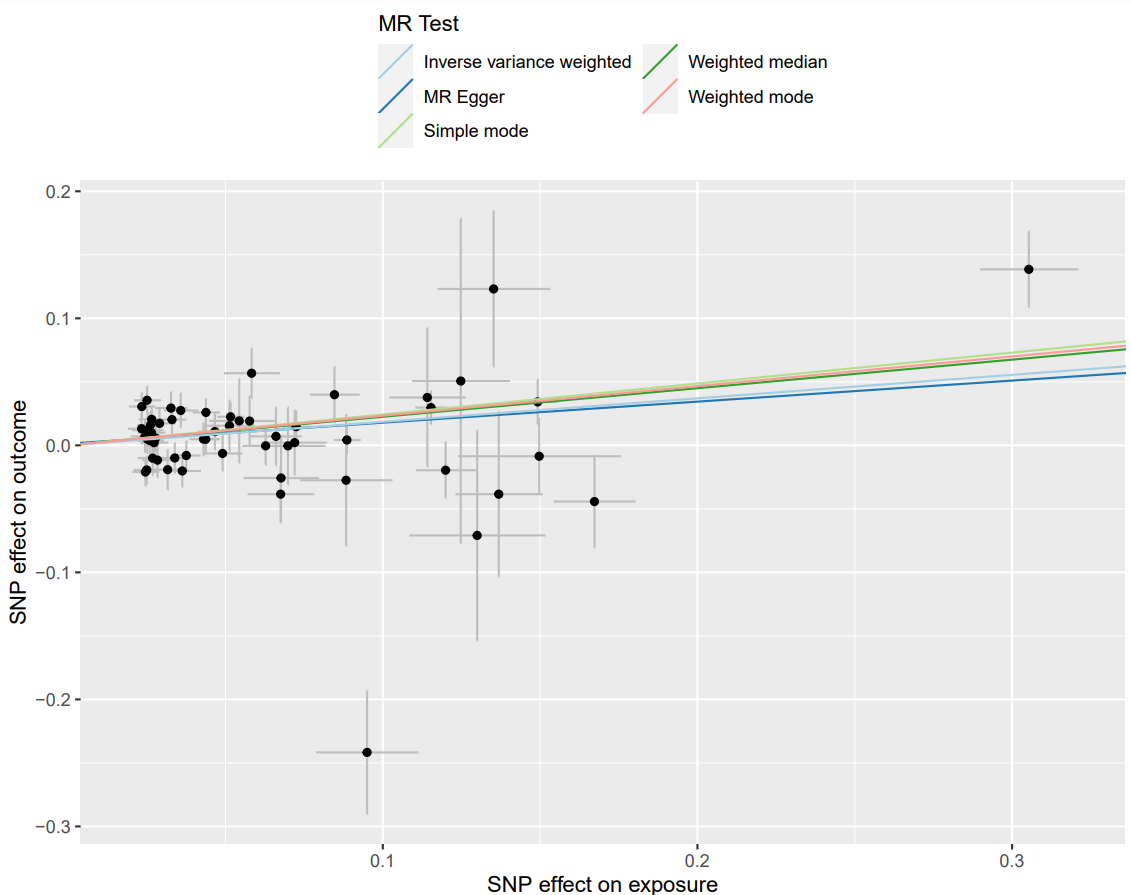
C D**

**Peripheral artery disease**

**
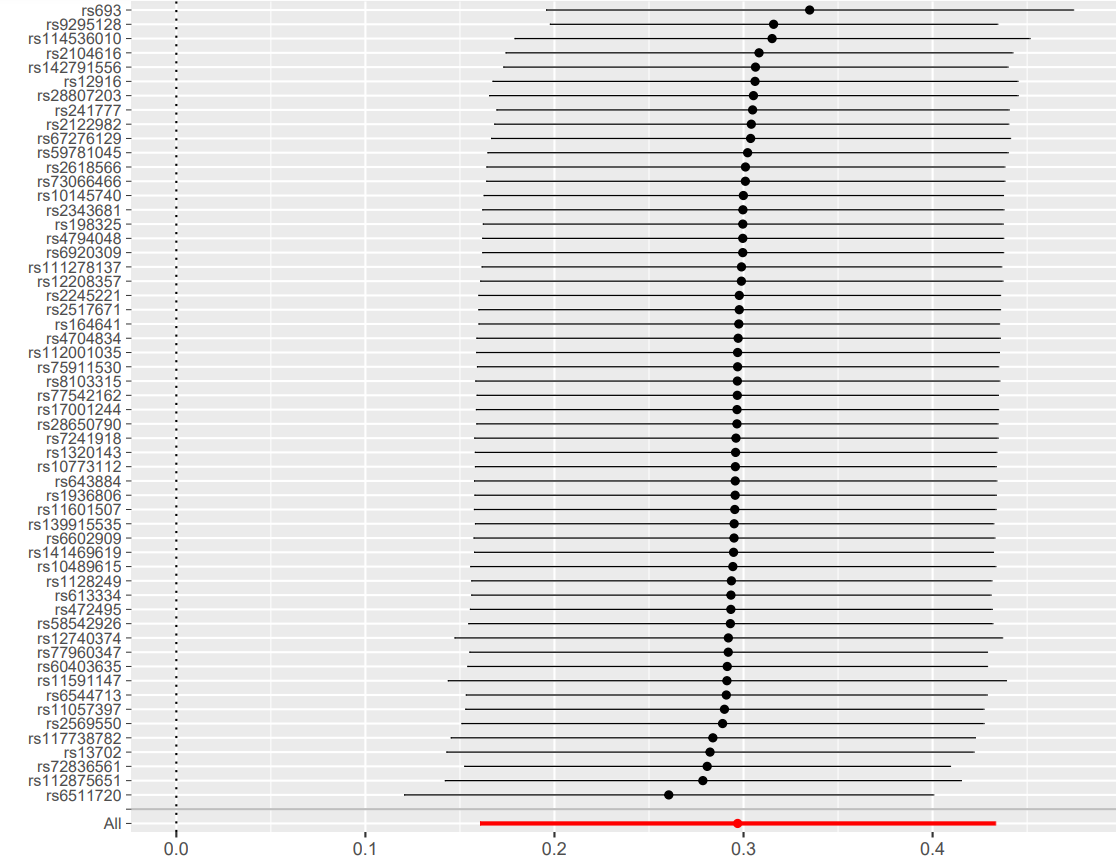

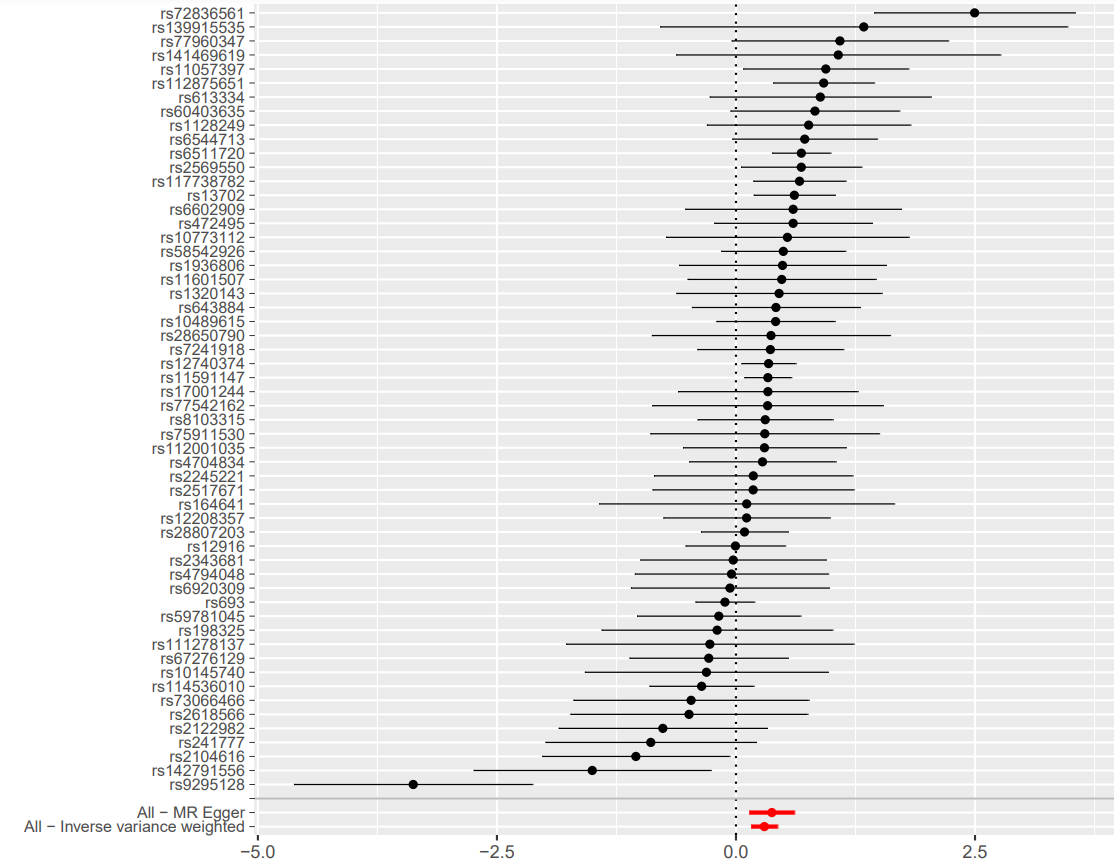
A B**

**
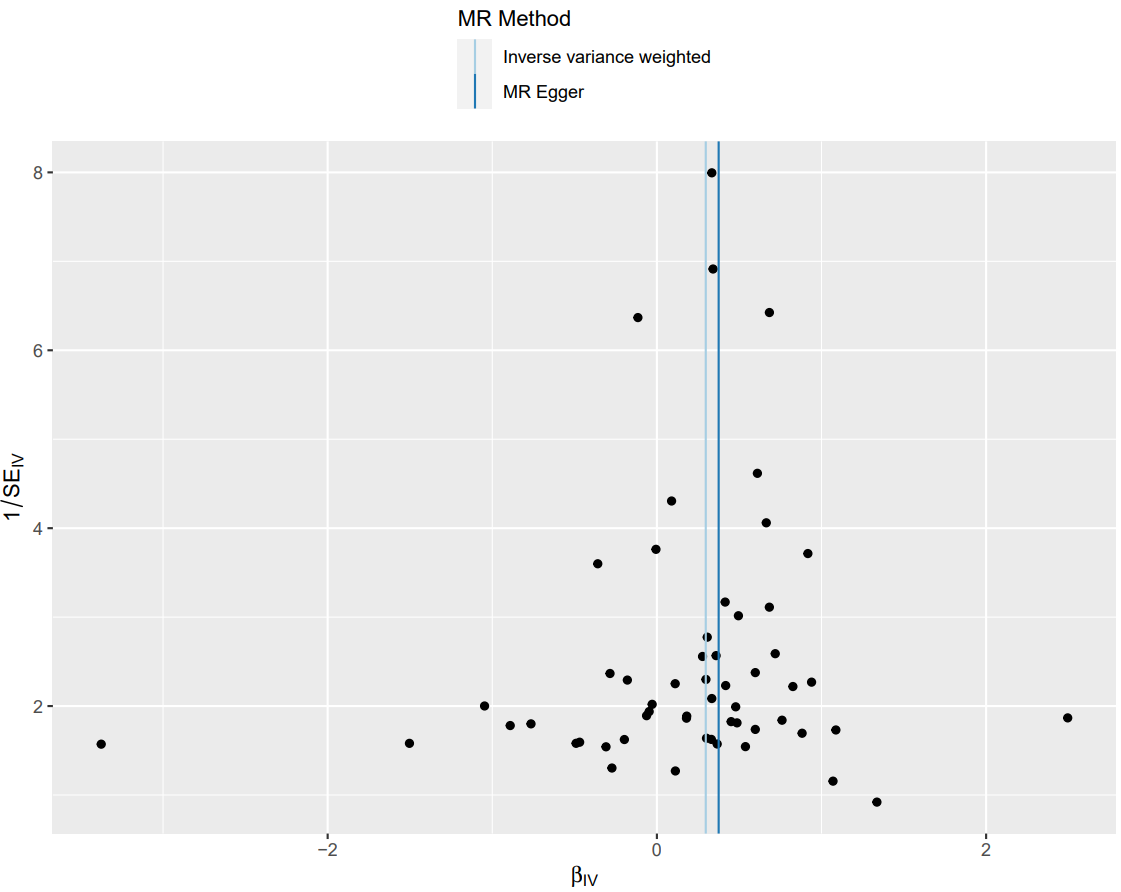

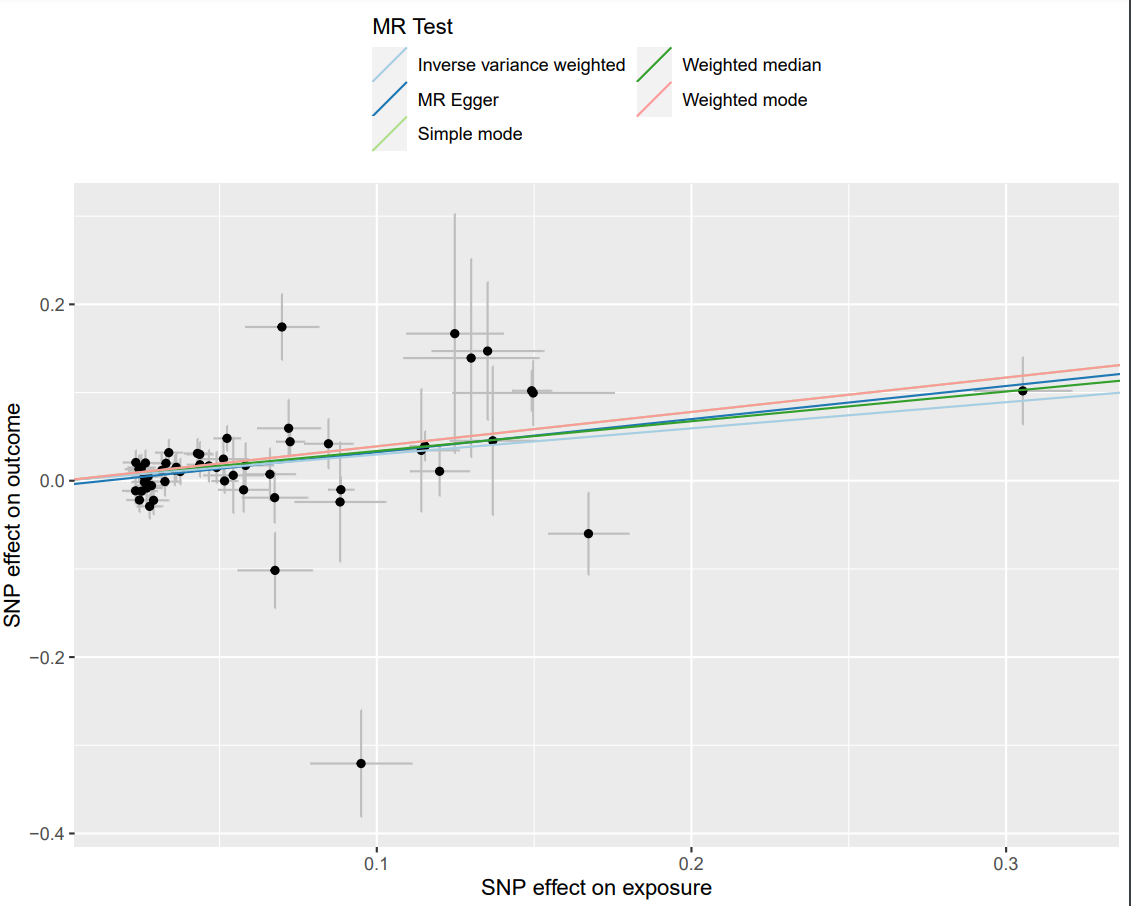
C D**

**Pulmonary embolism**

**
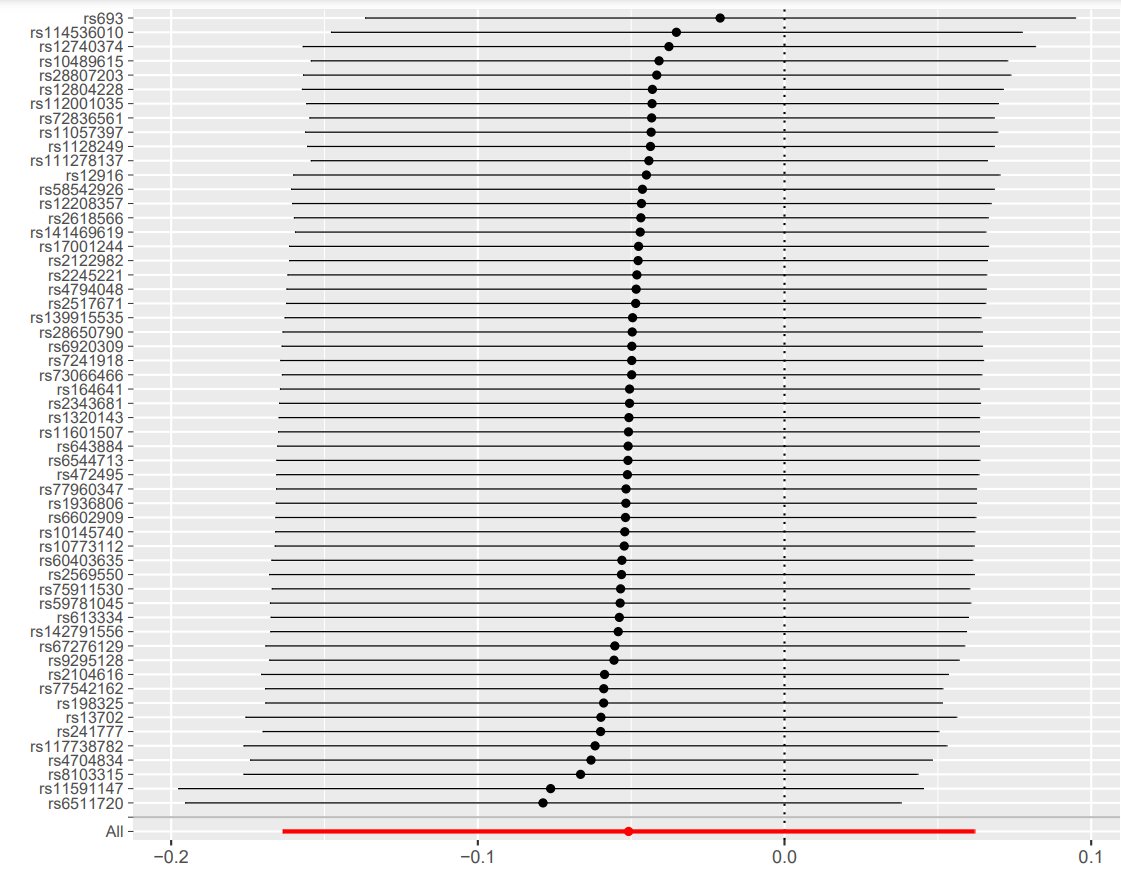

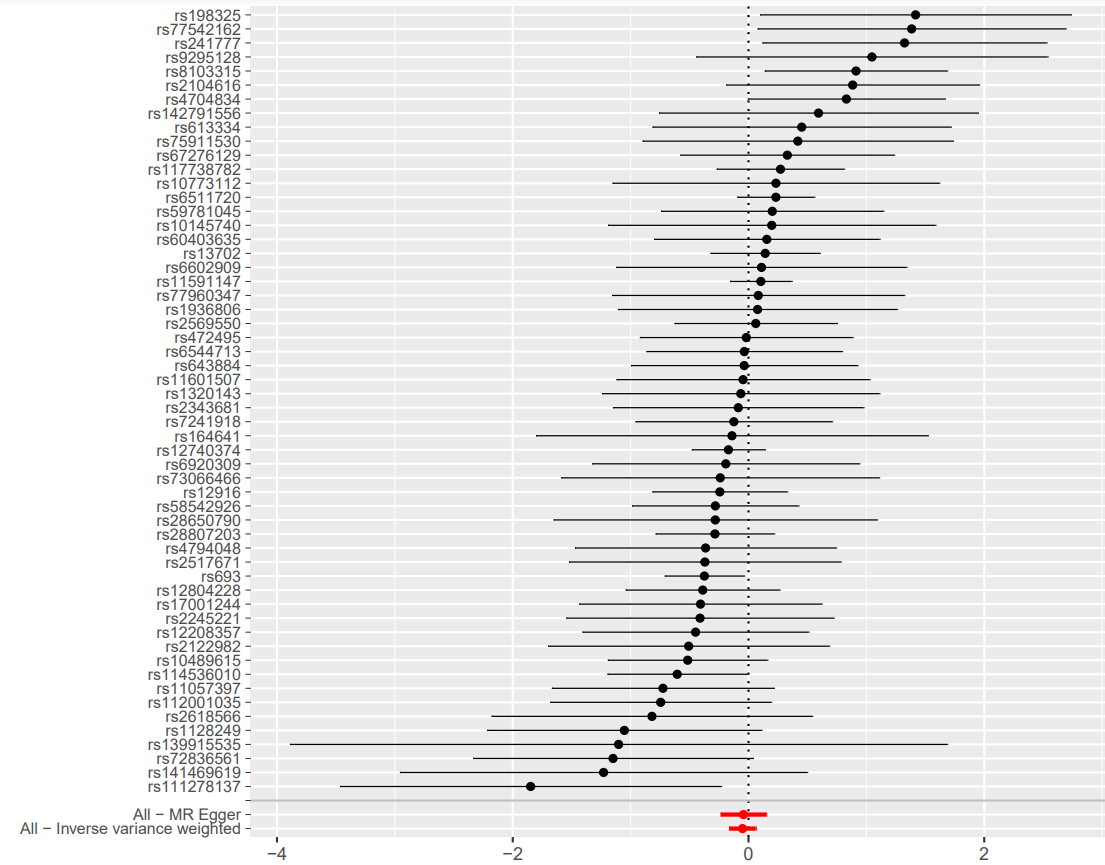
A B**

**
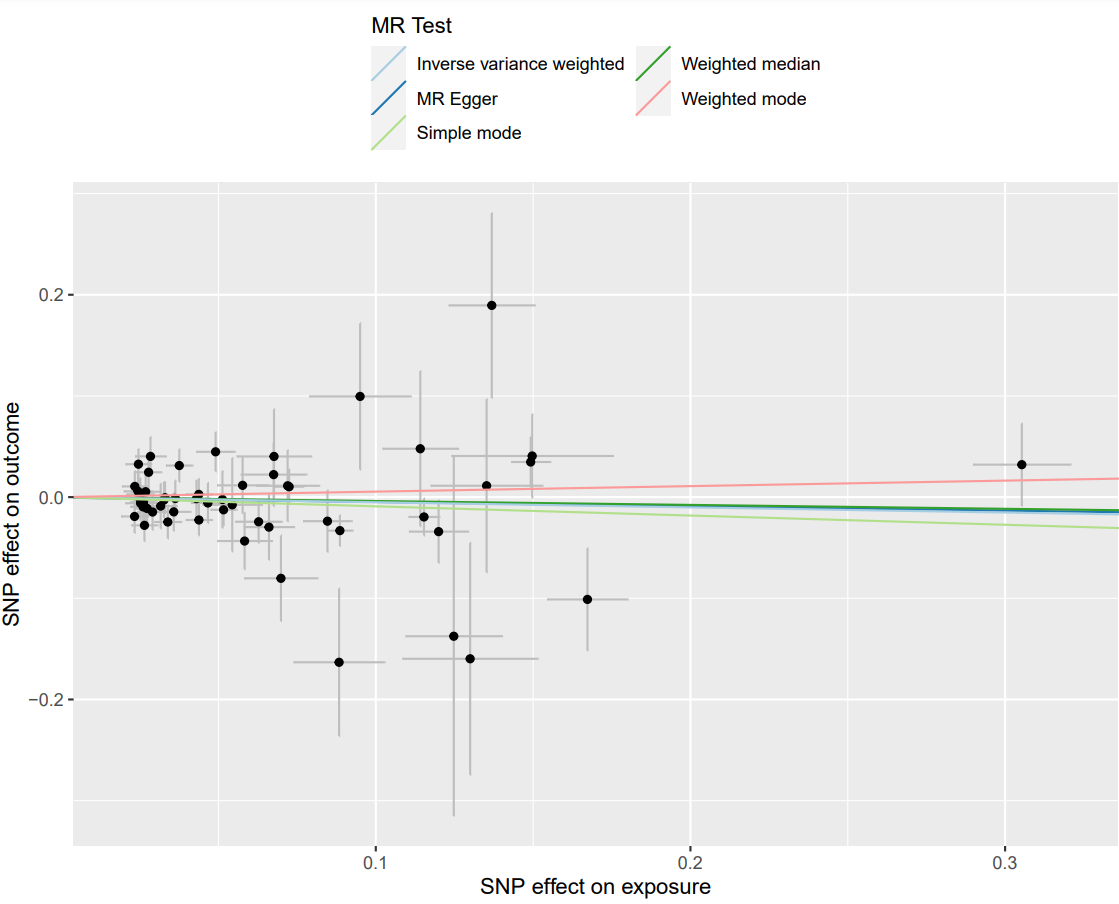

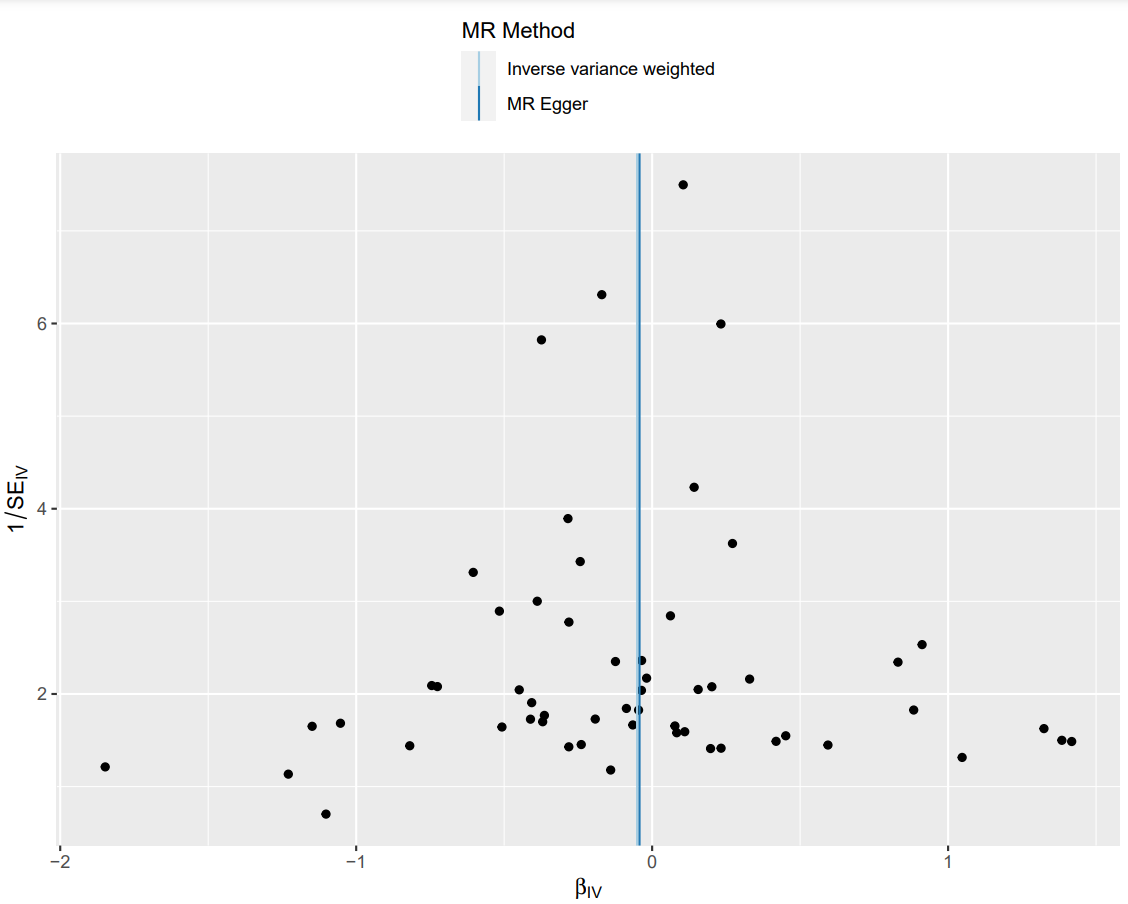
C D**

**Cardiomyopathy**

**
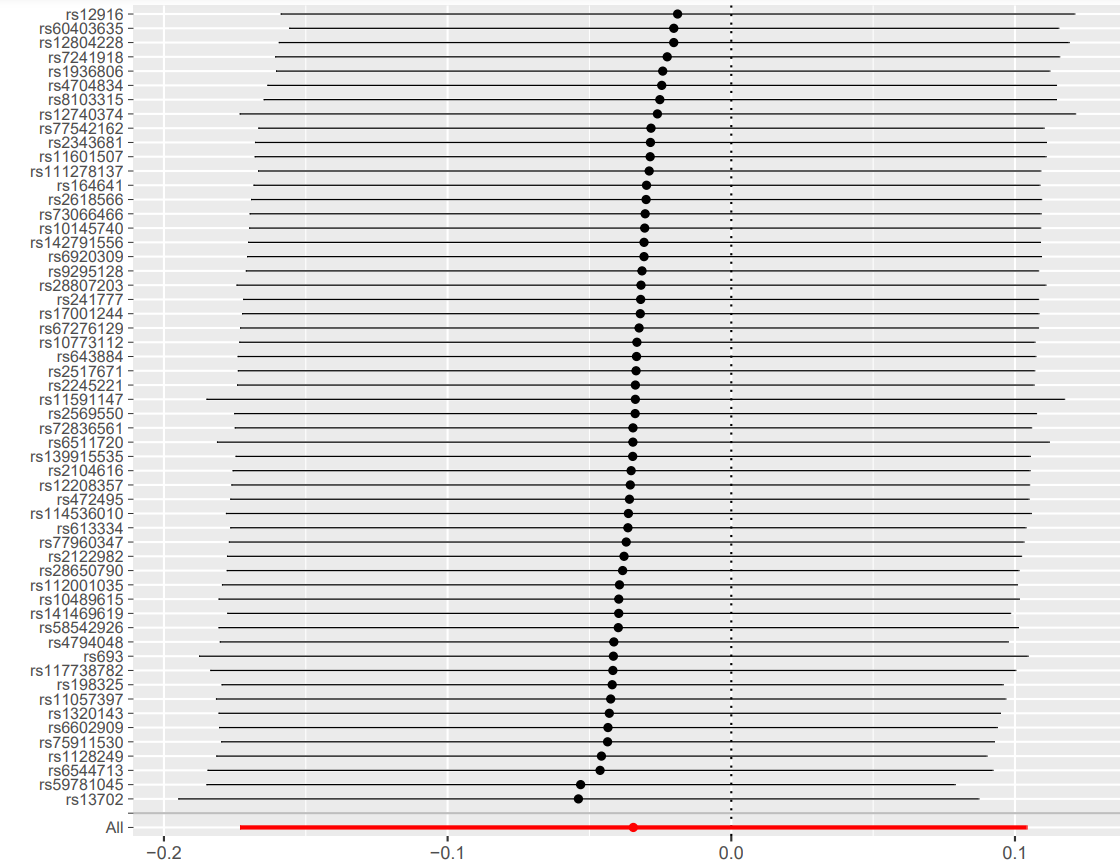

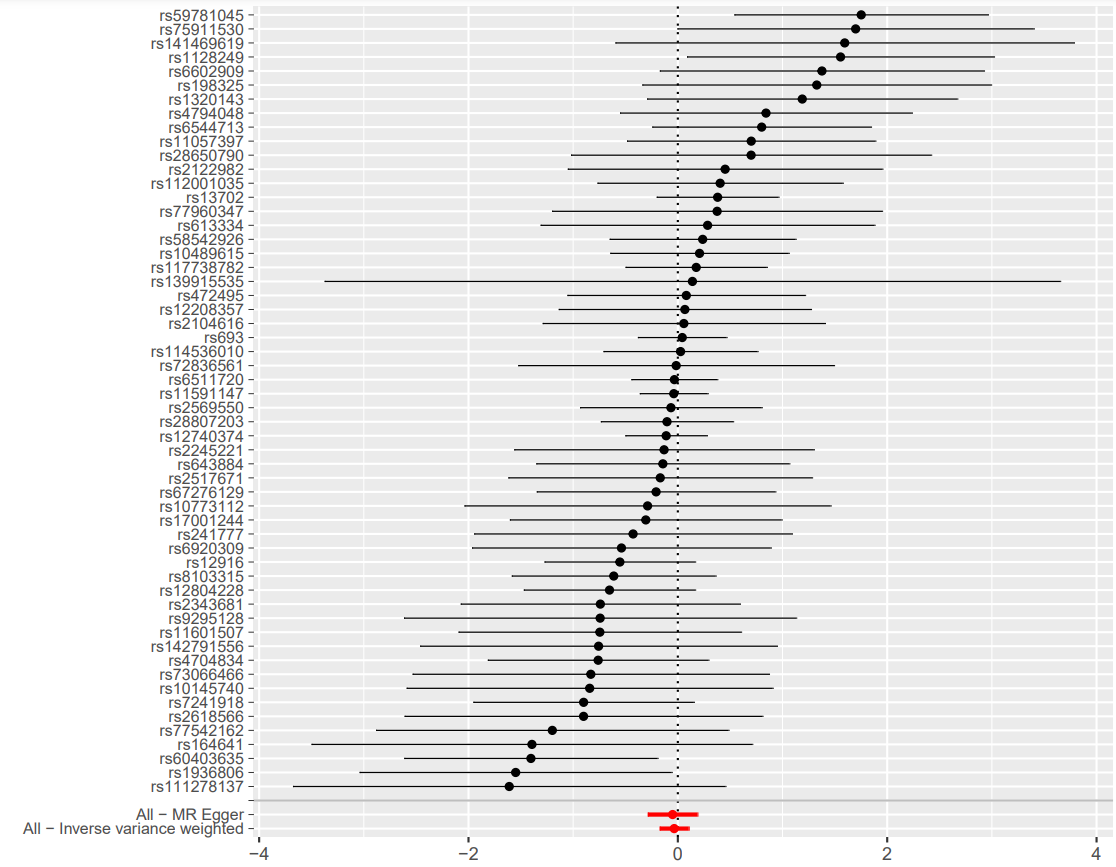
A B**

**
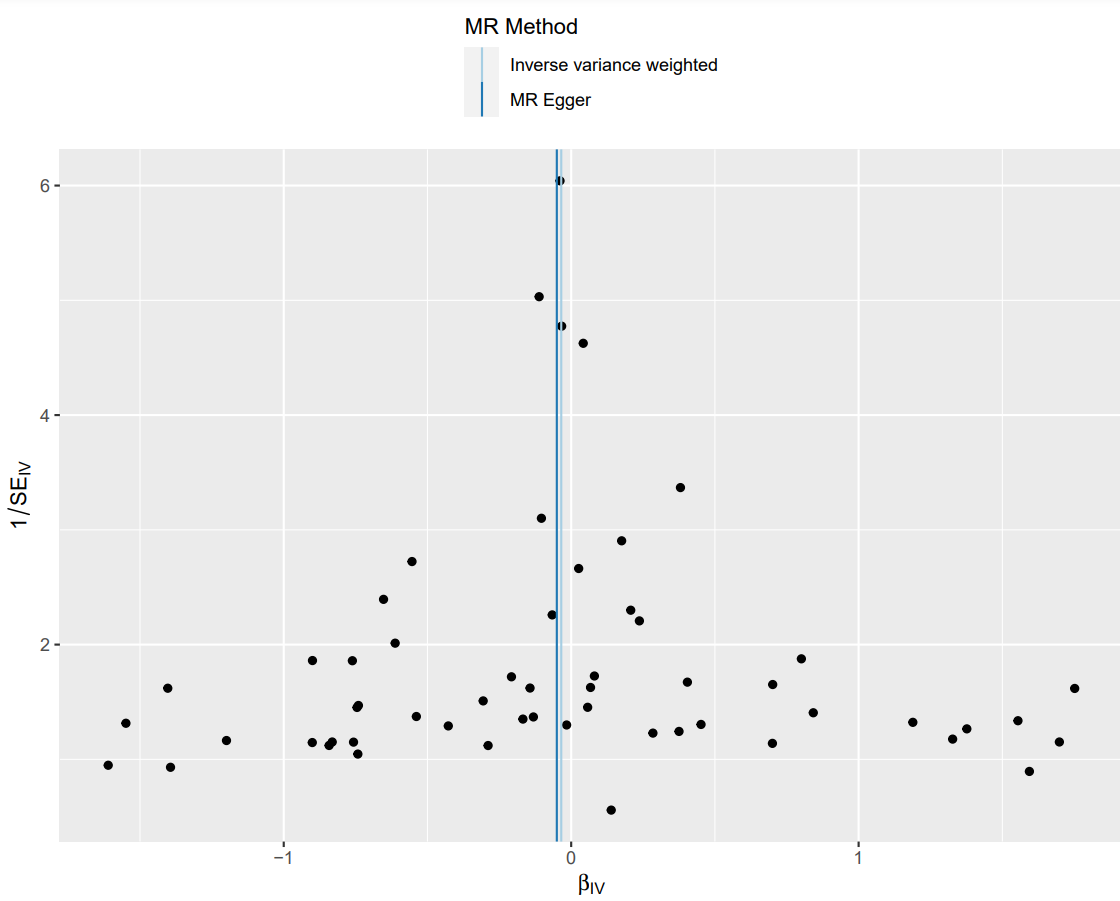
C D**

**
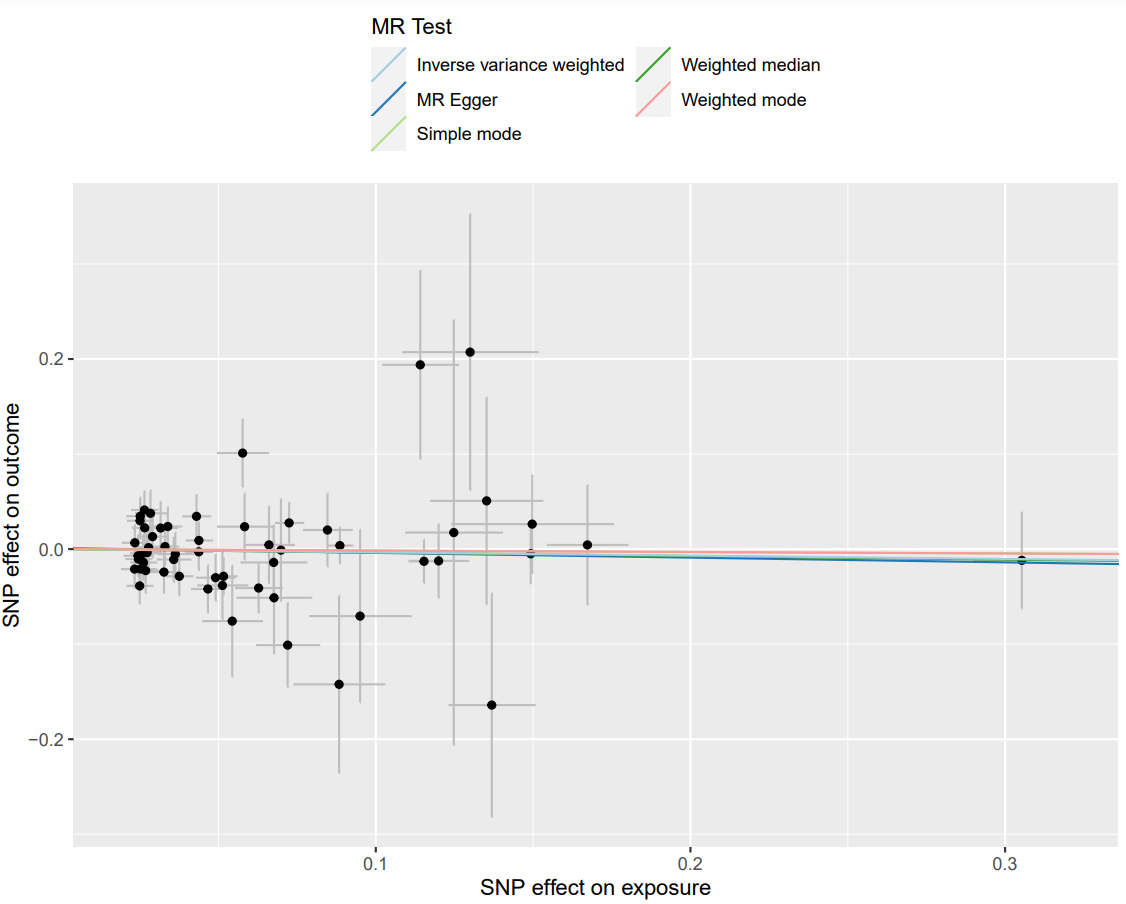
**

**Intracerebral hemorrhage**

**
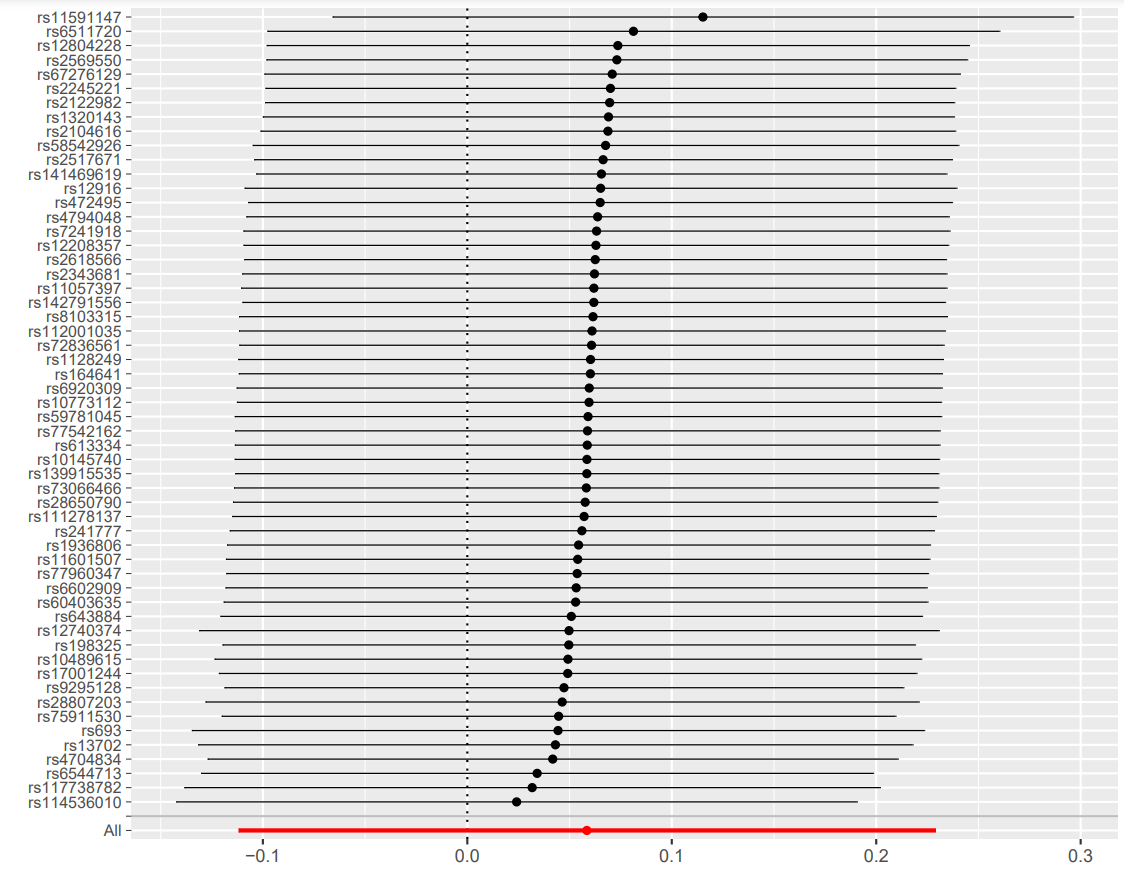

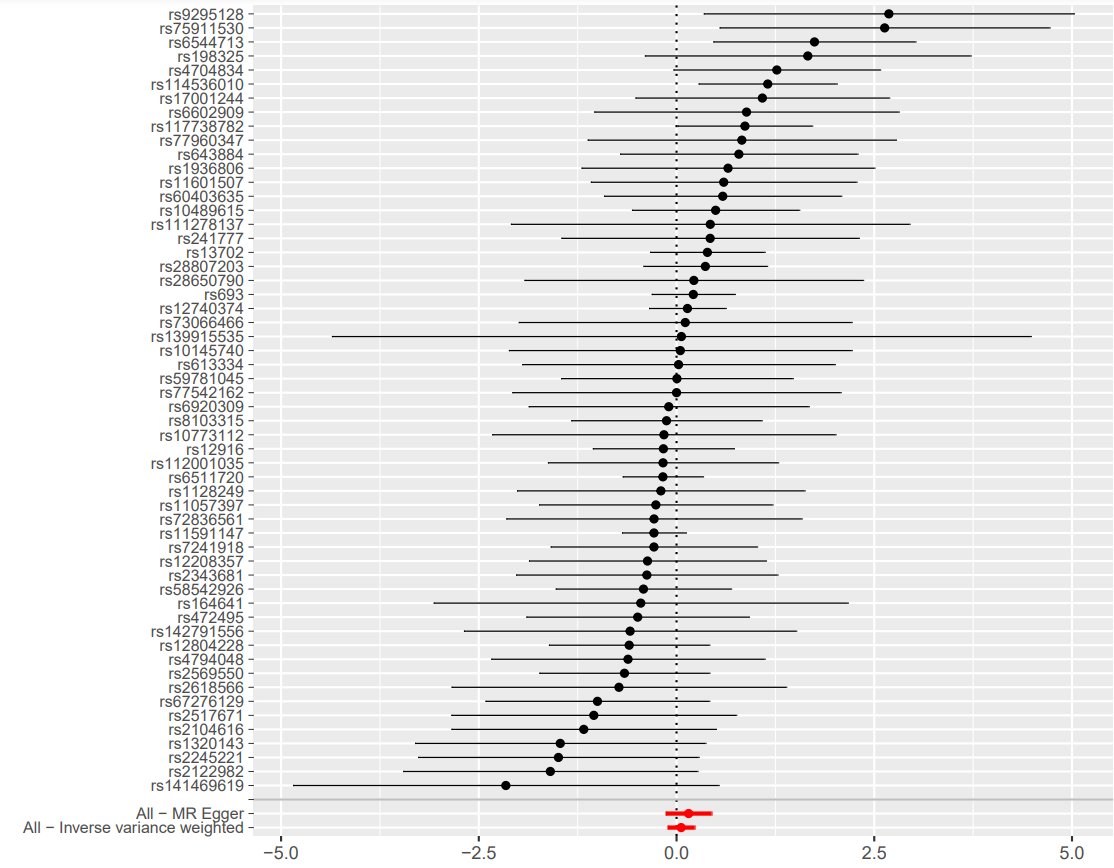
A B**

**
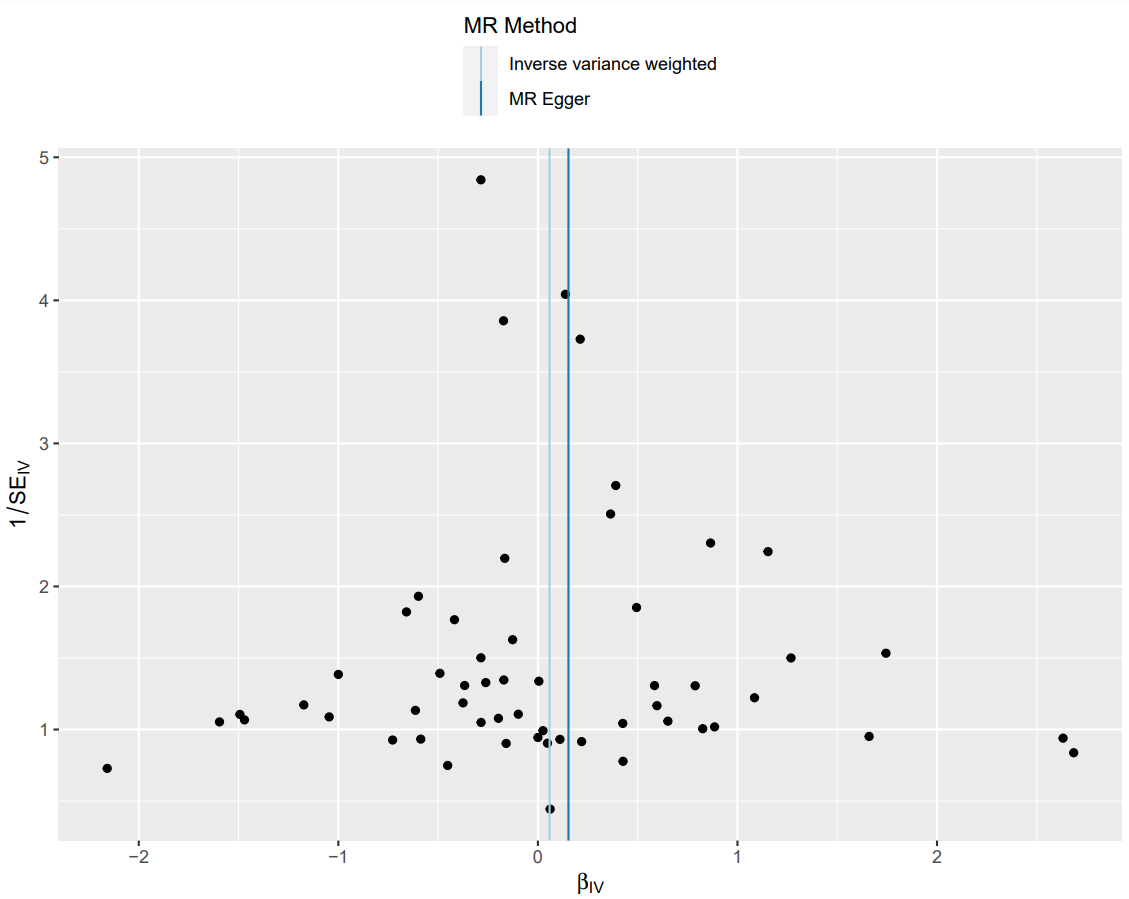
**

**
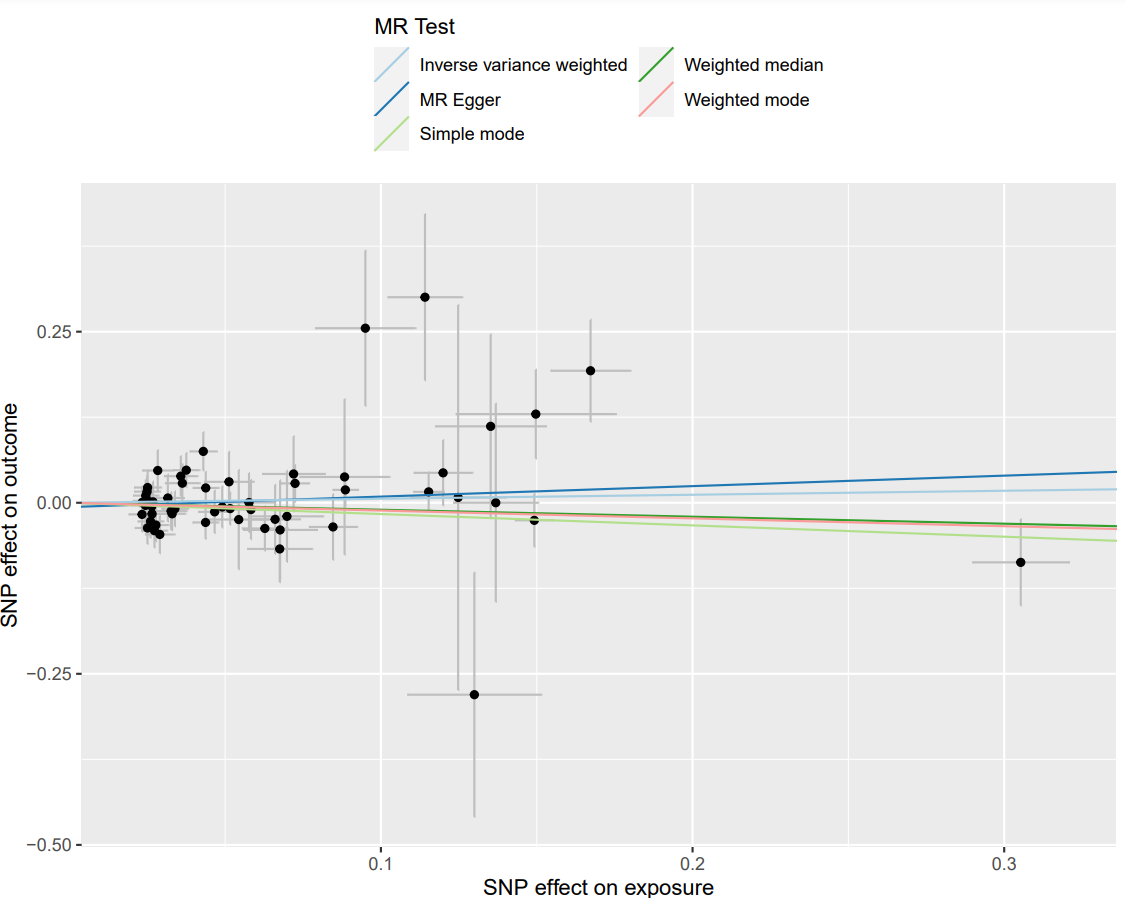
C D**

**Subarachnoid hemorrhage**

**
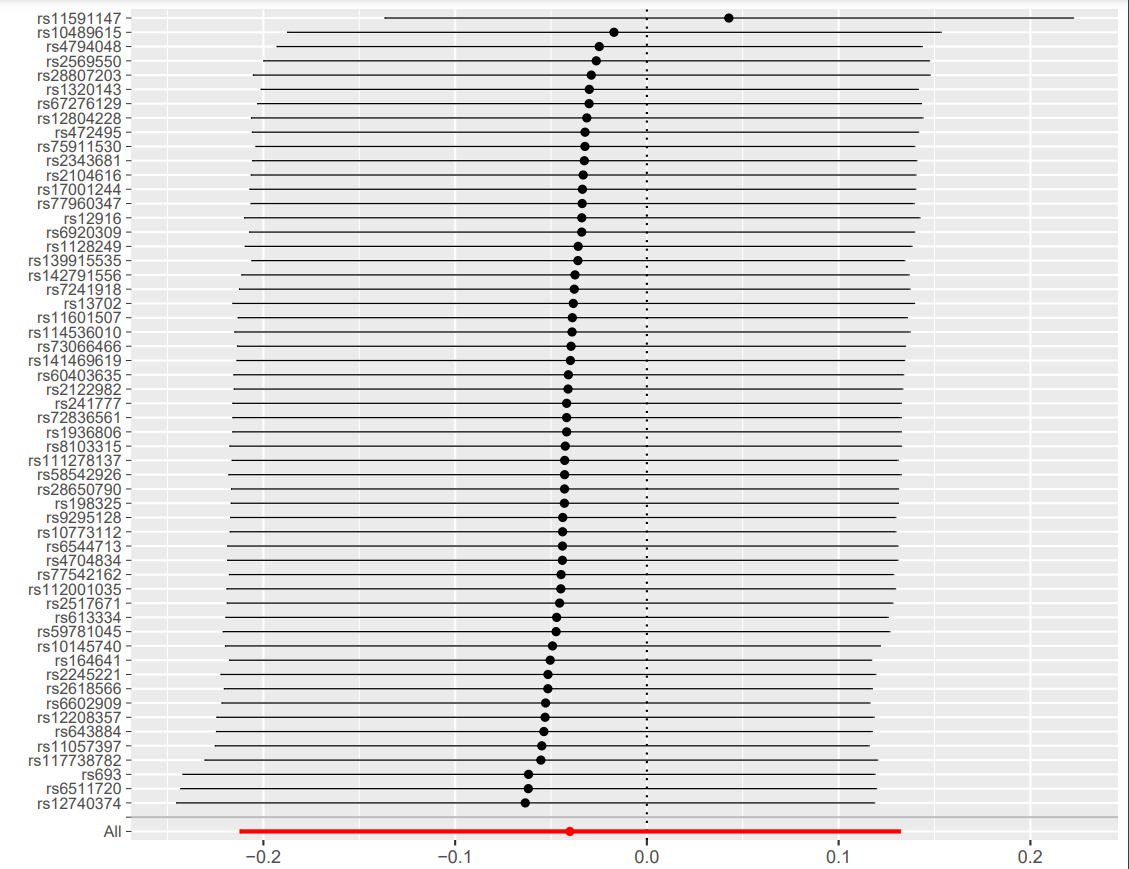

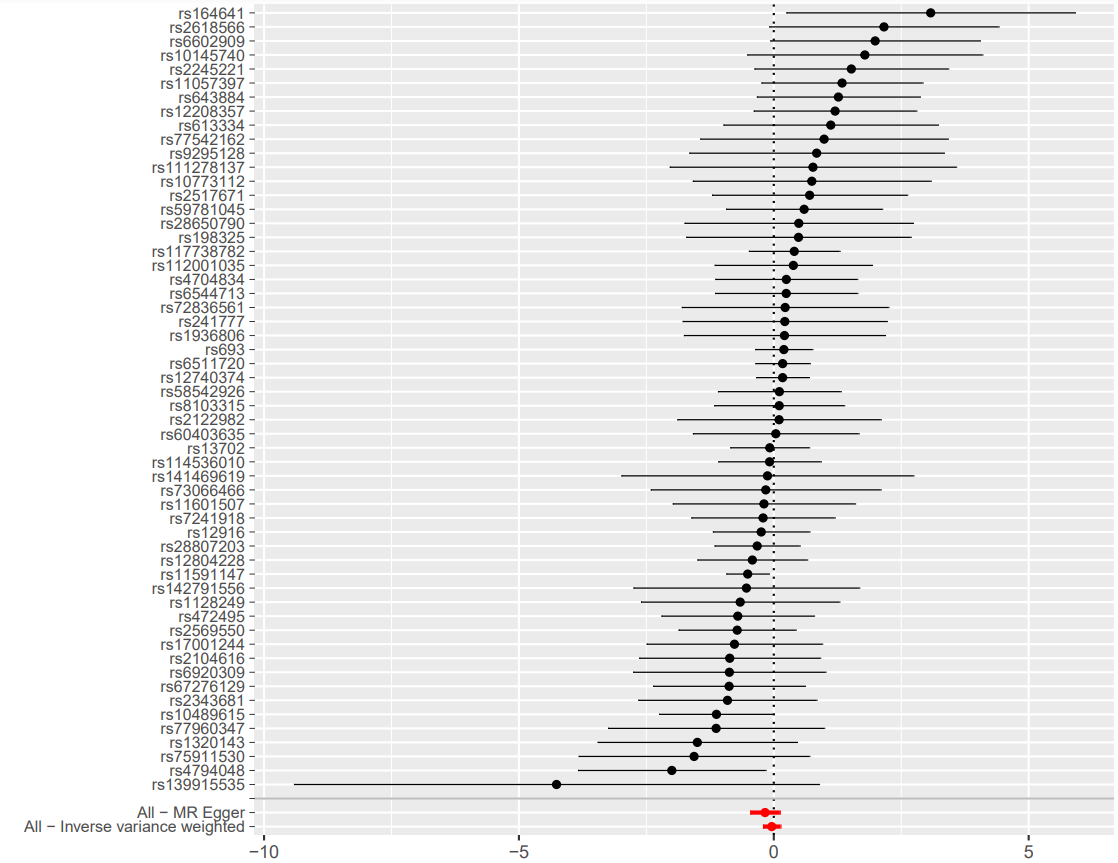
A B**

**
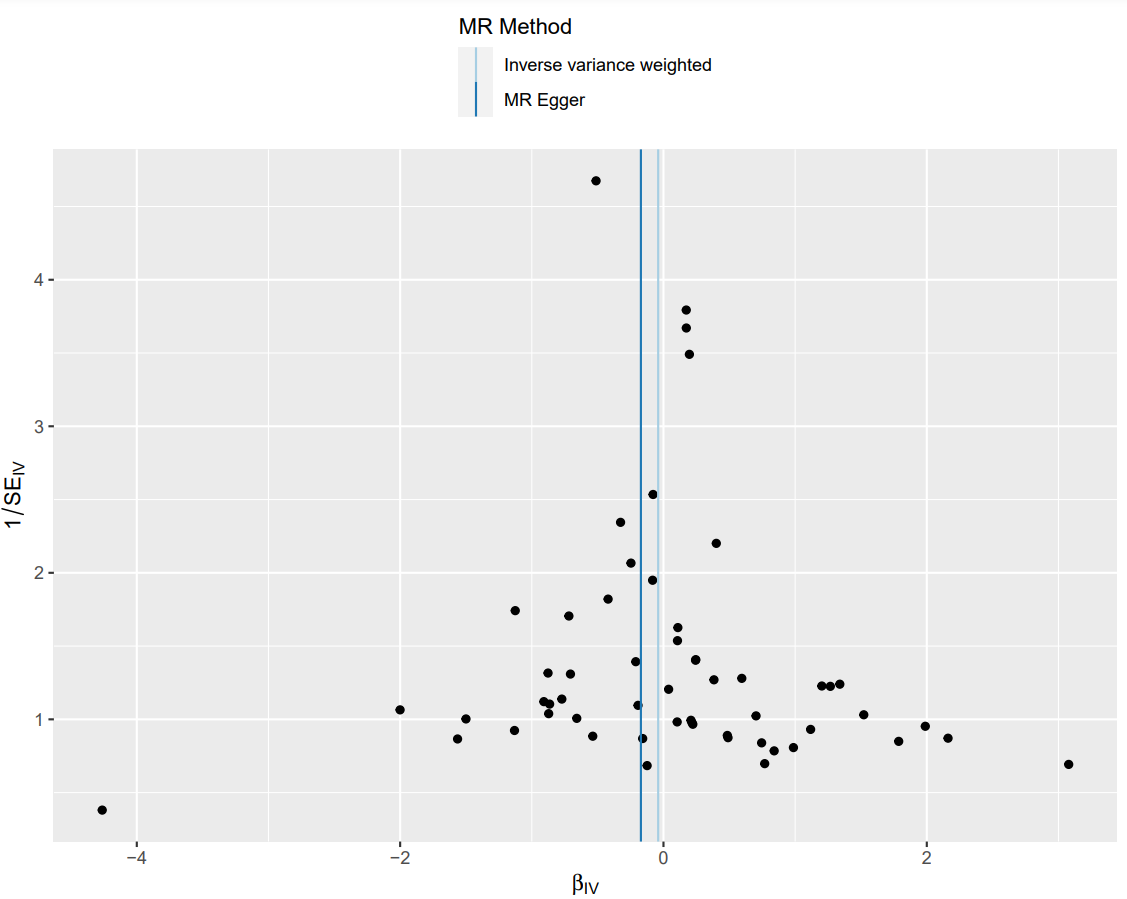

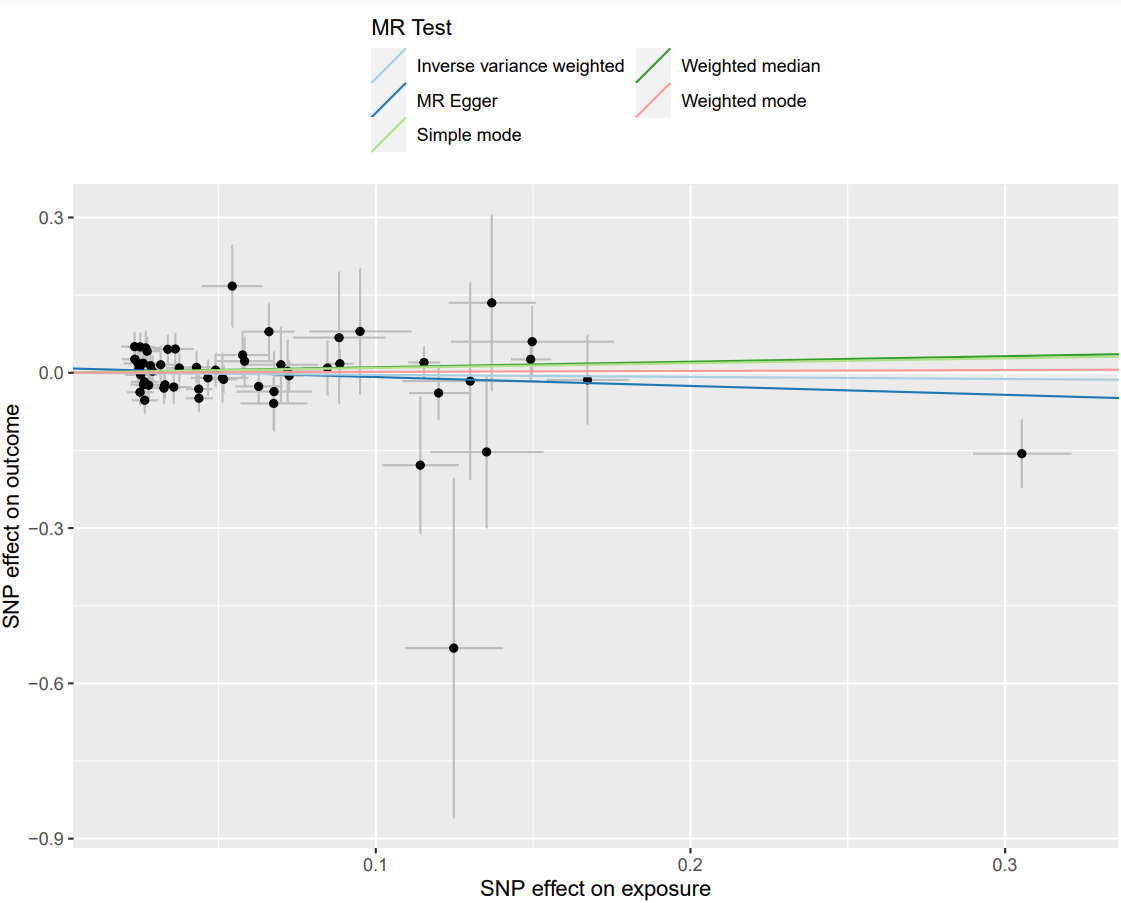
C D**

**Ischemic stroke**

**
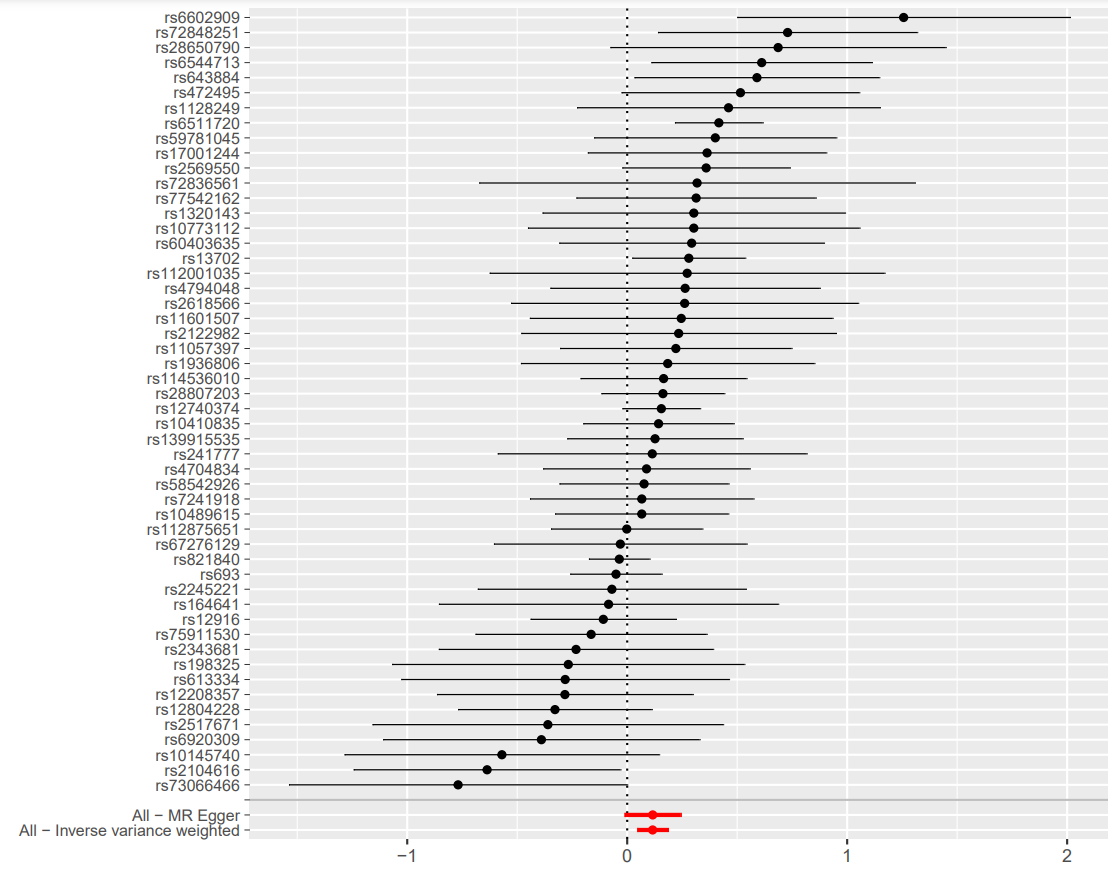

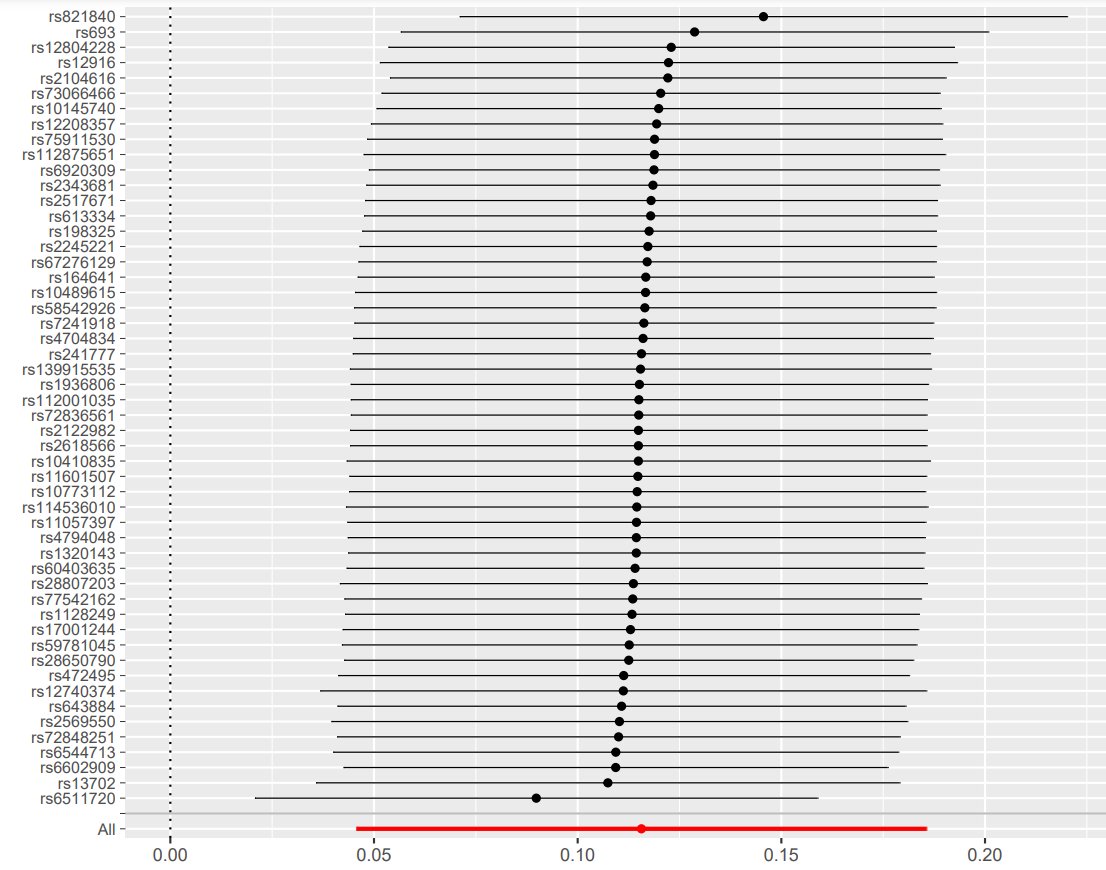
A B**

**
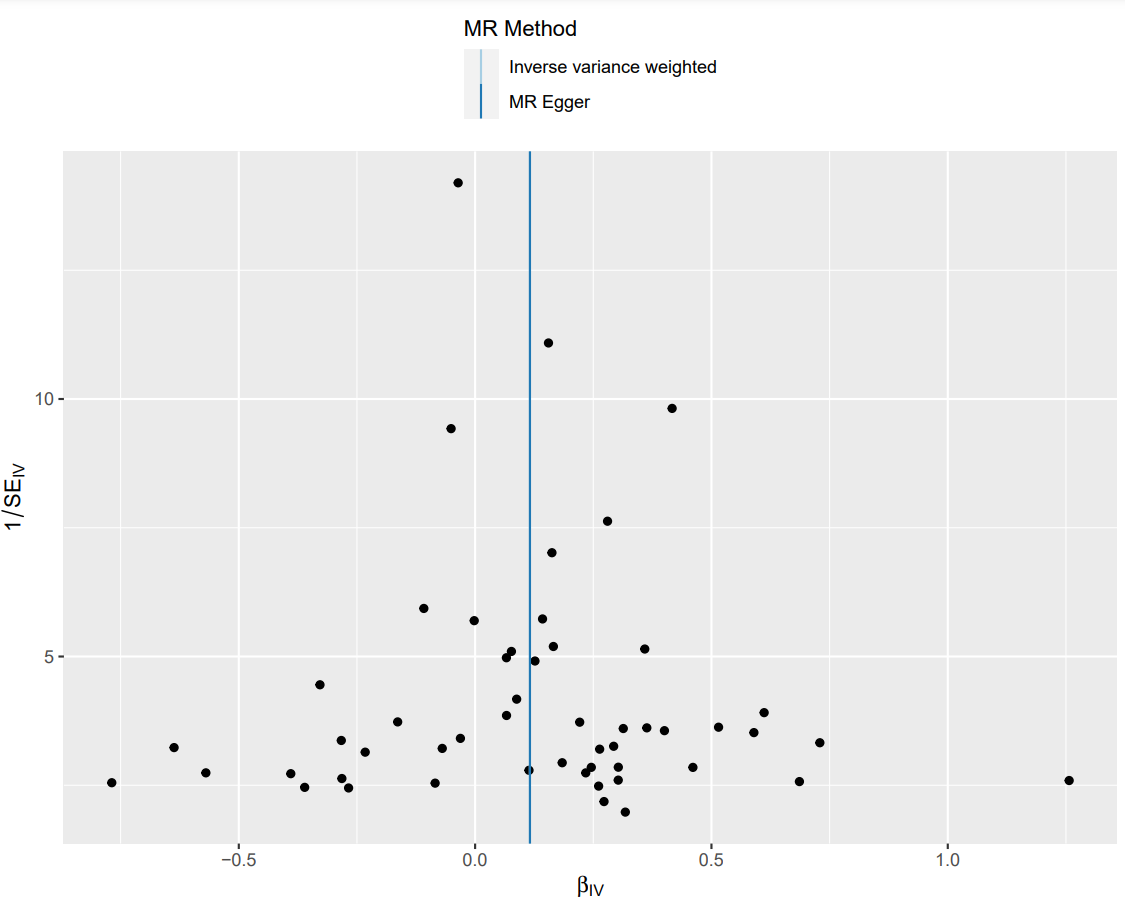

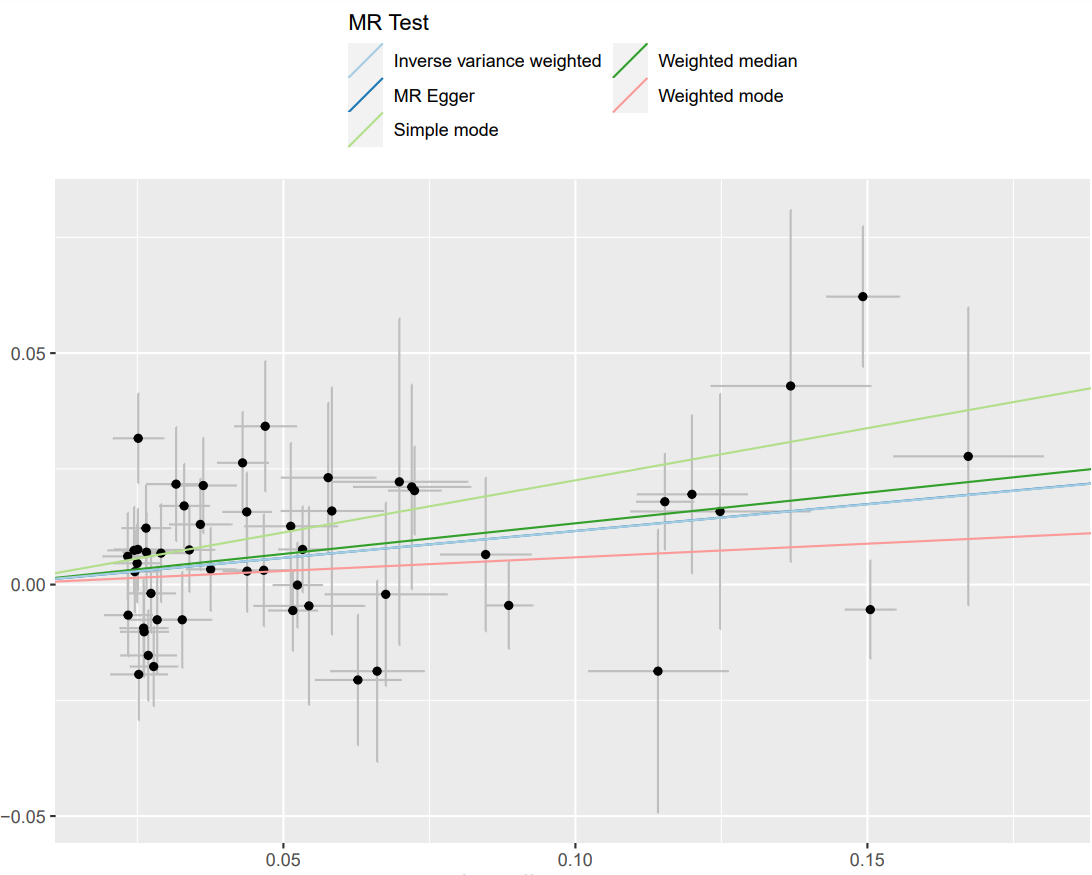
C D**

**Transient ischemic attack**

**
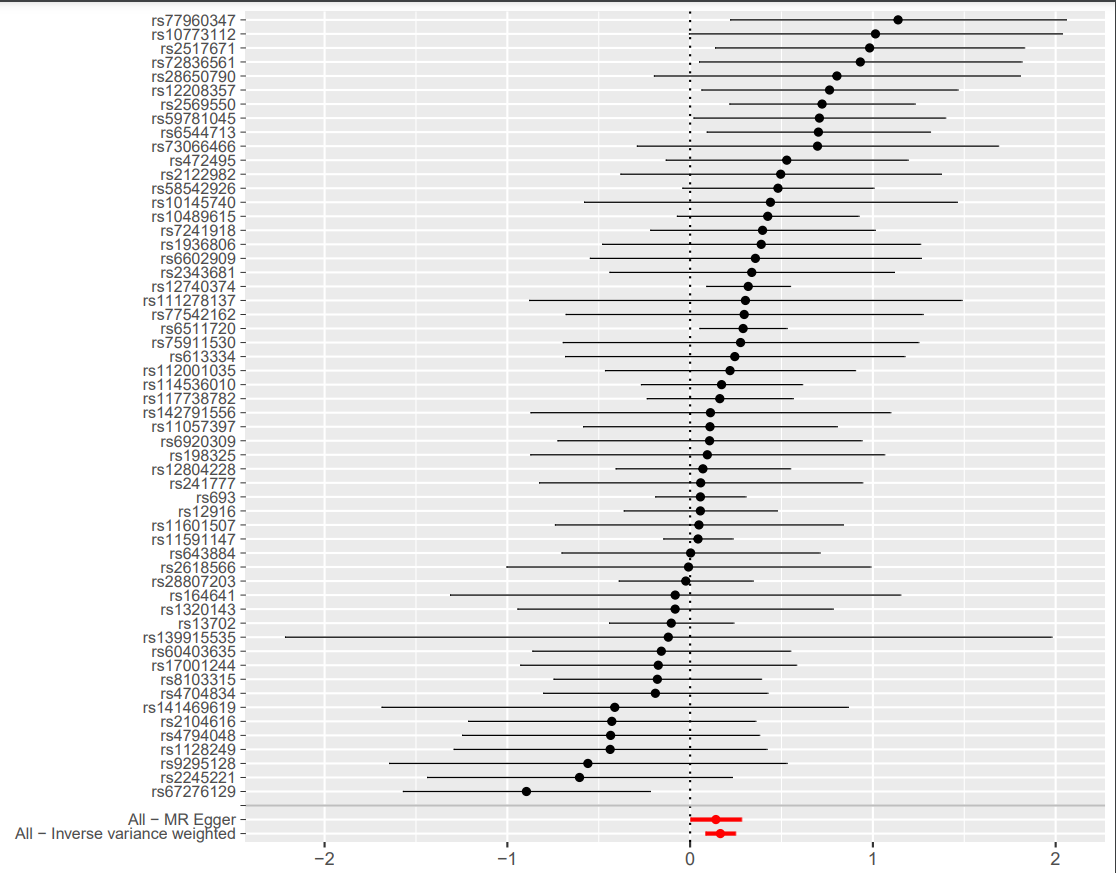

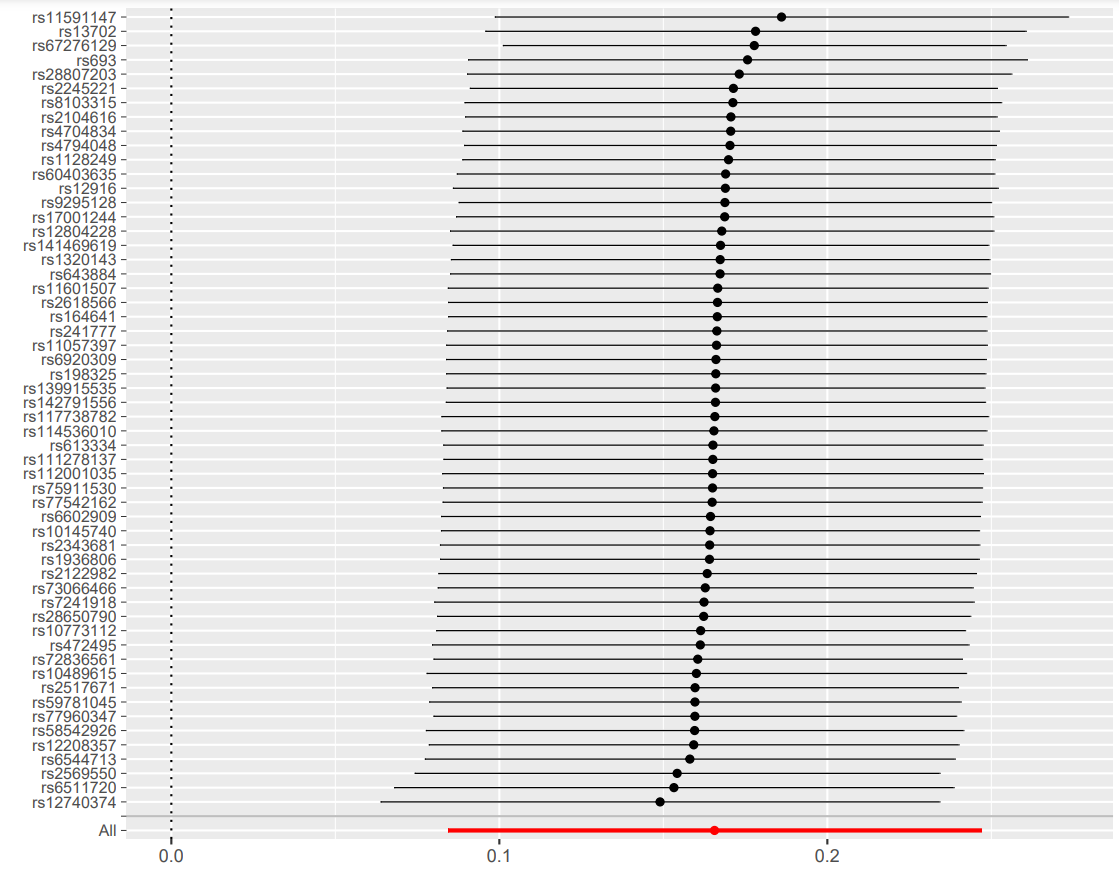
A B**

**
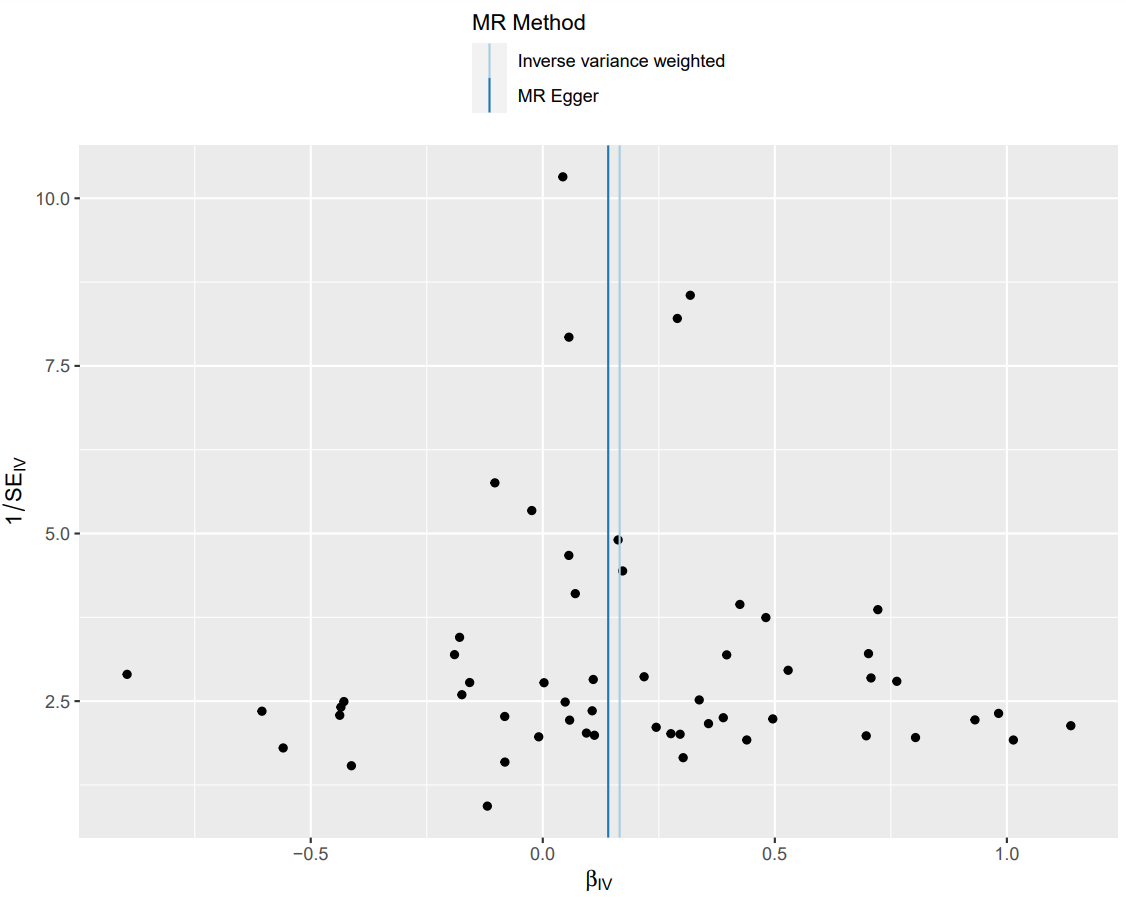

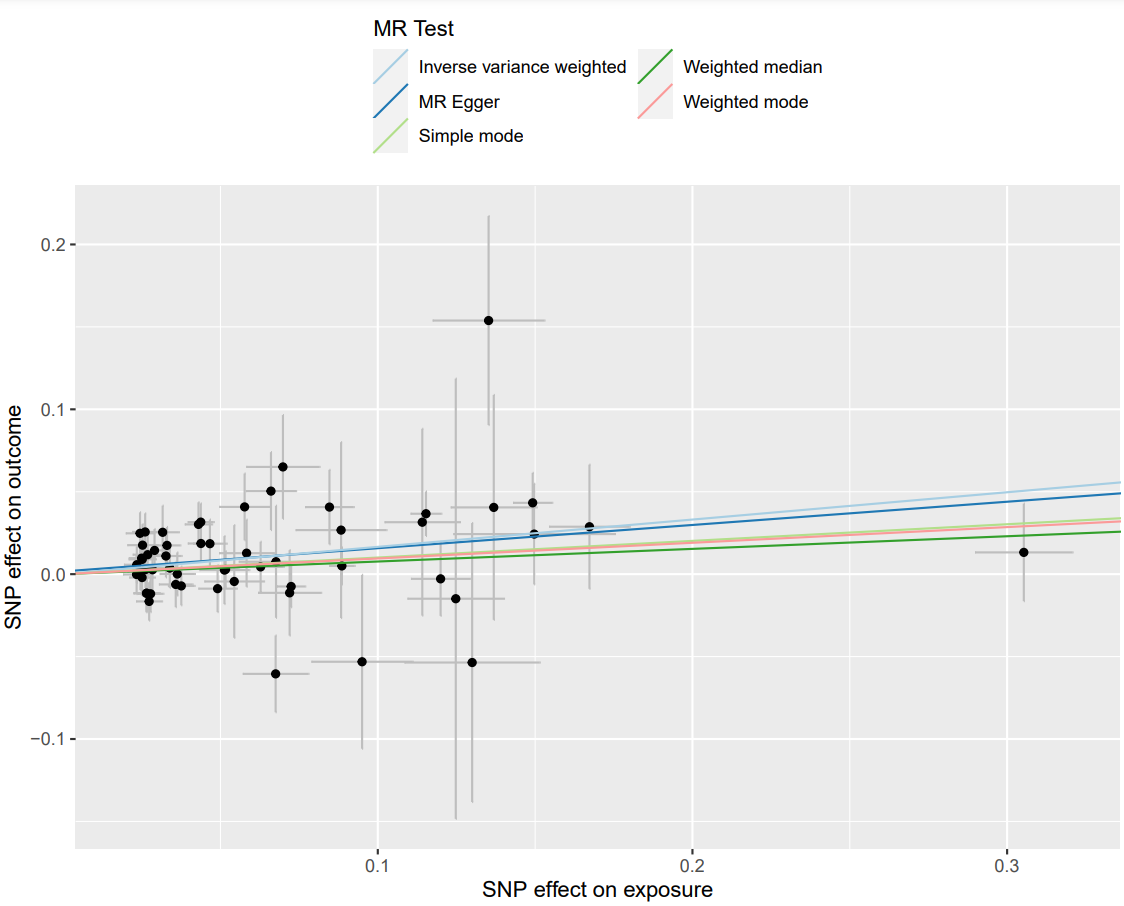
C D**

**DVT of lower extremities**

**
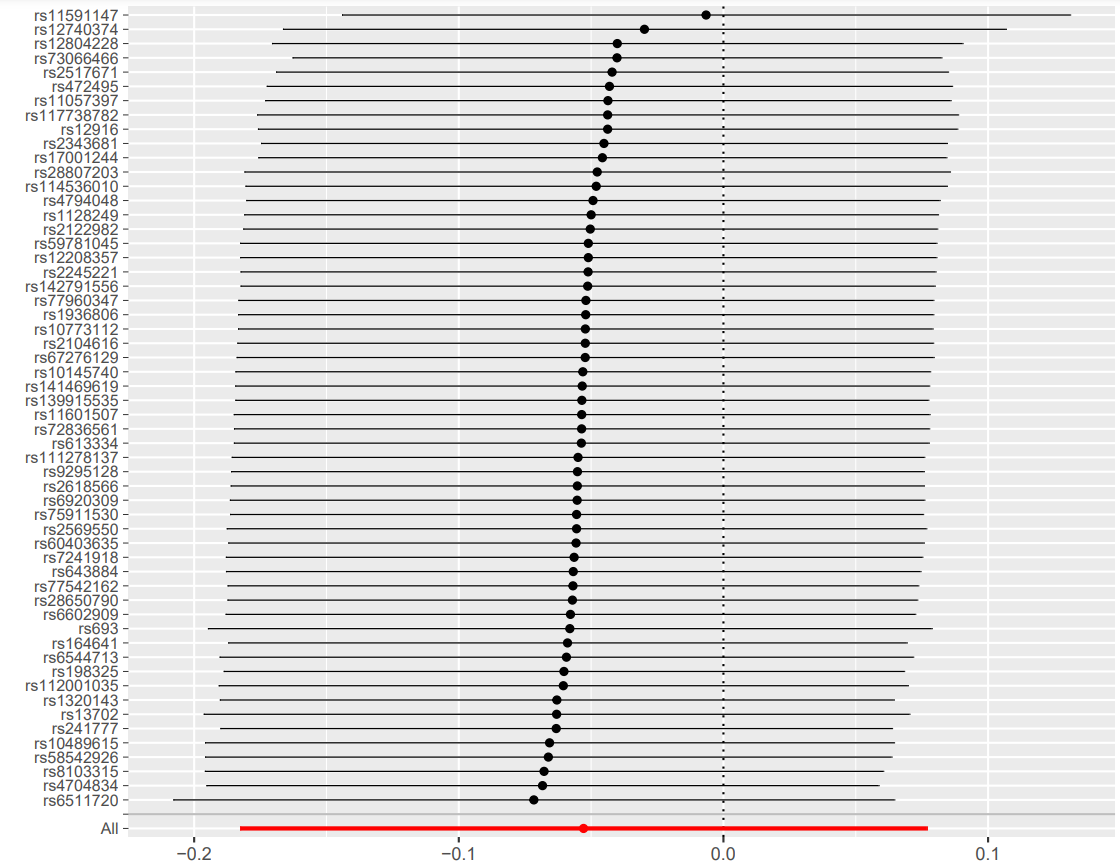

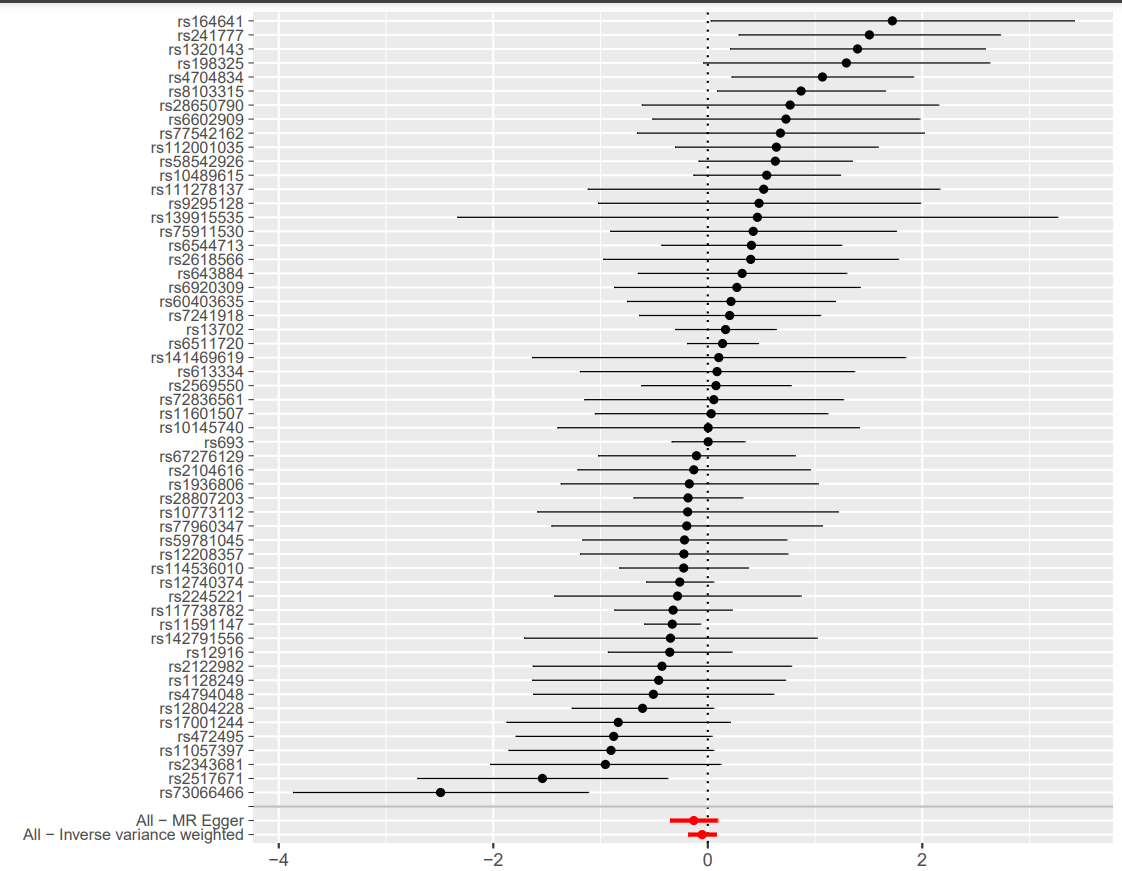
A B**

**
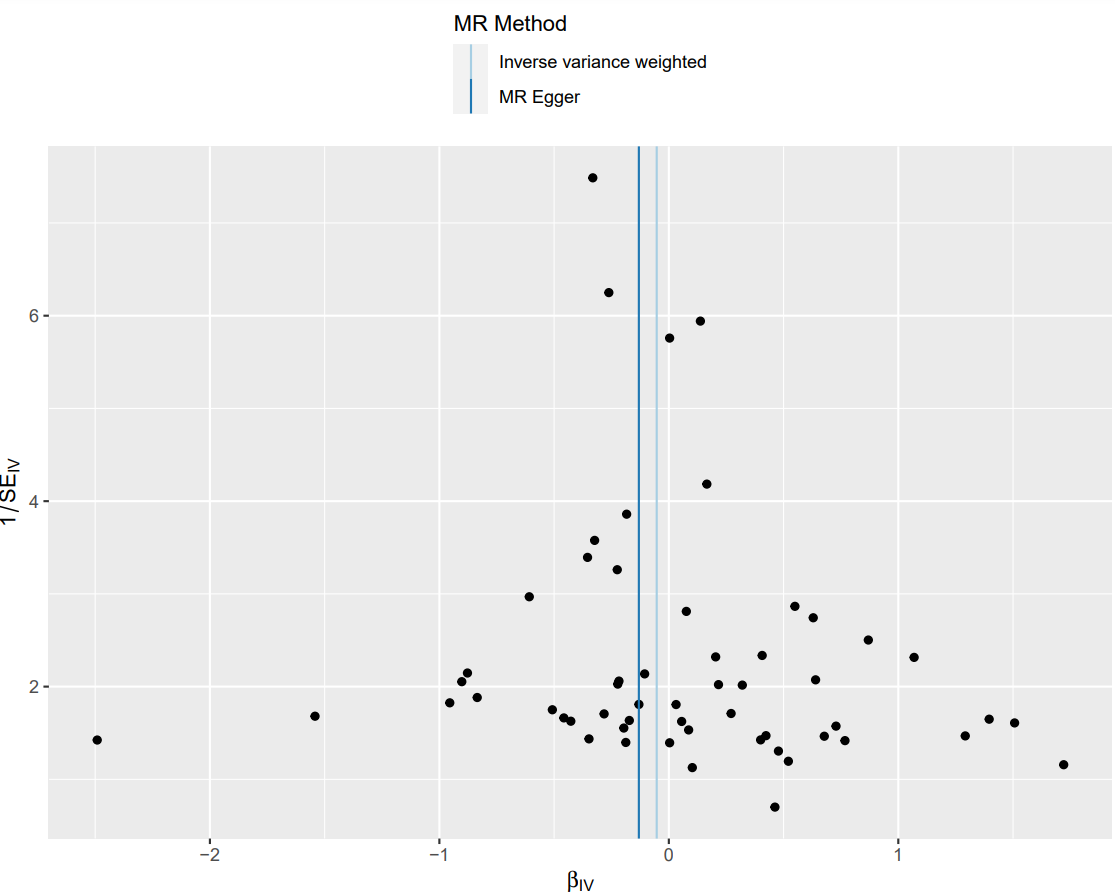

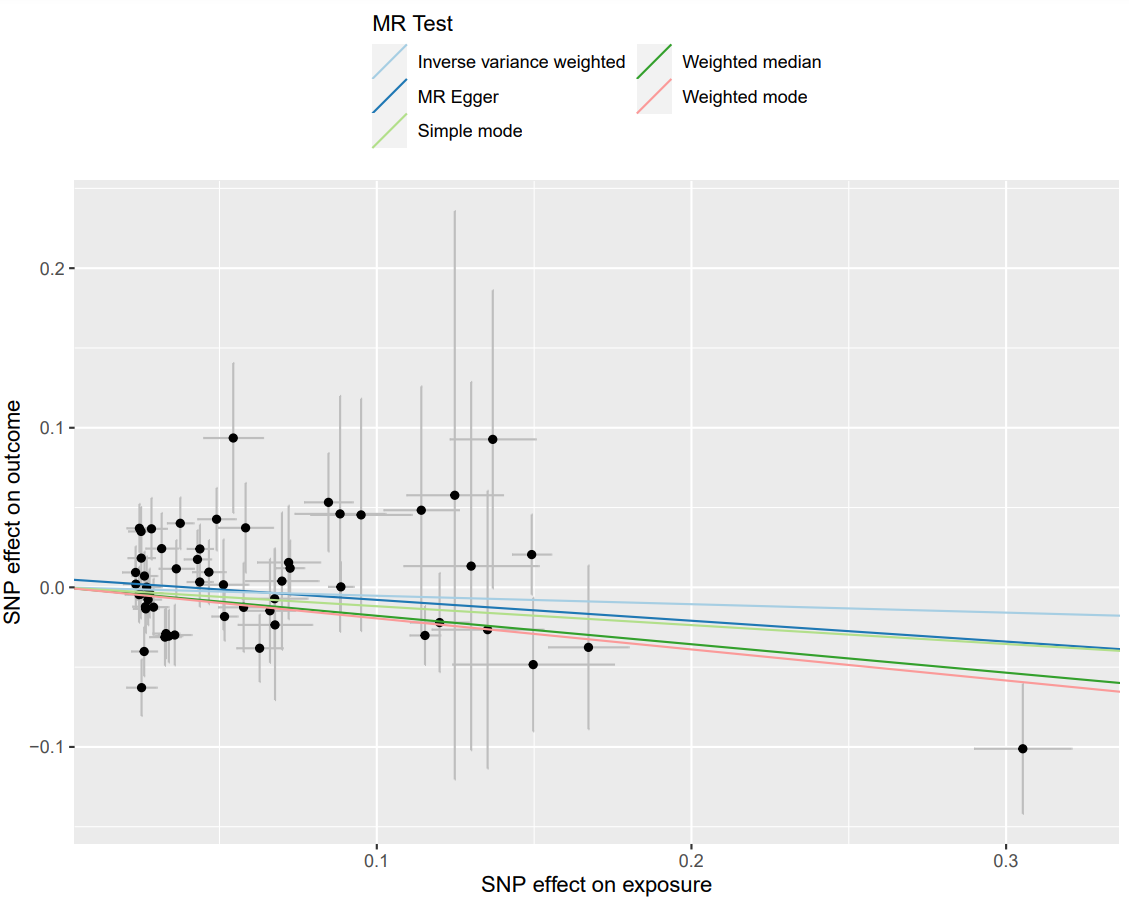
C D**

**Type 2 diabetes**

**
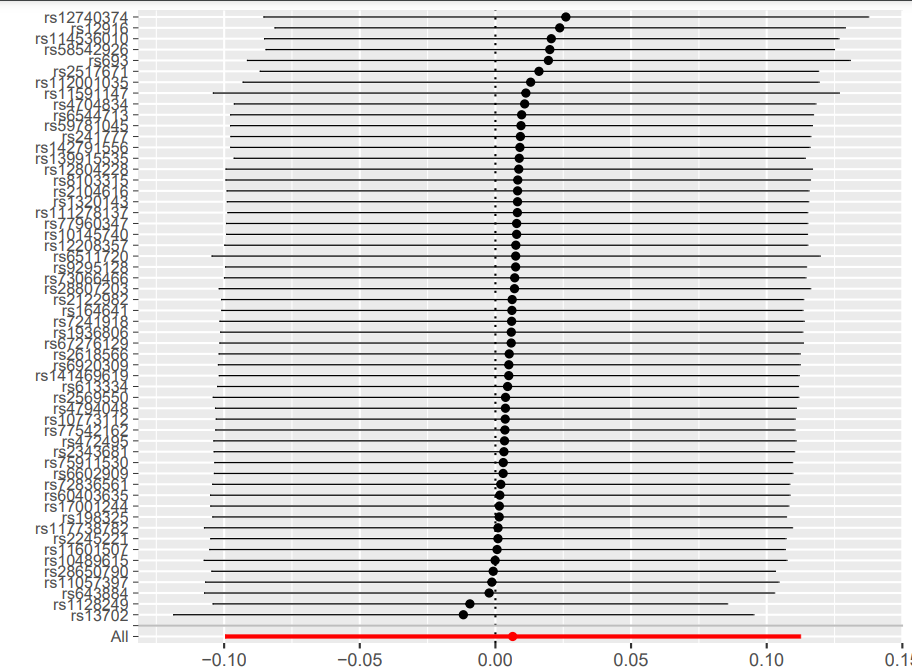

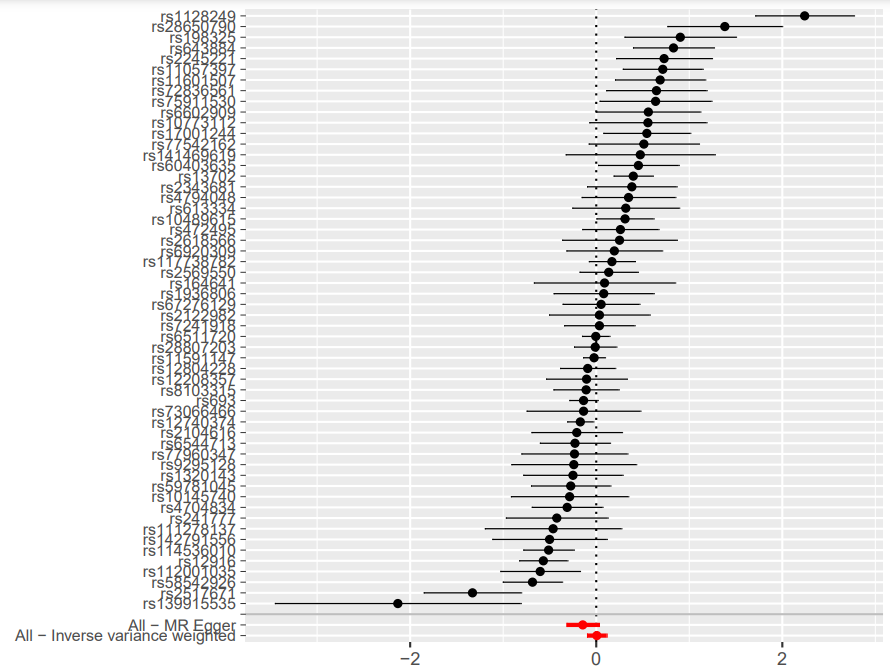
A B**

**
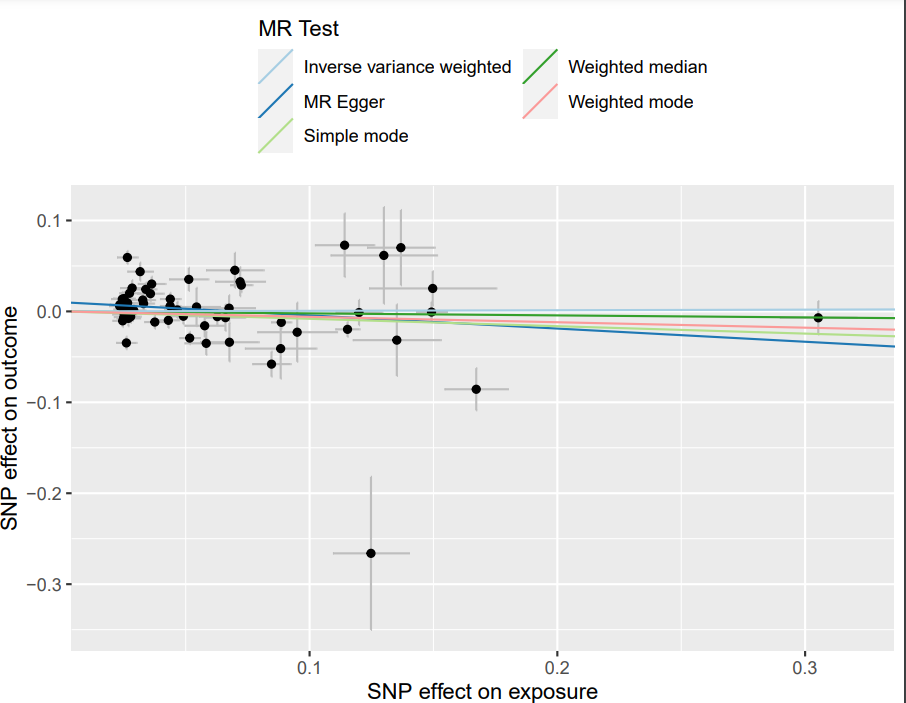

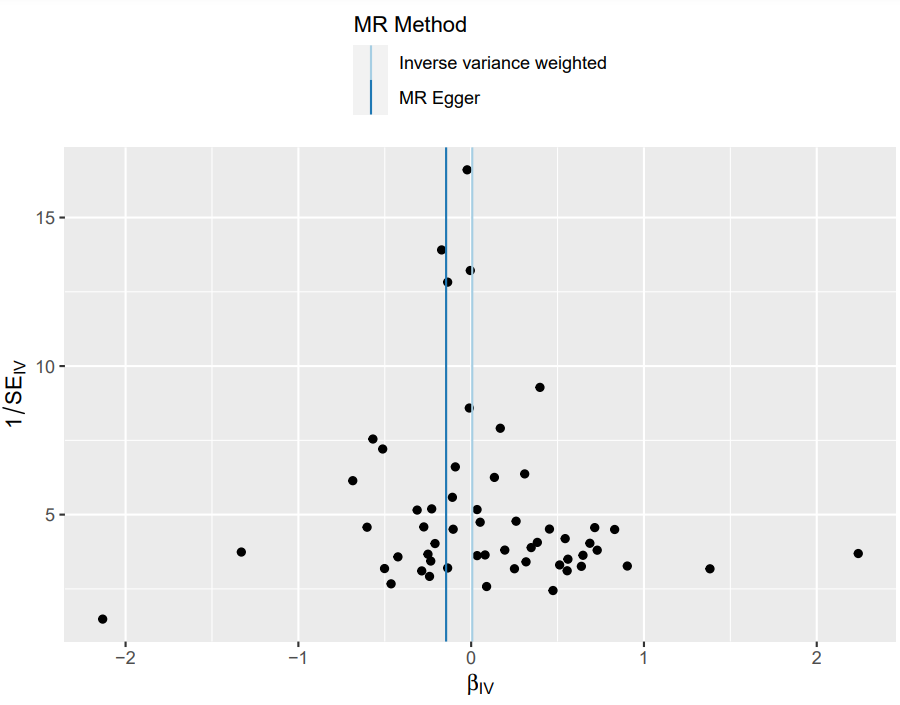
C D**

**Obesity**

**
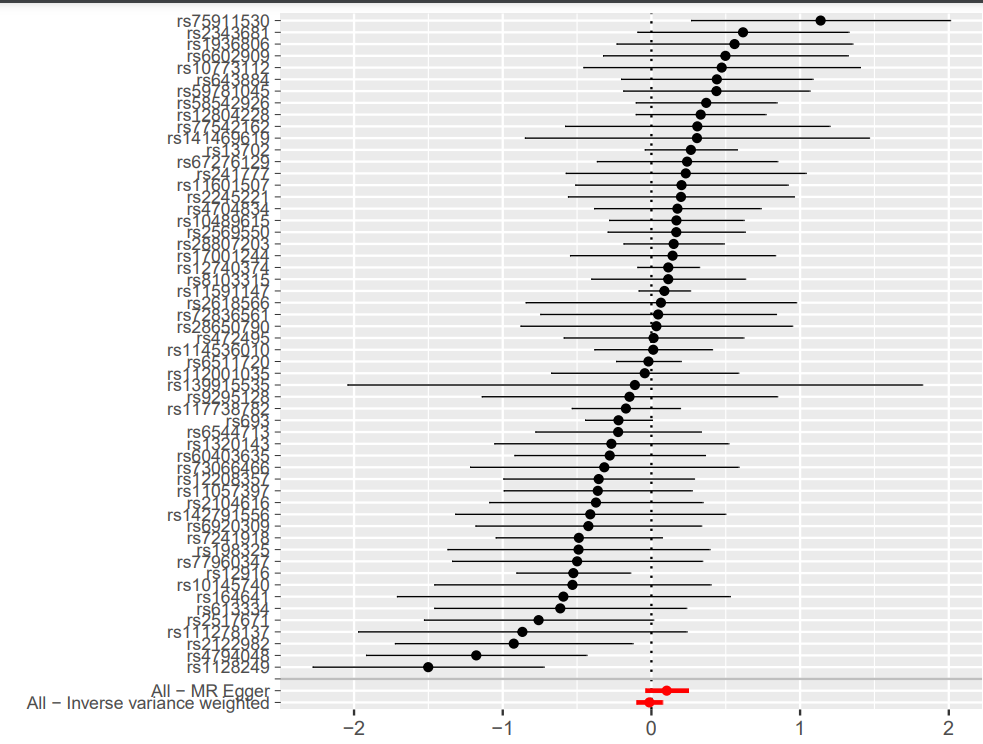

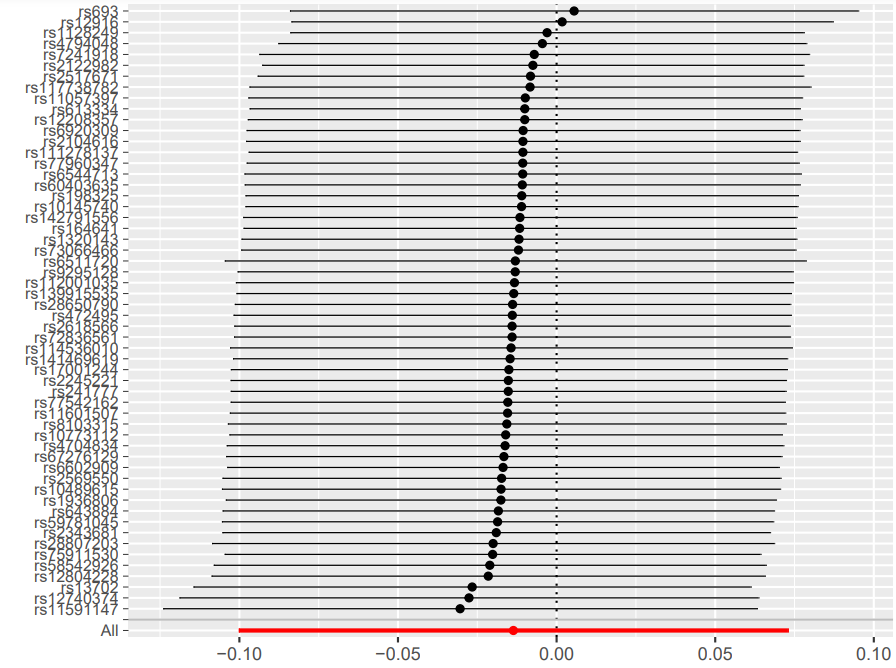
A B**

**
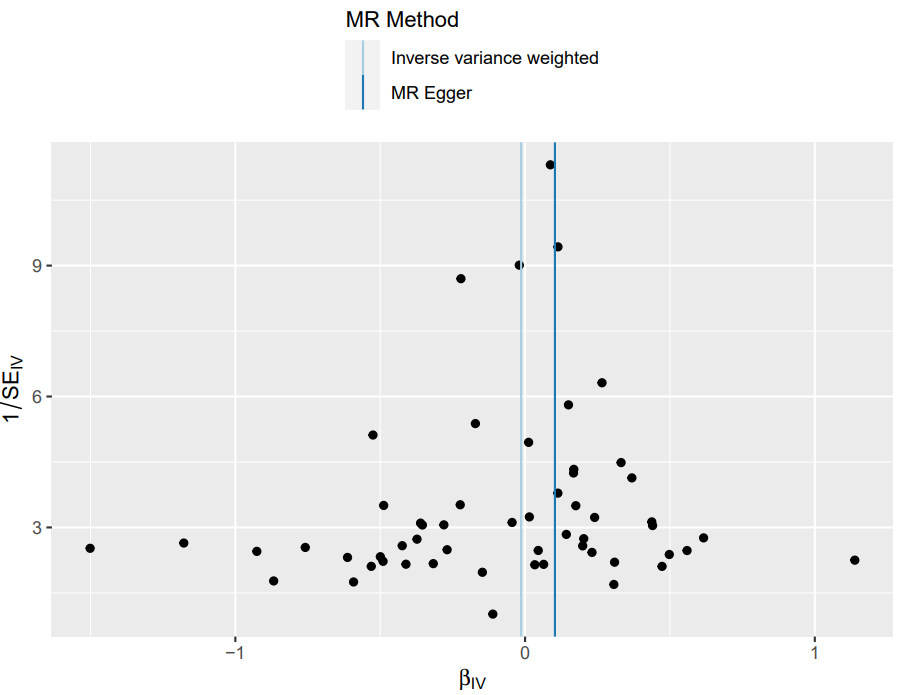

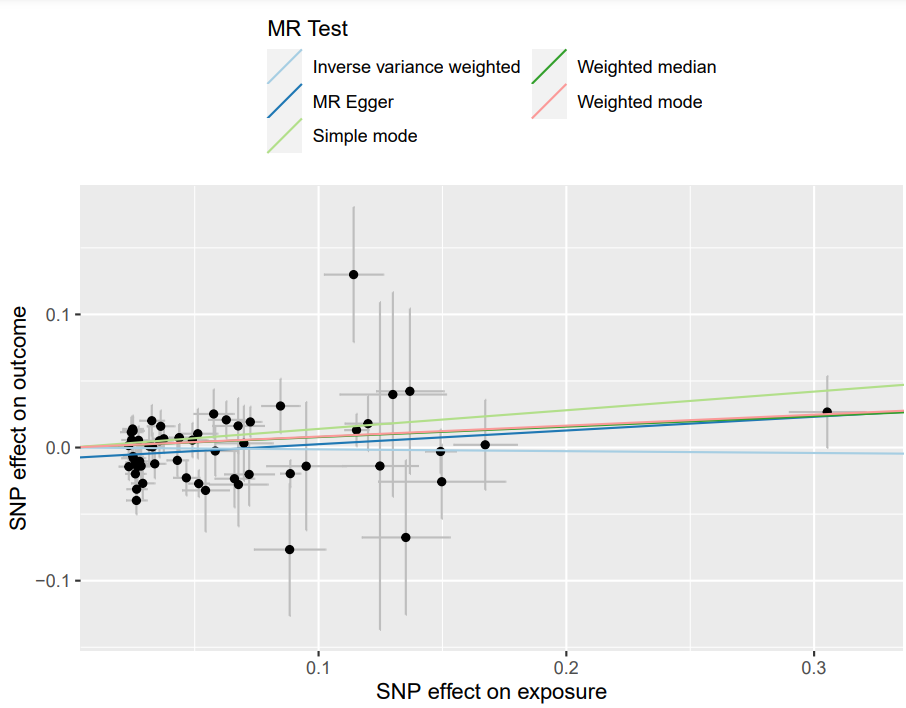
C D**

**Nonalcoholic fatty liver disease**

**
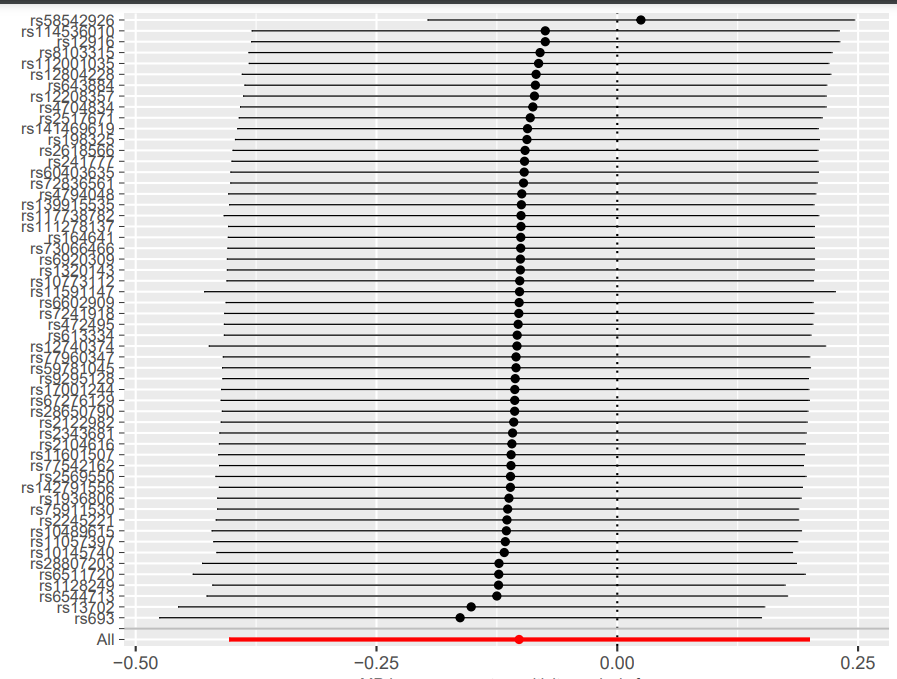

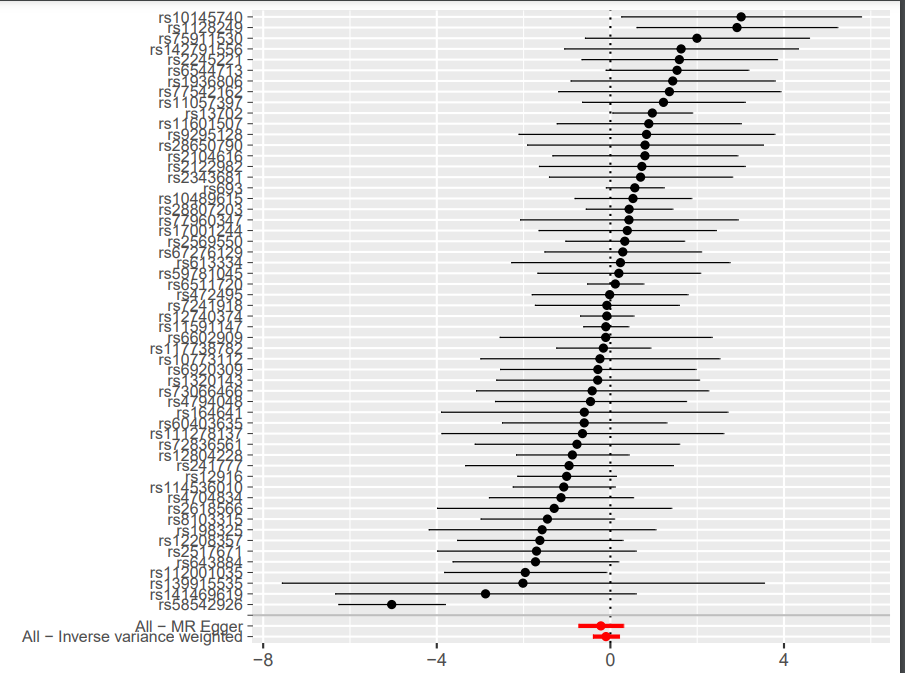
A B**

**
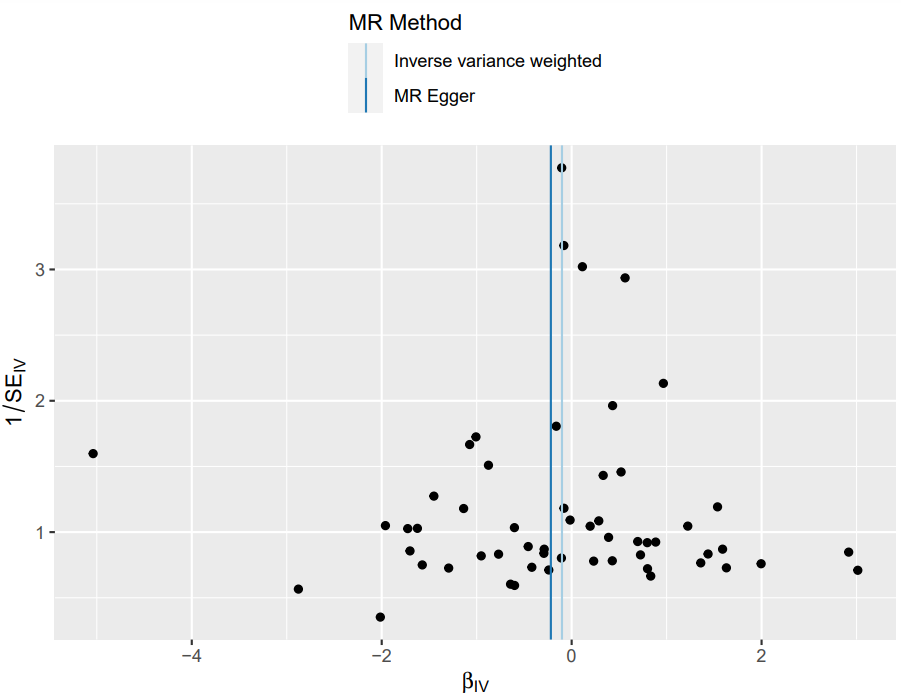

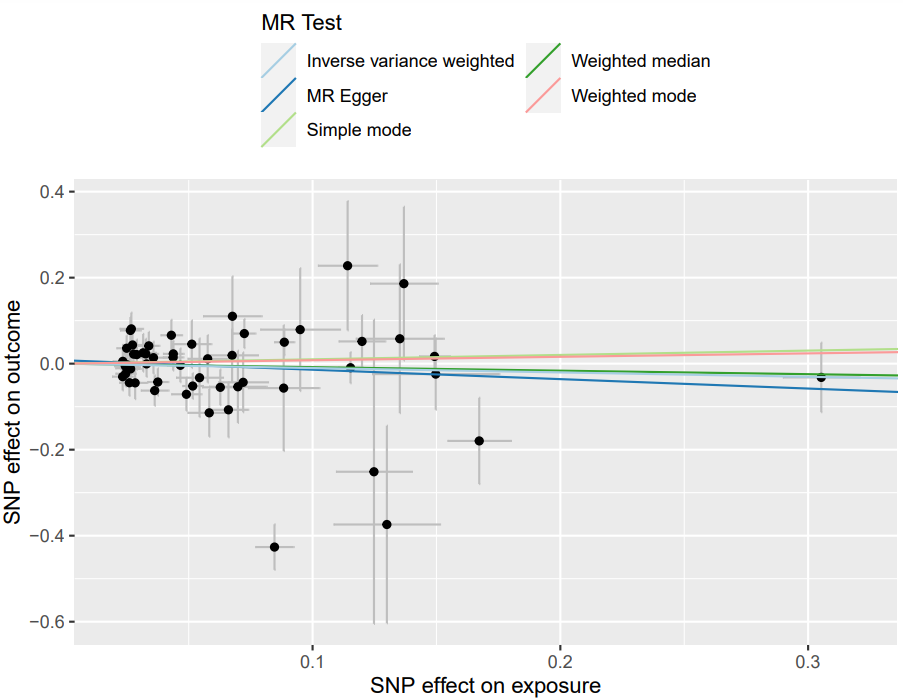
C D**

**Chronic kidney disease**

**
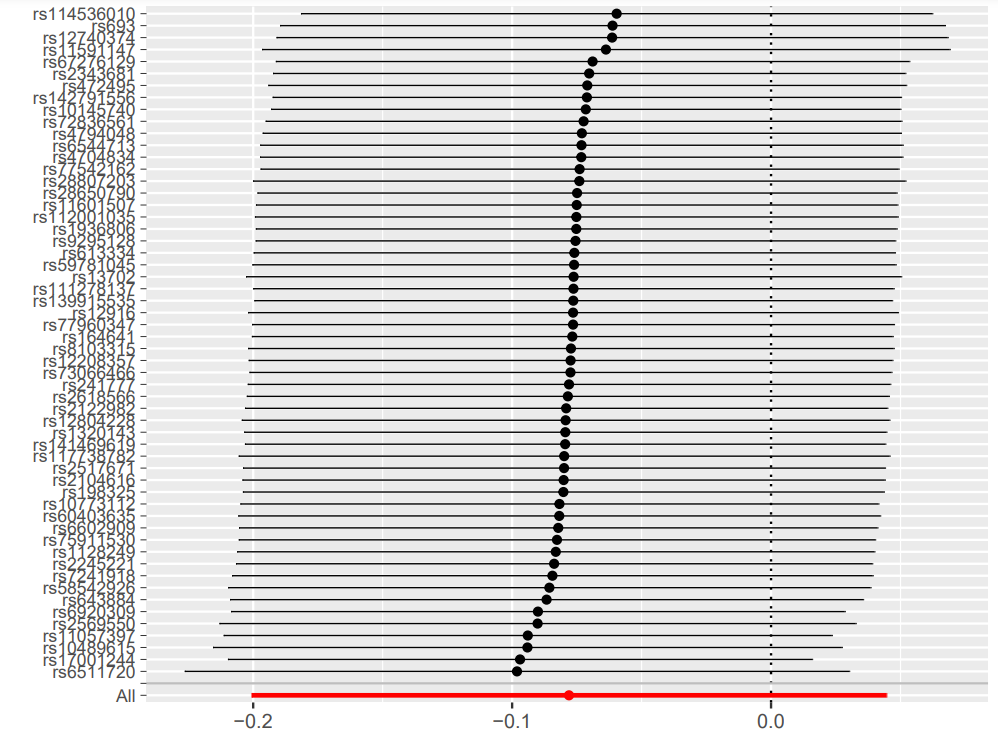

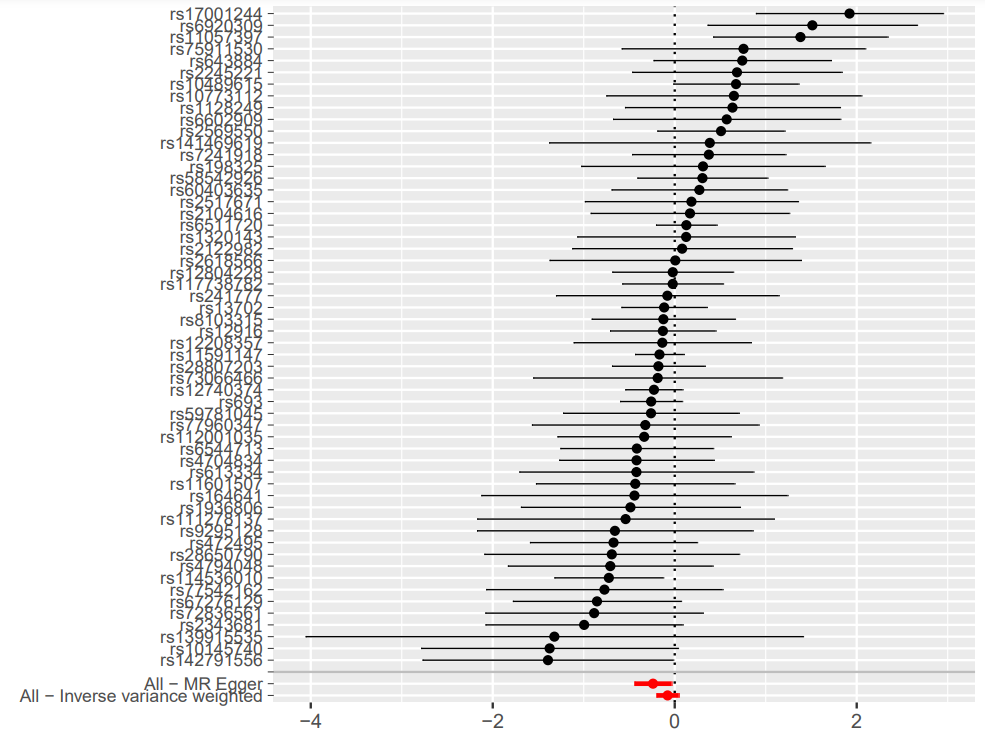
A B**

**
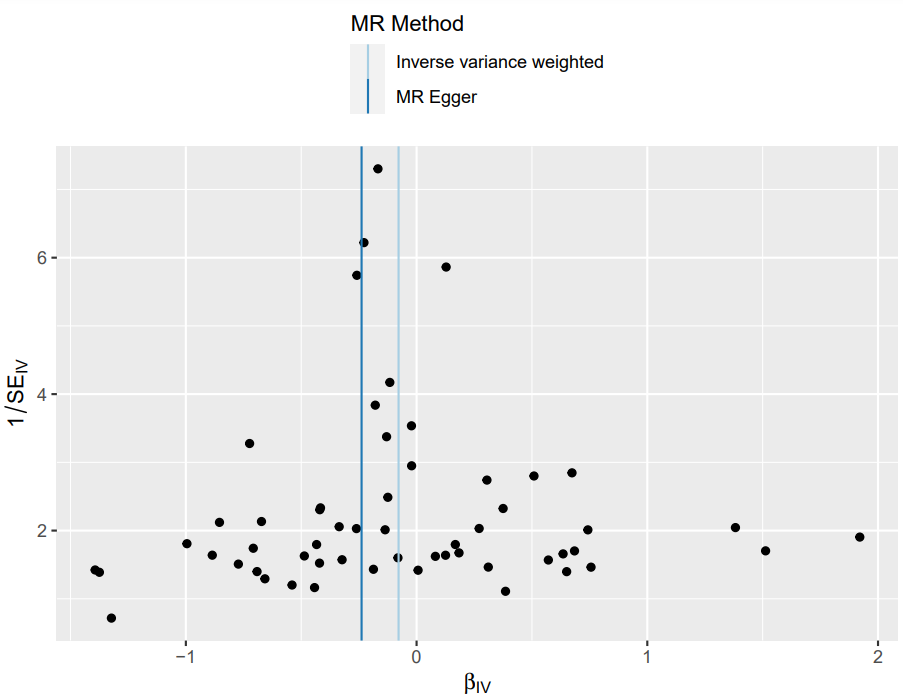

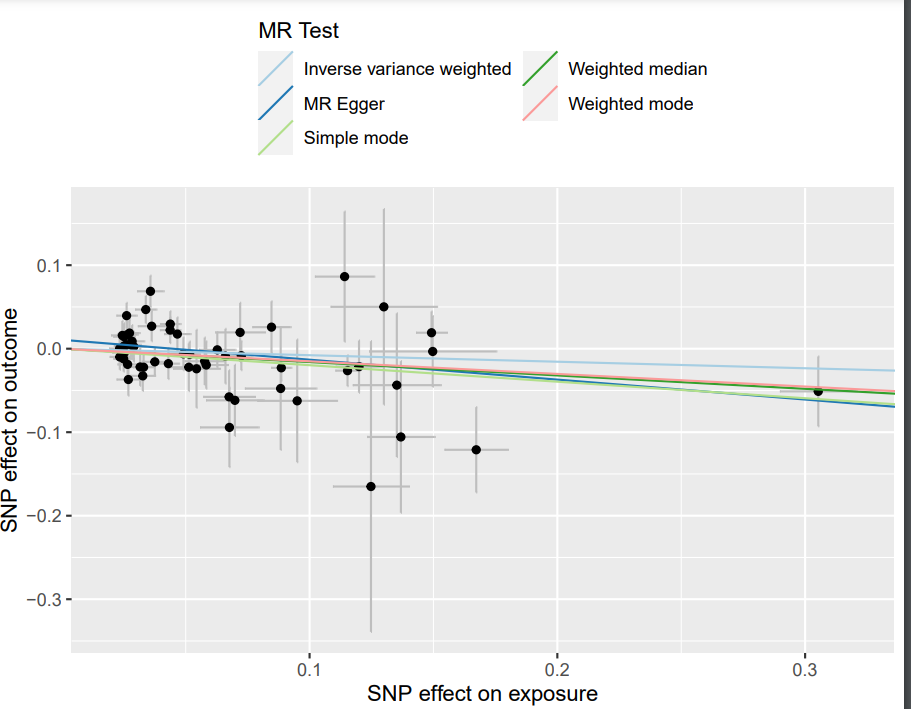
C D**

**Figure S2. Two-sample MR analyses: the casual effect of CMD risk factors on the ApoB/ApoA1 ratio. (A) The singly SNP estimated the relationship between CMD risk factors and the ApoB/ApoA1 ratio; (B) the Leave-one-out analysis in estimating the relationship between CMD risk factors and the ApoB/ApoA1 ratio; (C) The five methods of MR test; (D) The scatter diagram for showing the tendency of dispersion in the ApoB/ApoA1 ratio.**

**hemoglobin A1c**

**
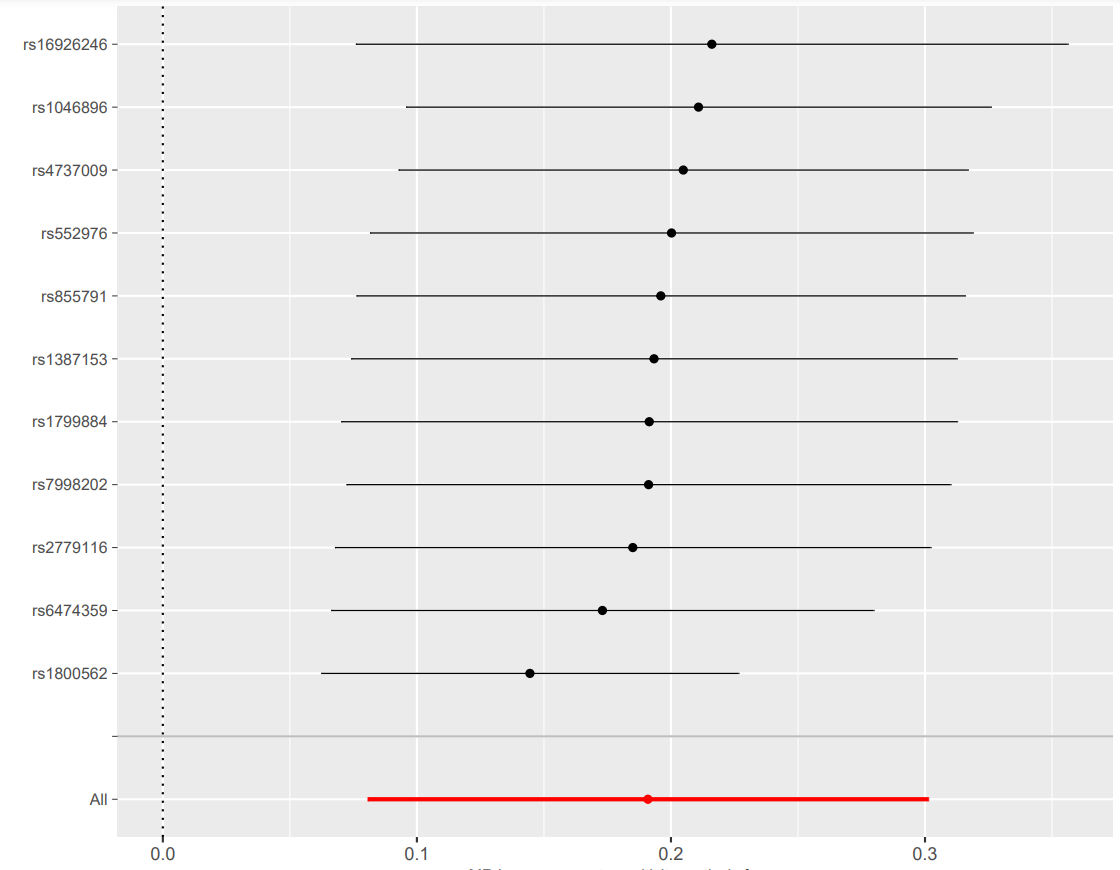

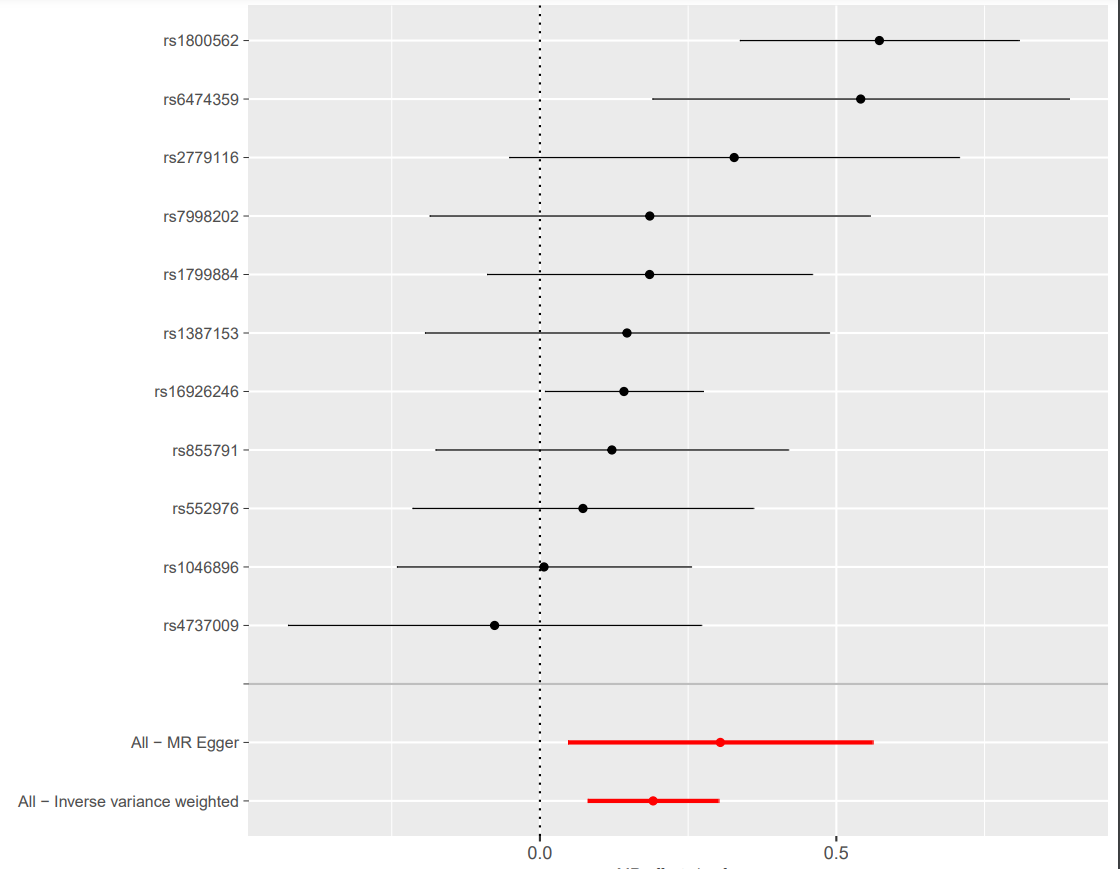
A B**

**
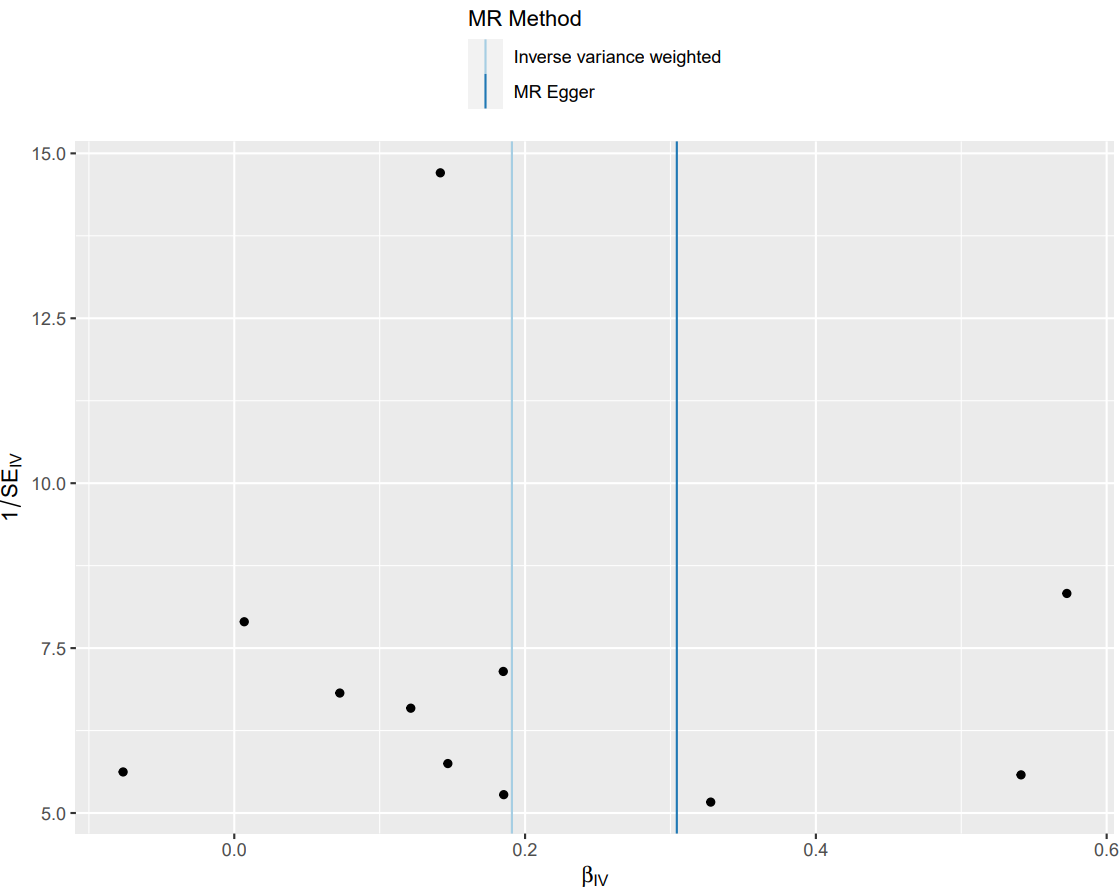

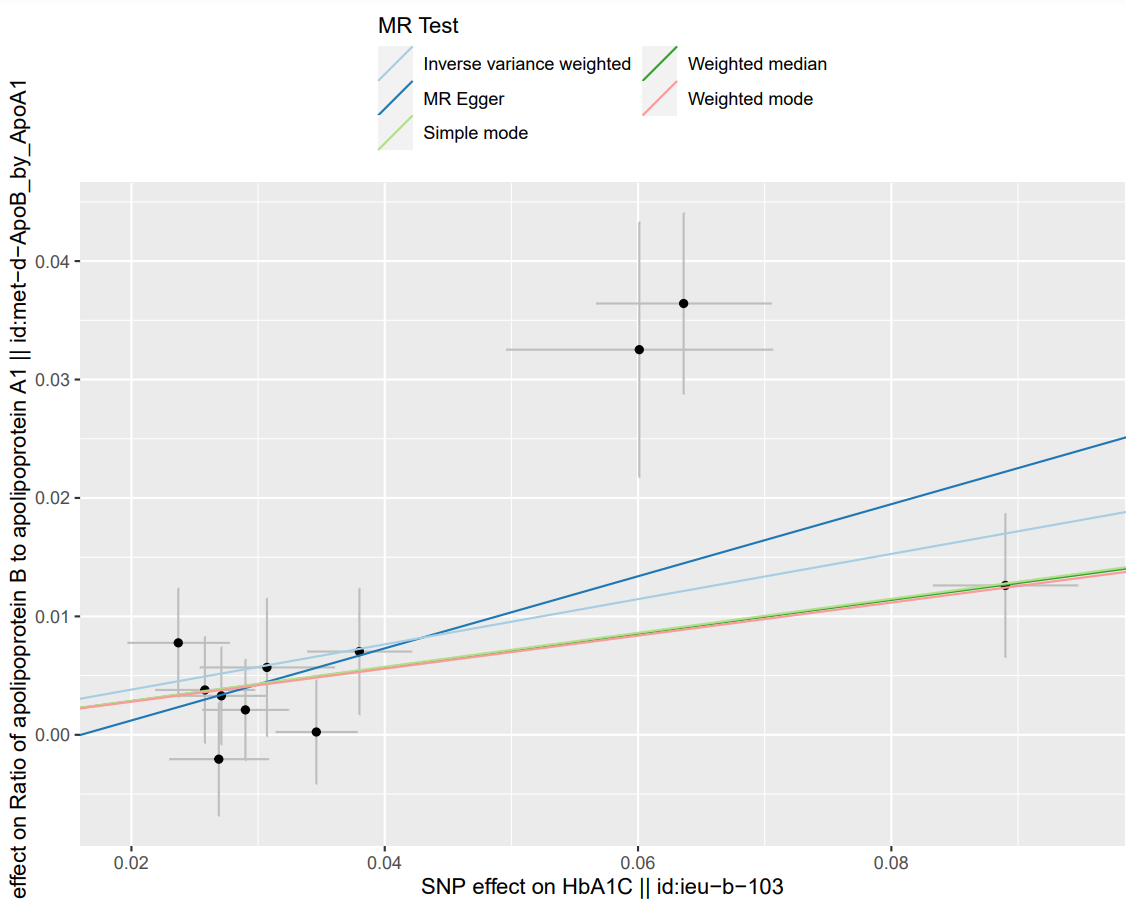
C D**

**Fasting blood glucose**

**A B**

**C D**

**Fasting insulin**

**A B**

**C D**

**Overweight**

**A B**

**C D**

**Body mass index**

**A B**

**C D**

**Waist circumference**

**A B**

**C D**

**Hip circumference**

**A B**

**C D**

**Waist-to-hip ratio**

**A B**

**C D**

**LDL cholesterol**

**A B**

**C D**

**HDL cholesterol**

**A B**

**C D**

**Coffee intake**

**A B**

**C D**

**Alcohol consumption**

**A B**

**C D**

**Alcohol intake frequency**

**A B**

**C D**

**Cigarettes smoked per day**

**A B**

**C D**

**Sedentary behavior**

**A B**

**C D**

**Insomnia**

**A B**

**C D**

**Depression**

**A B**

**C D**

**Sleep apnoea**

**A B**

**C D**

**Figure S3. Two-sample MR analyses: the casual effect of the ApoB/ApoA1 ratio on** **CMD risk factors. (A) The singly SNP estimated the relationship between the ApoB/ApoA1 ratio and CMD risk factors; (B) the Leave-one-out analysis in estimating the relationship between the ApoB/ApoA1 ratio on CMD risk factors; (C) The five methods of MR test; (D) The scatter diagram for showing the tendency of dispersion in the ratio of CMD risk factors.**

**hemoglobin A1c**

**A**  **B**

**C D**

**Fasting blood glucose**

**A B**

**C D**

**Fasting insulin**

**A B**

**C D**

**Overweight**

**A B**

**C D**

**Body mass index**

**A B**

**C D**

**Waist circumference**

**A B**

**C D**

**Hip circumference**

**A B**

**C D**

**Waist-to-hip ratio**

**A B**

**C D**

**LDL cholesterol**

**A B**

**C D**

**HDL cholesterol**

**A B**

**C D**

**Coffee intake**

**A B**

**C D**

**Alcohol consumption**

**A B**

**C D**

**Alcohol intake frequency**

**A B**

**C D**

**Cigarettes smoked per day**

**A B**

**C D**

**Sedentary behavior**

**A B**

**C D**

**Insomnia**

**A B**

**C D**

**Depression**

**A B**

**C D**

**Sleep apnea**

**A B**

**C D**
